# Application of Docking Analysis in the Prediction and Biological Evaluation of the Lipoxygenase Inhibitory Action of Thiazolyl Derivatives of Mycophenolic Acid

**DOI:** 10.3390/molecules23071621

**Published:** 2018-07-03

**Authors:** Evangelia Tsolaki, Phaedra Eleftheriou, Victor Kartsev, Athina Geronikaki, Anil K. Saxena

**Affiliations:** 1 Department of Pharmaceutical Chemistry, School of Pharmacy, Aristotle University of Thessaloniki, 54124 Thessaloniki, Greece; evantsol@yahoo.gr; 2 Department of Medical Laboratories, School of Health and Care Professions, Alexander Technological Educational Institute of Thessaloniki, 54700 Thessaloniki, Greece; 3 InterBioscreen, 119019 Moscow, Russia; vkartsev@ibscreen.chg.ru; 4 Division of Medicinal & Process Chemistry, Central Drug Research Institute, 226031 Lucknow, India; anilsak@gmail.com

**Keywords:** pharmacophore, docking, LOX, anti-inflammatory, thiazoles

## Abstract

5-LOX inhibition is among the desired characteristics of anti-inflammatory drugs, while 15-LOX has also been considered as a drug target. Similarity in inhibition behavior between soybean LOX-1 and human 5-LOX has been observed and soybean LOX (sLOX) type 1b has been used for the evaluation of LOX inhibition in drug screening for years. After prediction of LOX inhibition by PASS and docking as well as toxicity by PROTOX and ToxPredict sixteen (*E*)-*N*-(thiazol-2-yl)-6-(4-hydroxy-6-methoxy-7-methyl-3-oxo-1,3-dihydroisobenzofuran-5-yl)-4-methylhex-4-enamide derivatives with lengths varying from about 15–20 Å were evaluated in vitro for LOX inhibitory action using the soybean lipoxygenase sLOX 1b. Docking analysis was performed using soybean LOX L-1 (1YGE), soybean LOX-3 (1JNQ), human 5-LOX (3O8Y and 3V99) and mammalian 15-LOX (1LOX) structures. Different dimensions of target center and docking boxes and a cavity prediction algorithm were used. The compounds exhibited inhibitory action between 2.5 μΜ and 165 μΜ. Substituents with an electronegative atom at two-bond proximity to position 4 of the thiazole led to enhanced activity. Docking results indicated that the LOX structures 1JNQ, 3V99 and 1LOX can effectively be used for estimation of LOX inhibition and amino acid interactions of these compounds.

## 1. Introduction

Eicosanoids constitute a family of metabolic products of arachidonic acids with a wide variety of biological activities, among which are their effects on inflammation and immunity. By the action of phospholipase A2 arachidonic acid is liberated from the cell membranes and is used as a substrate for the production of biological mediators [1,2] through different pathways. The two most important pathways are the cycloxygenase and the lipoxygenase pathways, leading respectively to the production of prostaglandins (COX products) and leucotrienes (LOX products) that play a key role in inflammation. Lypoxygenases (LOX) are iron-containing enzymes with wide distribution in plants and animals. In plants they catalyze the oxidation of linoleic acid, while in mammals they do so for arachidonic acid. They all belong to the dioxygenases family, catalyzing the oxygenation of free and esterified polyunsaturated acids with (1*Z*,4*Z*)-penta-1,4 diene systems, producing the corresponding hydroperoxy derivatives [3]. LOX enzymes are categorized according to their positional specificity of arachidonic acid oxygenation [4,5]. Thus, there are six well-known LO families: 5-LOs, 8-LOs, 9-LOs, 11-LOs, 12-LOs, and 15-LOs [3,6].

**Figure 1:**
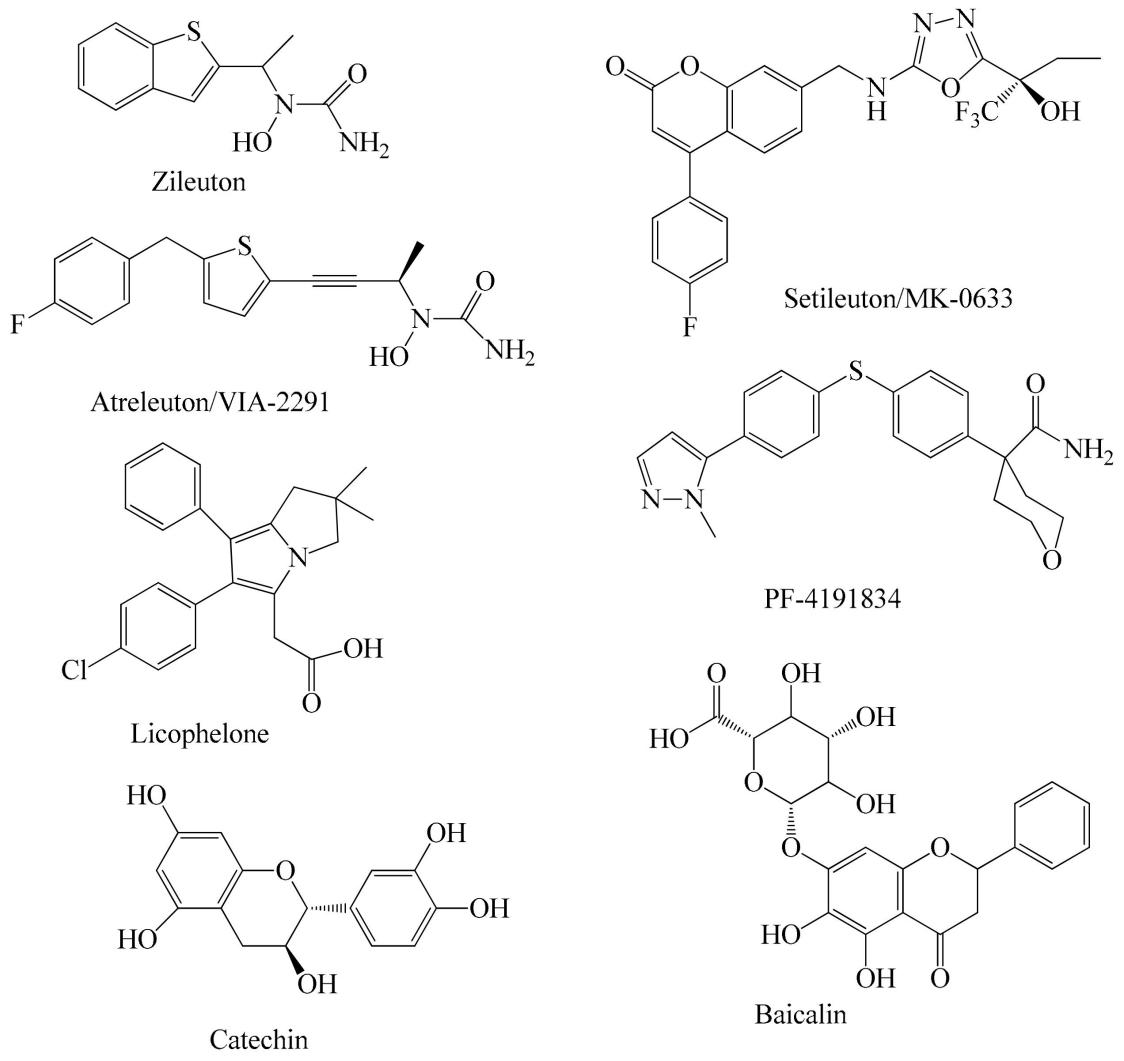
5-Lipoxygenase inhibitors under clinical development.

The mammalian 5-lipoxygenase (5-LOX) pathway (Figure 1) produces potent mediators such as leukotriene B4 (LTB4) and peptidoleukotrienes (LTC4, LTD4 or LTE4), as well as lipoxins which are also involved in inflammation. They are implicated in the pathogenesis of psoriasis [7,8], bronchial asthma [9,10] and edema formation. They are also related to leucocyte activation and adhesion to vascular endothelium [11]. There is evidence that they are among the factors involved in the damage of gastric mucosa [12]. Furthermore elevated 15-LOX activity has been correlated with atheromatic plaque formation [13,14,15,16], while association with Alzheimer’s disease progression [17] and prostate cancer development has been mentioned [18,19]. Thus, the mammalian 5-LOX [20] and 15-LOX [21] have become drug targets since their inhibitors may be used for the treatment of pathological conditions such as allergy, chronic inflammation, certain cancers and cardiovascular diseases [21,22,23,24,25,26,27].

**Figure 2:**
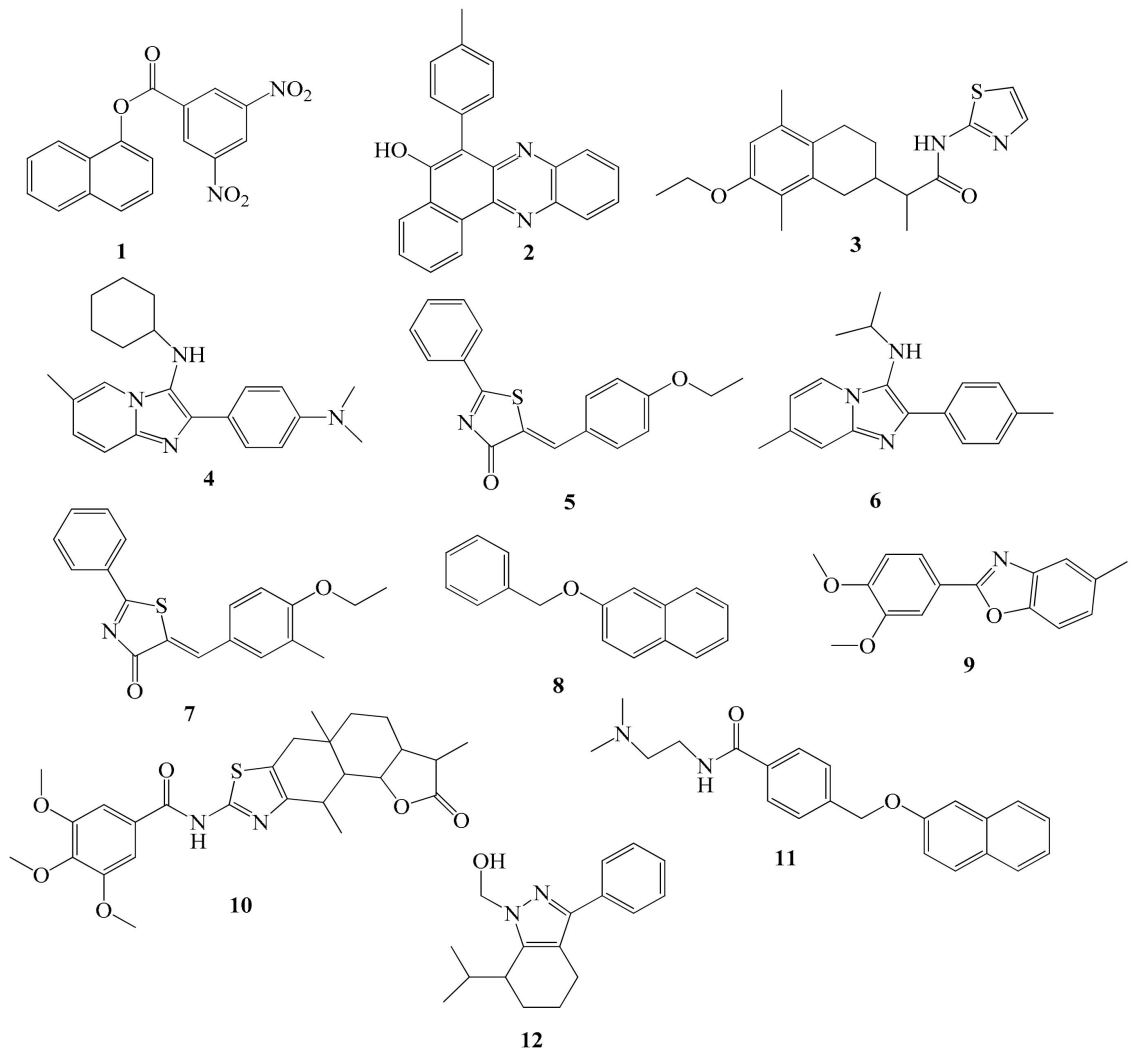
Novel 5-lipoxygenase inhibitors identified by computational screening methods.

To date only one 5-LOX inhibitor, zileuton, was approved by the FDA for the treatment of asthma, however it has been related with liver toxicity [28] and has a short half-life [29]. Furthermore its pharmacokinetic properties are unfavorable [29]. This fact, in combination with the increased indications for anti-LOX therapies has enhanced the interest of the scientific community in developing new safe and effective LOX inhibitors. These efforts are focused on both concepts, the modification of already known compounds, and the rational design of novel 5-LOX inhibitors. Figure 1 shows the structures of 5-LOX inhibitors that are under clinical development. The *N*-hydroxyurea derivative alreleuton is in phase II clinical trials for the treatment of inflammation and cardiovascular disease. [30] Setileuton (MK-0633) is a potent 5-LO inhibitor which completed phase II clinical trials against asthma [31], while licofelon (phase III) [32,33] and PF-4191834 (Phase II) [34] have been studied for knee osteoarthritis. The flavonoids baicalin and catechin are the main active components of flavocoxid, a FDA-approved product with anti-inflammatory activity also used for the treatment of osteoarthritis [35]. Furthermore, a large number of novel 5-lipoxygenase inhibitors (Figure 2) were identified using computational screening methods, such as ligand-structure-based techniques.

Arachidonic acid, the natural substrate of LOX, is a hydrophobic, flexible molecule having a carboxylate group at one end which adapts a curved conformation when interacting with the catalytic site of the enzyme. Thus, the catalytic center of 5-LOX mainly consists of the amino acids Leu368, Ile406, Ala410, Leu414, Ile415, Leu691, Val604 and Leu607, which have a hydrophobic character. The iron atom, located at the middle of the active pocket, is stabilized by coordination bonds with His367, His372, His550 and Asn554. The Phe177 also participating in complex stabilization, seems to interact with the carboxylic group of arachidonic acid [36]. Consequently, the presence of hydrophobic moieties is a mandatory characteristic of 5-LOX inhibitors, while a flexible structure, capable of adapting curved conformations, may facilitate stable complex formation as well [25,26,36,37,38]. The presence of heterocyclic rings connected by carbon chains has been observed in known LOX inhibitors such as NDGA [37].

There are many publication that refer to the LOX inhibitory activity of thiazole derivatives [26,39,40,41,42] (Figure 2) as well as phenolic compounds [43,44,45] (Figure 1). On the other hand, mycophenolic acid known for its immunosuppressing properties [46,47], has been proposed for the treatment of psoriasis, which is an inflammatory immune-mediated disease [48,49,50,51].

Nowadays a large number of organic compounds (more than 60 million) have been synthesized and can be used for biological evaluation [52]. The new pharmacologically promising compounds should meet multiple criteria, among which are, desired specific activity and lack of toxicity and serious side effects [53]. In order to search for and optimize new pharmacologically active compounds there is a need for computer approaches [54]. One very convenient computer-assisted method, based on the structure of chemical compounds is PASS [27]. In addition, docking analysis can be used if the crystallographic structure of the target molecule is known [55]. Moreover, a number of on-line programs have been developed for prediction of the toxicity of the compounds [56,57,58].

In the present study sixteen mycophenolic acid derivatives (Table 1), which combine in the same molecule a thiazole ring and a phenolic moiety with multiple molecular lengths varying from about 15–20 Å were designed. Prediction of their biological activity spectra and toxicity were performed using the PASS, PROTOX and ToxPredict programs. In addition docking analysis was also performed for prediction of inhibitory action and as a tool to facilitate SAR analysis of the experimental results. The compounds were evaluated in vitro for LOX inhibitory action. As different enzyme structures, target boxes and docking programs were used, evaluation of the best parameters for efficient docking-aided prediction of LOX inhibitors was carried out.

## 2. Results and Discussion

### 2.1. Prediction of Biological Activity Spectra of Compounds by PASS

In this paper the analysis of biological activity spectra prediction for the designed compounds was performed using the computer program PASS 2014. In silico study of compounds before the synthesis and especially biological evaluation has the advantage of avoiding time consuming and negative results. The results of PASS prediction are expressed as a list of biological activities, for which the probability of the compound to be active (Pa) or inactive (Pi) is calculated. These spectra were predicted for all designed compounds and results allow the planning of experiments. The higher the Pa value is, the less is the probability of negative results in a set of designed compounds. The prediction results revealed that for all compounds lipoxygenase inhibitory activity was predicted with Pa values in the range at 0.661–0.879, meaning that the chance of finding this activity in experiments is high; however the compounds may be close analogues to known biologically active compounds. The results of biological activity spectra prediction are presented in Table 2.

### 2.2. Prediction of Toxicity

Prediction of toxicity of a compound is a very important step in the design of new drugs. The in silico toxicity study is a more rapid and less expensive process than in vivo toxicity testing in animals, and can help to significantly reduce the number of animals used in the experimental assays. Attempts are currently being made to assess toxicity mainly by in silico models and there are several online programs that calculate the immediate toxicity of the compounds, the average lethal dose, the likelihood of carcinogenicity or mutagenicity, etc.

Two computer programs, ToxPredict and PROTOX, were used in this work [56,57,58]. The ToxPredict platform meets the requirements of the REACH legislation to use alternative methods to reduce animal experiments. Through this application, the probability of carcinogenicity of the compounds in various organisms, as well as the probability of mutagenesis is predicted using an in silico model corresponding to the Ames test. The results are presented in Table 3. The accuracy of prediction increases as the confidence values rises. In particular, reliable estimates are considered to be more reliable than 0.025. The toxicity prediction result of revealed that our compounds are not carcinogenic or mutagenic.

Another program, PROTOX [58], which predicts the average lethal dose (LD_50_) in rodents was also used. All chemical compounds can be classified into six Globally Harmonized System of Classification and Labeling of Chemicals (GHS) categories [59], depending on the toxicity of the compounds and the LD_50_ values (Table 4).

Category I: LD_50_ ≤ 5 mg/kg
Category II: 5 < LD_50_ ≤ 50 mg/kg
Category III: 50 < LD_50_ ≤ 300 mg/kg
Category IV: 300 < LD_50_ ≤ 2000 mg/kg
Category V: 2000 < LD_50_ ≤ 5000 mg/kg
Category VI: LD_50_ > 5000 mg/kg

All compounds tested were in category IV, except for compounds **13** and **14** which were in category III.

### 2.3. Docking-Aided Prediction of the Inhibitory Action

**Figure 3:**
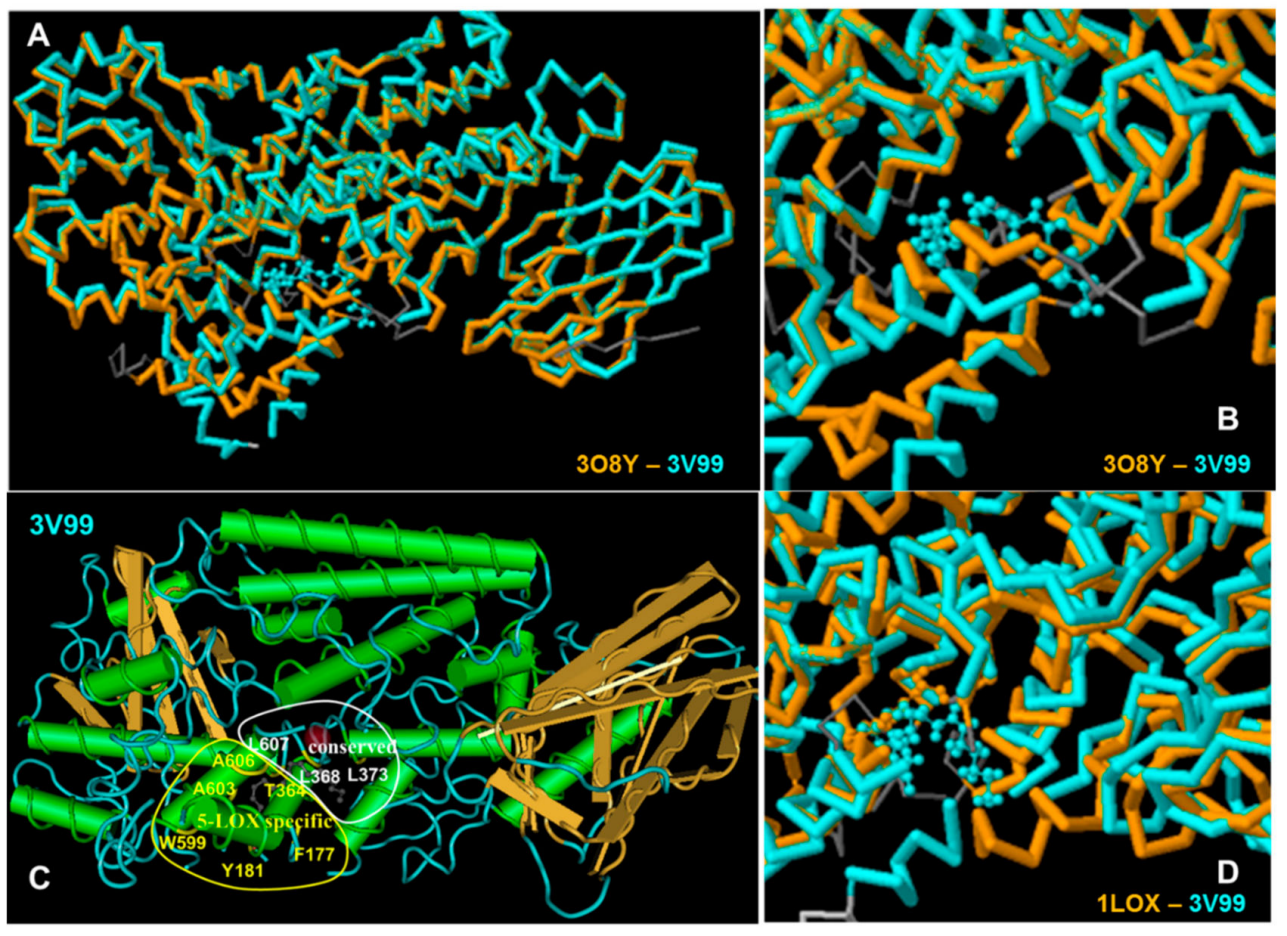
(**A**,**B**) Structure alignment between the human 5-LOX structure 3O8Y (crystalized without substrate or inhibitor) and human 5-LOX structure 3V99 (crystalized with arachidonic acid); (**C**) Amimno acids of human 5-LOX (structure 3V99), conserved in all LOX enzymes (white region), and 5-LOX specific amino acids (yellow area); (**D**) Structure alignment between the rat 15-LOX, structure 1LOX (crystalized with inhibitor) and human 5-LOX structure 3V99 (crystallized with substrate).

The X-ray crystal structures data of LOX enzymes were obtained from the Protein Data Bank. All LOX enzymes share some common characteristics at their active site, but also have differences, some of which are associated with the 5- or 15-LOX activity [36] (Figure 3). For example Leu368, Leu373, Leu414, Leu609 and Ile406 of the human 5-LOX enzyme constitute a hydrophobic cavity, conserved in all arachidonic acid catalyzing enzymes [60,61] and their analogues in rat 15-LOX are Leu362, Leu367, Ile350, Leu597 and Ile400, respectively (Figure 3C). In contrast, the amino acids Tyr181, Ala603, Ala606, His600, Thr364 and Phe177 are considered to form a 5-LOX specific region.

Although the objective of this study was the development of effective inhibitors of human 5-LOX, the soybean LOX type 1b, sLOX-1, was used for in vitro evaluation of LOX inhibitory action, as sLOX-1 is the most common soybean isoenzyme in drug screening [37,38,62,63], so both soybean and mammalian enzymes were used for the prediction of the inhibitory action of the specific compounds.

It is well known that since conformational modifications of the enzymes take place upon interaction with the substrates or inhibitors, crystallographic structures obtained by complexes of enzymes with substrates or inhibitors give better prediction results [64]. More precisely, structures derived from complexes with the substrate or competitive inhibitors are preferred in development of competitive inhibitors while structures derived from complexes with allosteric inhibitors are preferred for the evaluation of prospective allosteric inhibitors. Moreover, if crystallographic forms of complexes with different inhibitors are available, the structures with inhibitors structurally similar to the compounds of the study are preferred [65]. In the case of the LOX enzyme, all preferred inhibitors are competitive and this simplifies the process.

The crystal structure of the soybean LOX-1 (1YGE) [66], available from the Protein Data Bank was obtained from a crystallization of the pure enzyme. However, in the absence of a more suitable structure of this enzyme in the Data Bank, it (1YGE) was used for prediction, since this enzyme would be used for the biological evaluation of the inhibitors. In parallel, the crystallographic structure of a second soybean LOX, sLOX-3, was used for docking analysis due to its similarity with sLOX-1 and its availability in the Protein Data Bank in complex with inhibitor (1JNQ) [67] which is the preferred form of the enzyme.

As the real target of the prospective drugs will be the human enzyme 5-LOX the two available forms of this enzyme were also used for docking analysis, namely the stable human 5-LOX enzyme (PDB ID: 3O8Y) [61] derived from the crystallization of the pure enzyme, and the human 5-LOX structure (PDB ID: 3V99) [68] derived from a complex of the enzyme with its substrate, arachidonic acid. Although the structure 3V99 is expected to be more appropriate because of the co-crystallization with the substrate, the 3O8Y structure is preferred by some scientists because many amino acids are absent from the 3V99 structure [69] and there is a mutation of S663 to D which seems to affect the 5-LOX activity of the enzyme. Moreover, 3O8Y has adequate structural similarity with the 3V99, leaving enough space for the inhibitor to dock in the active center cavity [66]. However, 3O8Y may still not be suitable for long or bulky structures.

Since, mammalian 15-LOX has also been used in drug screening, the rat 15-LOX enzyme, crystallized with 3-(2-oct-1-ynylphenyl) propanoic acid (PDB ID: 1LOX) [70] was also chosen for this study. In addition, comparison of the results to the 5-LOX human and 15-LOX mammalian enzyme may help to determine the selectivity of the inhibitors.

**Figure 4:**
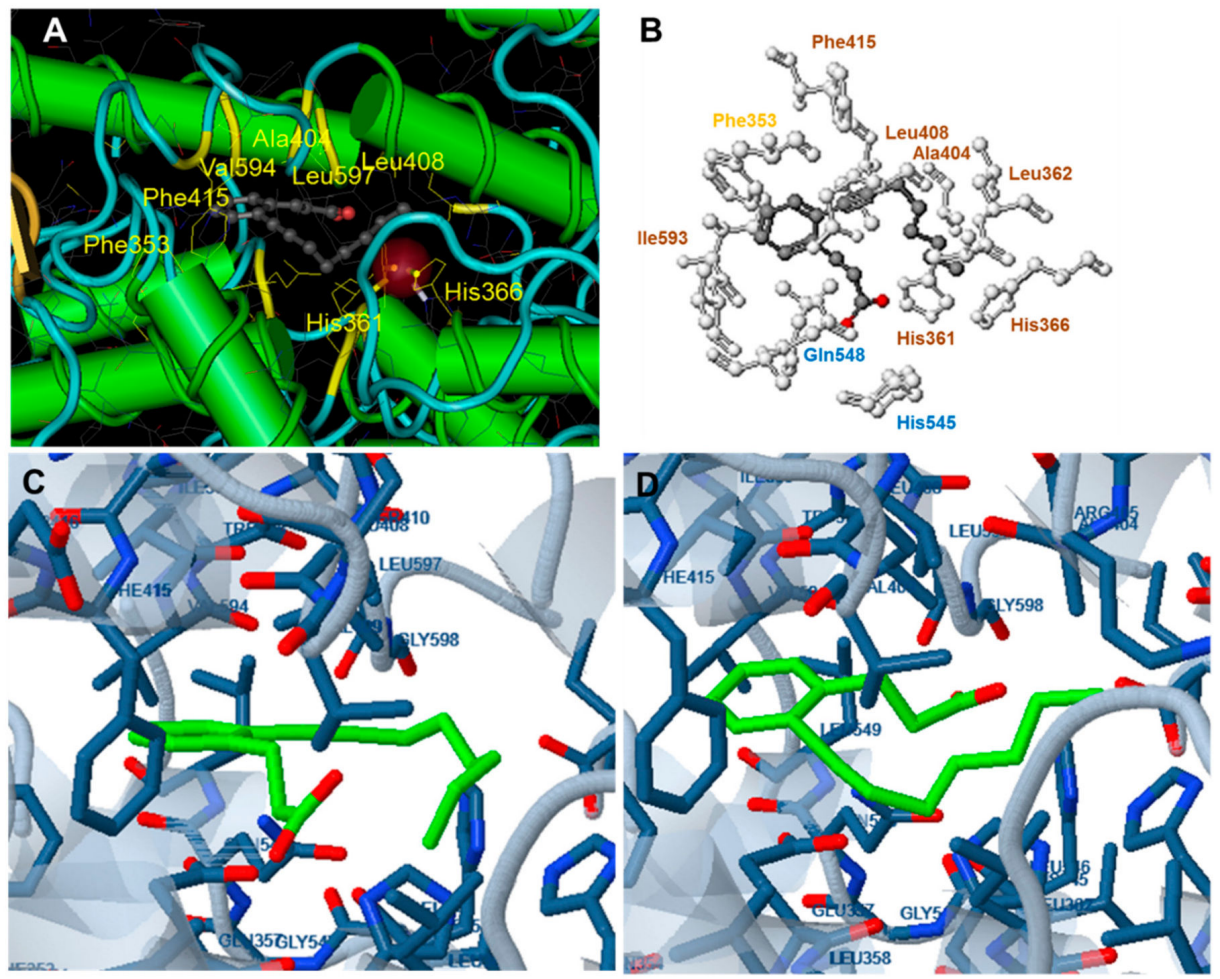
(**A**) The reference compound 3-(2-oct-1-ynylphenyl) propanoic acid (OPPA) in complex with 1LOX; (**B**,**D**) Docking of OPPA to 1LOX (target box 35). The orientation of the compound is analogue to the original complex (In B. blue: polar interactions, brown: hydrophobic interactions, yellow: pi interactions). In the complex of 1LOX with OPPA (box 35), the carboxylic group of the ligand participates in polar interactions with Gln548 and His545. Hydrophobic interactions with His361, His366, Leu362 Ala404, Leu408 and Phe415 are observed while pi-pi interactions between Phe353 and carbon atoms of the phenyl ring of the ligand also take place; (**C**) Docking of OPPA to 1LOX (target box 30). The compound occupies an opposite orientation to than in the original complex.

For docking analysis of the 1LOX structure, the docking center was kept as in the initial crystallographic structure and was in the middle of the catalytic cavity, very close to the Fe atom, which was always included at the 10 Å box around the target center. According to Feinstein et al. [65] a target box 2.9 times larger than the radius of gyration of a docking compound may improve docking efficiency. Since the length of our compounds in the lowest energy, slightly curved form, varied between 15.0 and 19.5 Å, three target boxes of 25 Å, 30 Å and 35 Å were chosen for docking studies of the compounds. In order to evaluate the target box suitability docking of the 3-(2-oct-1-ynylphenyl) propanoic acid, which is the ligand complexed with the rat 15-LOX in the 1LOX structure, was used. (Figure 4).

For docking analysis to 3V99 the docking center was also kept as in the initial crystallographic structure, in the middle of the catalytic cavity, very close to the Fe atom, and the target box was set at 30 Å. Different combinations of docking centers, and target box dimensions were used for docking analysis of the structures 1YGE and 3O8Y (see Results). In order to evaluate efficiency of docking analysis, arachidonic acid was also docked.

Docking analysis to soybean LOX-1 (1YGE) and to human 5-LOX structures 3O8Y, and 3V99 were carried out using Molecular Docking Server while docking analysis to soybean 3-LOX was performed using MolDock which combines the DE optimization techniques with a cavity prediction algorithm and a re-ranking process was applied to the highest ranked poses to further increase docking accuracy.

The best results were obtained by docking analysis of the structures that were crystallized in complex with a substrate or inhibitor (1LOX, 3V99 and 1JNQ) and are presented in Table 5. The results of docking analysis involving the enzymes which were crystallized without inhibitor (1YGE and 3V99) are presented in Table 6.

In all cases the results obtained using target boxes with different dimensions led to different ranking of the compounds according to the calculated free binding energy. In addition, substrate or ligand orientation and free binding energy of these complexes also varied according to the target box dimensions.

The best correlation of binding mode of the reference compound 3-(2-oct-1-ynylphenyl) propanoic acid (OPPA) to 1LOX with the binding mode of the compound at the crystallized complex was obtained for a target box of 35 Å (Figure 4). Hence this target box was selected for the docking of the studied compounds. Analysis of five compounds, randomly selected from those designed, revealed that four out of the five exhibited free binding energy lower than −5.50 kcal/mole which can be considered as the cut-off energy for inhibition activity [34]. Moreover, lower free binding energy (−8.29 kcal/mole) compared to the ligand OPPA (−8.09 kcal/mole) was calculated for compound **9**.

Docking of five randomly selected compounds to the human 5-LOX, 3V99 revealed that the free binding energy of the compounds varied between −6.27 kcal/mole and −10.00 kcal/mole. For four out of the five, the calculated free binding energy was lower than the energy calculated for the natural substrate of the enzyme, arachidonic acid (−6.27 kcal/mole).

The docking scores to the soybean LOX-3: 1JNQ, also indicated that these compounds can effectively inhibit the soybean LOX-3 and related LOX enzymes.

The results indicated a high probability of existence of effective inhibitors, among the designed compounds.

### 2.4. Chemistry

**Scheme 1:**
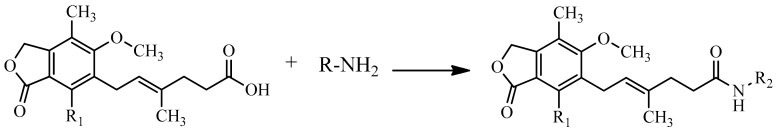
Synthesis of the title compounds. R_1_ and R_2_ are shown in Table 1.

The titled compounds were synthesized by condensation of mycophenolic acid with the corresponding amines, as shown in Scheme 1.

All compounds were characterized by melting point and ^1^H-NMR and ^13^C-NMR. The spectrosopic data were in accordance with the proposed structures.

### 2.5. Biological Evaluation

In vitro evaluation of the compounds against the widely used soybean s-LOX-1 enzyme [25,26,27] revealed that their IC_50_ values varied between 2.50 μΜ and 156.25 μΜ (Table 5). It was observed that differentiation of both R_1_ and R_2_ substituents significantly affects inhibitory action.

Comparison of the hydroxyl derivatives **1** (IC_50_ = 26.25 µM) and **2** (IC_50_ = 22.50 µM) with the methoxy- analogues **15** (IC_50_ = 96.25 µM) and **16** (IC_50_ = 90.00 µM) respectively differing in the R_1_ substituent of the benzyl ring, revealed that the OH group strongly favors inhibitory action compared to the OMe- group.

Compounds 1–14 which belong to the same group concerning the R_1_ substituent (R = OH), are ranked as follows: 9 > 2, 5 > 13 > 1 > 6 > 4, 14 > 3 > 12 > 7 > 8, 10 > 11. The R_2_ substituent consists of a non-substituted or a substituted thiazol-2-yl ring. Addition of a methyl group at position 5 of the ring (compound 2) results in a reduced IC_50_ value (22.50 µM) compared to the non-substituted compound 1 (26.25 µM). On the other hand, introduction of a methyl (compound 4) or an ethyl acetate group (compound 6) at position 4 of the ring results in decreased activity, with IC_50_ values of 31.25 µM and 30.00 µM, respectively. The introduction of a second small hydrophobic methyl-substituent at position 5 of compound 4 leads to an even less active compound, 3 (IC_50_ = 43.75 µM). Interestingly, the presence of the long, relatively hydrophilic ethyl formate substituent at position 5 of a 4-methyl thiazol-2-yl moiety seems to counteract the positive effect of the 4-methyl substituent, leading to compound 5 with an IC_50_ = 22.50 µM.

Interestingly the presence of a pyridine ring at position 4 of the thiazol-2-yl moiety had a different effect depending on the position of the nitrogen in the ring. The pyridine-4-yl and pyridine-3-yl derivatives, **7** and **8**, showed decreased activity with IC_50_ values 78.75 µM and 90.00 µM, respectively, while the pyridine-2-yl derivative, **9**, exhibited the best activity with an IC_50_ value of 2.50 µM, underlining the great importance of the presence of the electronegative nitrogen atom close to position 4 of the thiazolyl moiety.

Addition of hydrophobic rings at position 4–5 of the thiazol-2-yl ring did not favor the activity. The difference in IC_50_ values of the derivatives varied between 25.00 µM (**13**) and 90.00 µM (**10**) with a ranking as follows: cycloheptene > cyclopentene > cyclohexene > benzene. The size and bending capacity of the ring may be related to the different activities of the compounds. Methoxy-substitution of the benzene ring resulted in compound **11** with an even lower activity (IC_50_ value of 156.25 µM).

### 2.6. Docking Analysis-Assisted Justification of Inhibitory Action

The docking studies revealed that docking results do not always correlate with the IC_50_ values (Table 5) of compounds. However, the best docking scores for all tested enzyme structures (soybean LOX-3: 1JNQ, as well as also for the human 5-LOX: 3V99 and the rat 15-LOX: 1LOX) were obtained for compound **9**, with the lowest IC_50_ value.

**Figure 5:**
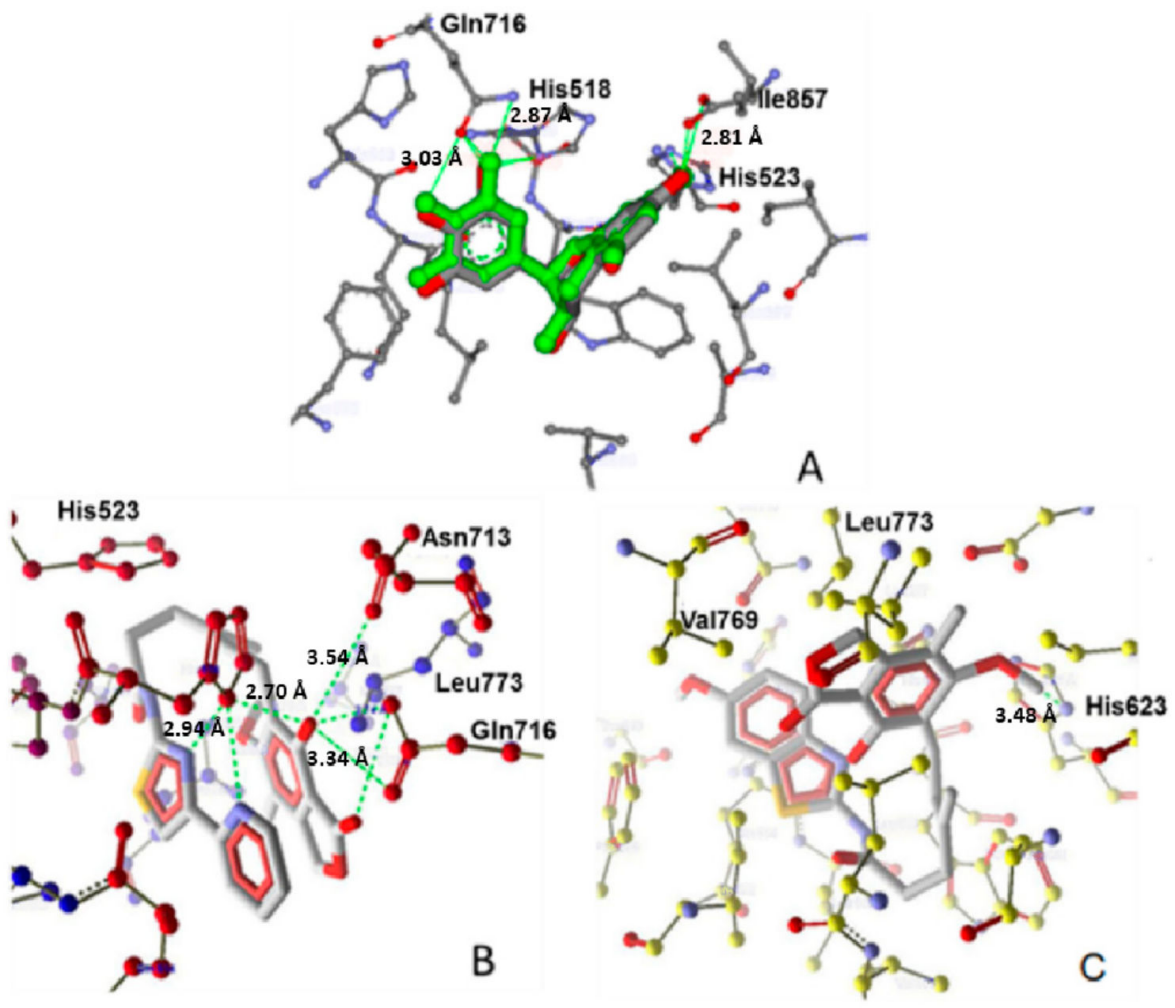
(**A**) Alignment and molecular interactions of co-crystallized epigalocatechin and redocked epigalocatechin in the LOX binding site PDB ID 1JNQ; (**B**) Interactions of the most active compound **9** with LOX protein PDB ID 1JNQ; (**C**) Molecular interactions of the least active compound **11** with LOX protein PDB ID 1JNQ. Hydrogen bonds are shown in green.

**Figure 6:**
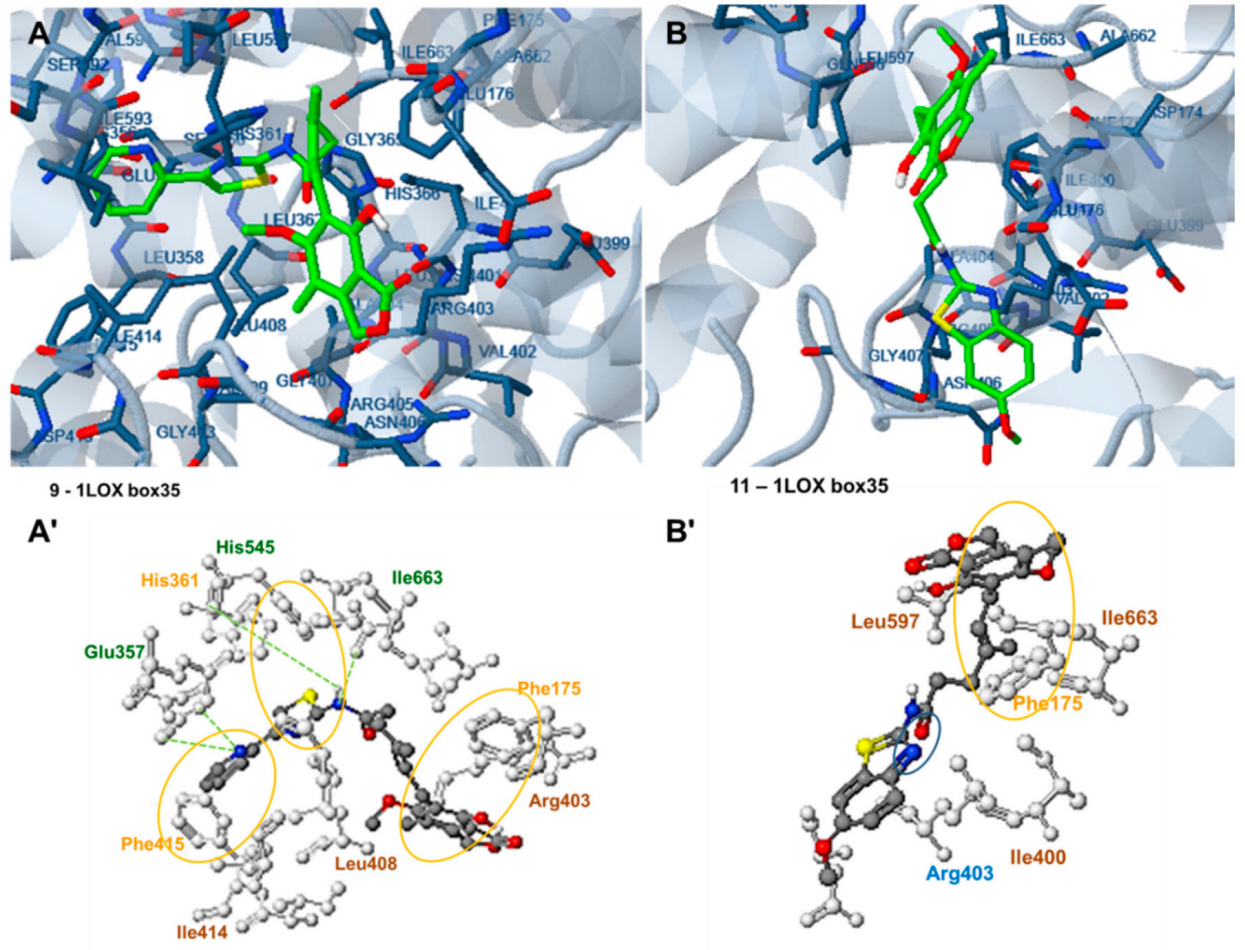
Docking analysis of the most active (IC50 2.5 µM) compound **9**, (**A**,**A**′) and the less active, (IC_50_ 156.25 µM) compound **11** (**B**,**B**′) to the active site of the rat 15-LOX structure PDB ID: 1LOX (target box 35). Green: hydrogen bond interactions, Blue: polar interactions, yellow: pi interactions, brown: hydrophobic interactions.

**Figure 7:**
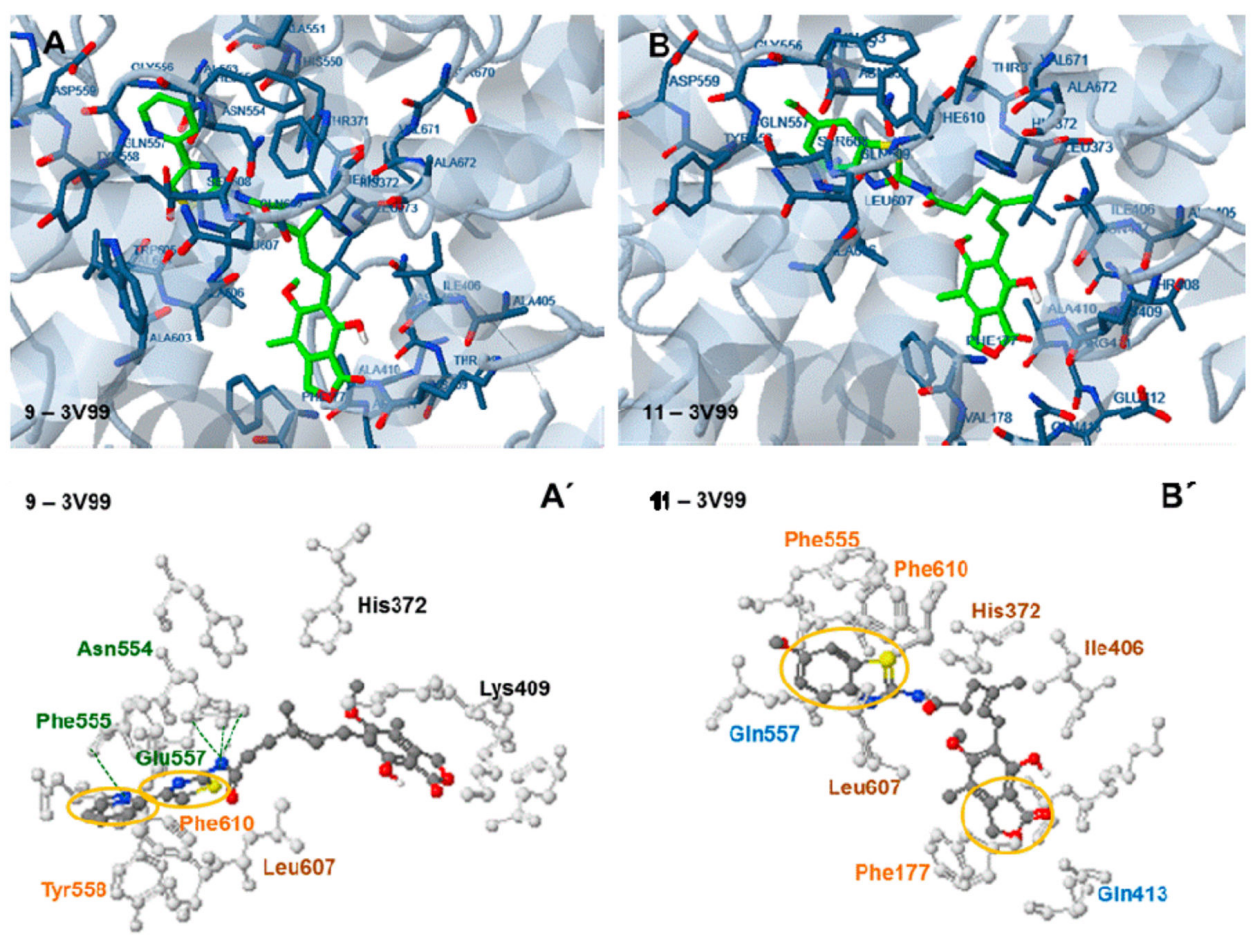
Docking analysis of the most active (IC50 2.5 µM) compound, **9**, (**A**,**A**′) and the less active, (IC_50_ 156.25 µM) compound **11** (**B**,**B**′) with the active site of the human 5-LOX structure PDB ID: 3V99 (target box 30). Green: hydrogen bond interactions, blue: polar interactions, yellow: pi interactions, brown: hydrophobic interactions.

Analysis of the docking of compound **9** to all three enzymes (soybean LOX-3: 1JNQ, rat 15-LOX: 1LOX and human 5-LOX: 3V99 (Figure 5B, Figure 6A,A′ and Figure 7A,A′, respectively) showed a strong involvement of hydrogen bonding and pi interactions in complex stabilization. The N atom of the pyridine ring participates in hydrogen bond interactions in all cases, while the pyridine and thiazolyl- rings participate in pi interactions. A different orientation and lack of hydrogen bonds are observed in the case of the less active compound **11** in all enzyme structures (Figure 5C, Figure 6B,B′ and Figure 7B,B′).

More precisely all compounds exhibited good binding affinity towards the soybean LOX-3: 1JNQ (Table 1). Compound 9 showed comparatively higher binding affinity represented by its Moldock and re-rank scores, which were better than the reference compound, and a significantly lower IC_50_ value. As shown in Figure 3B, the compound is placed in a bended conformation at the active site of the enzyme interacting with the amino acids His518, Leu773, Gln716 and Asn713. Hydrogen bonds are formed between the N of pyridine and thiazolyl rings and His518 and between OH substituent of the benzene ring and carbonyl group of the furanone ring and the amino acids Asn713 and Gln716. Moreover, “pi-pi” interactions with the residues Phe576 and Trp519 are also observed. Involvement of amino acids such as Asn713 and His518 in hydrogen bond formation as well as pi-pi interactions have been mentioned in complex stabilization of other ligands to 1JNQ, such as the reference ligand, epigalocatechin, presented in Figure 4A. In contrast only one hydrogen bond with His623 and no pi-pi interactions were observed in case of the less active compound 11 (Figure 5C).

Docking analysis of the rat 15-LOX structure PDB ID: 1LOX (target box 35) revealed that compound 9 is also placed in the active center of the enzyme in a curved conformation with the pyridine-2-yl-5-thiazol-2-yl moiety oriented in the vicinity of Leu408, Ile414, Phe415, Glu357, His361, His545 and Ile663, and the 1,3-dihydroisobenzofuran-2-one moiety placed near Phe175 (Figure 6A,A′). Two hydrogen bonds are formed between the H atom of the amide group linked to the thiazolyl- moiety and the *O* atoms of the peptide bonds of Ile663 and His545, while two more hydrogen bonds are formed between the *N* atom of the pyridine ring and Glu357. Phe415, His361 and Phe175 participate in pi-pi interactions with pyridine, thiazolyl and benzene rings respectively. In addition, hydrophobic interactions with Ile414, Leu408 and Arg403 also contribute to complex stabilization.

The less active compound, 11 has a different orientation in the 1LOX active site, with the 1,3-dihydrobenzofuranone moiety placed near Leu597, Ile663 and Phe175, with which it participates in a π-π interaction. However, the benzothiazolyl moiety is now placed near Arg403 and Ile400. No hydrogen bond formation and fewer π-π interactions are observed in this case, which explains the higher free binding energy of this complex (−6.71 kcal/mole compared to the −8.29 kcal/mole of compound 9).

Docking of compound **9** to human 5-LOX: 3V99, revealed that the enzyme was oriented with the thiazolyl moiety towards Leu607, Phe610, Tyr558, Asn 554, Phe555 and Glu557 and the dihydroisobenzofuranone moiety towards Lys409 (Figure 7A,A’). Three hydrogen bonds are formed between the H and N atom of the amide group linked to the thiazolyl moiety and the side chain of Gln557 and Asn554 and a fourth one is formed between the N atom of the pyridine ring and the peptide bond of Phe555. π-π interactions between the pyridine and thiazolyl rings and the amino acids Phe558 and Phe610 also participate in complex stabilization. The observed interactions indicate a high affinity of the compound with the active site of the human 5-LOX enzyme, which will be the real target of the prospective inhibitors. This explains the low calculated free binding energy of the compound to 3V99 (−10.00 kcal/mole) and supports the idea that compound **9** can effectively inhibit the human enzyme.

A more bent conformation is adapted by compound 11 (Figure 7B,B′) with the thiazolyl moiety placed in the same area of the enzyme as in the case of compound 9, and the dihydro-isobenzofuranone moiety placed towards Phe 177. No hydrogen bond is observed in this case. However π-π interactions are formed between the benzothiazolyl moiety and the amino acids Phe555 and Phe619 and between the furanone ring and the amino acid Phe177. The relatively weaker interactions observed justify the higher free binding energy of this compound (−7.49 kcal/mole).

**Figure 8:**
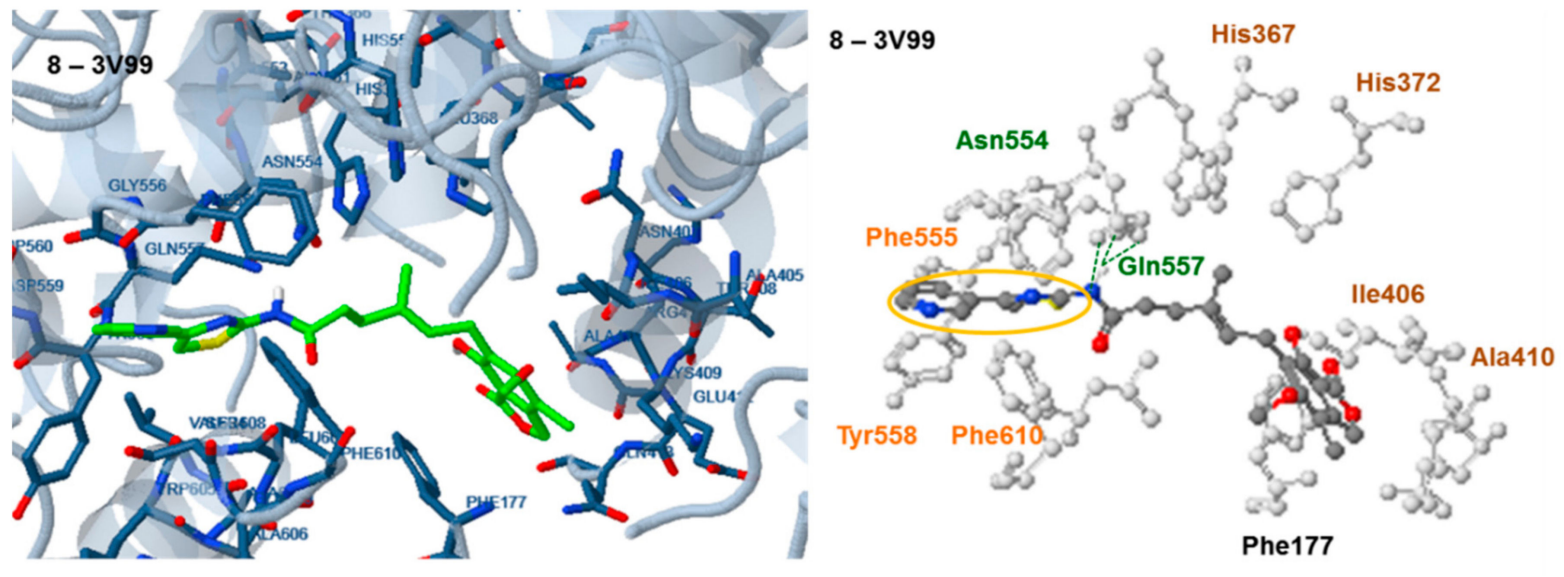
Docking analysis of compound, **8**, with the active site of the human 5-LOX structure PDB ID: 3V99 (target box 30). Green: hydrogen bond interactions, yellow: pi interactions, brown: hydrophobic interactions.

A higher free binding energy (−9.01 kcal/mole) was calculated for the pyridine-3-yl derivative 8 compared to the pyridine-2-yl derivative 9. According to docking (Figure 8) the different position of the N atom in pyridine ring results in inability to form a hydrogen bond with Phe555. Three hydrogen bonds are now formed between the H atom of the amide group linked to the thiazolyl moiety and the O atoms of Gln557 and Asn554 while pi-pi interactions between the pyridine and thiazolyl ring and Phe555, Tyr558 and Phe610 also participate in complex stabilization.

### 2.7. Evaluation of Docking Analysis Efficiency

In general, the soybean sLOX structure 1YGE and the human 5-LOX structure 3O8Y, where the enzyme was crystallized without substrate or inhibitor, were not suitable for docking analysis of these compounds, probably due to their size. Structure alignment of the two human 5-LOX structures, 3O8Y (crystallized without substrate) and 3V99 (with substrate), clearly indicates the increased volume of the active site in case of 3V99 (Figure 3A,B).

**Figure 9:**
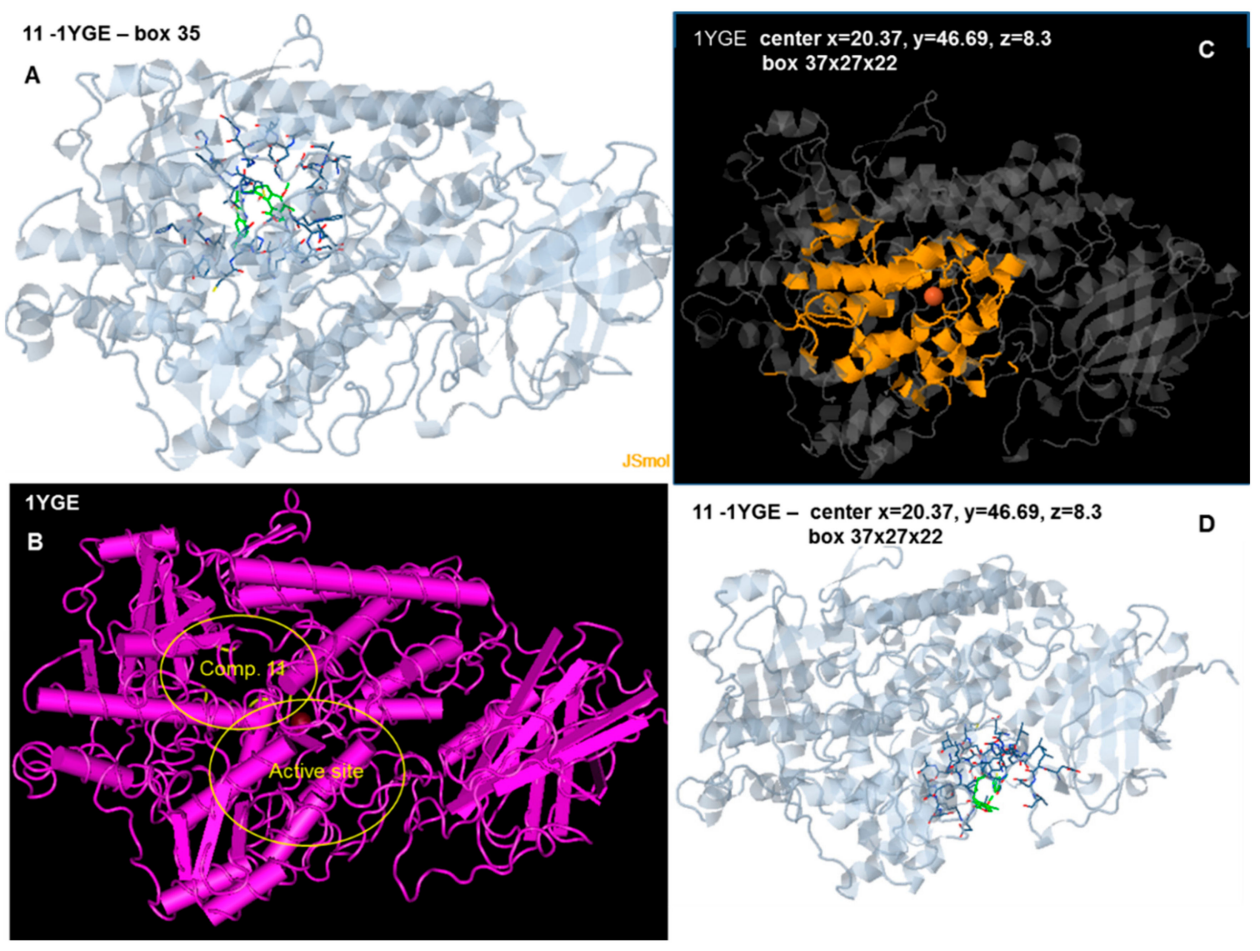
(**A**,**B**) Docking of compound **11** to the soybean LOX-1 structure 1YGE, target center: x = 26.37 Å, y = 42.69 Å, z = 12.36 Å, target box 35 × 35 × 35Å. The target box enables the compound to dock preferentially at the upper left corner, behind the active site. The interacting amino acids are marked in yellow at Figure 7B; (**C**) Structure 1YGE with the target box in yellow (center x = 20.37 Å, y = 46.69 Å, z = 8.3 Å, box 37 × 27 × 22 Å); (**D**) Docking of compound **11** to 1YGE (center x = 20.37 Å, y = 46.69 Å, z = 8.3 Å, box 37 × 27 × 22 Å).

For docking analysis of the structure 1YGE, the docking center was kept as in the initial crystallographic structure and was in the middle of the catalytic cavity, very close to the Fe atom, which was always included at the 10 Å box around the target center (target center: x = 26.37, y = 42.69). According to Feinstein et al. [66] a target box 2.9 times larger than the radius of gyration of a docking compound may improve docking efficiency. Since the length of our compounds in the lowest energy varied between 15.0 and 19.5 Å, three target boxes of 20 × 20 × 20 Å, 30 × 30 × 30 Å and 35 × 35 × 35 Å were chosen for docking analysis of the compounds. A positive free binding energy was calculated for the box at 20 Å (Table 6). Negative values for the free binding energy were obtained for boxes 30 Å and 35 Å. However, the extended docking target and the constrained area at the active center of the enzyme enabled attachment of the compounds at incorrect docking sites in some cases. Figure 9A,B show the binding site of compound **11**, and the distance between this and the active center of the enzyme.

Since the active site is not sufficiently included in a cubic box centered on the Fe atom several changes were made to the center and box dimensions to include all interacting amino acids and to exclude the non-interacting ones from the target area (Table 5, Figure 9C). This gave better results (Figure 9D), but although the target area was closer to the active site, some compounds failed to bind into the active center cavity, but nevertheless were attached to amino acids around the entrance of the cavity. This was probably due to the restricted cavity and entrance size, which are enlarged in enzymes crystallized with their substrates or inhibitors.

Moreover the insufficiency of this structure as a tool for docking analysis for these compounds is shown by the lack of correlation between the calculated free binding energies and the IC_50_ values of the compounds in most cases (Table 5). Since, the 1YGE structure corresponds to the soybean enzyme used for the biological evaluation, successful docking analysis should effectively predict the IC_50_ values of the compounds.

Although the human 5-LOX 3O8Y has been previously used by our team for docking evaluation [28], and the structure is proposed for drug screening by other scientists [29], the results obtained for some of the compounds of this group were not satisfactory.

**Figure 10:**
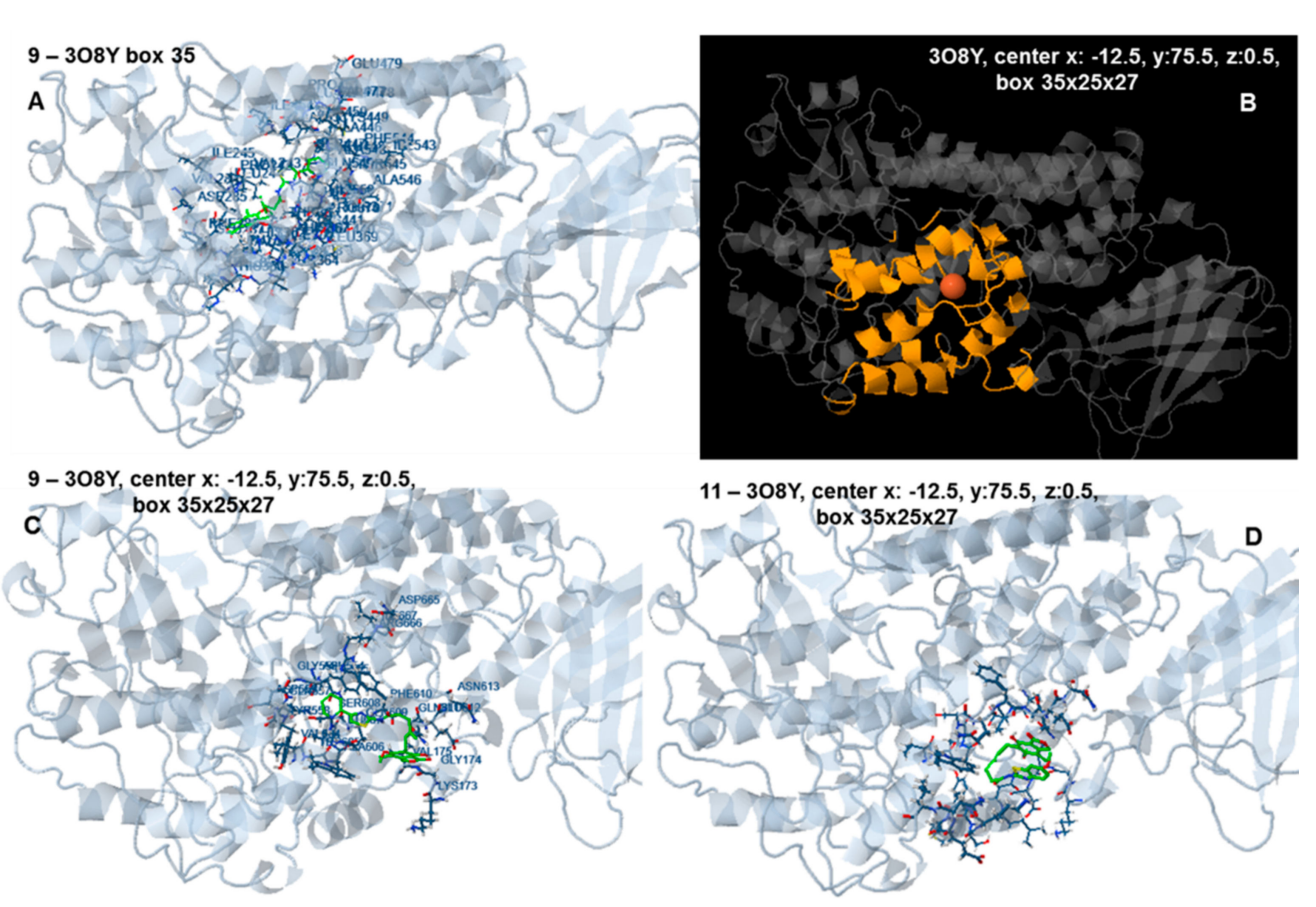
(**A**) Docking of compound **9** to the human 5-LOX structure 3O8Y, target center: x = 8.59 Å y = 22.65 Å, z = −1.02 Å, target box 35 × 35 × 35 Å. The target box enables the compound to dock preferentially at the upper left corner, behind the active site; (**B**) Structure 3O8Y with the target box in yellow (center x: −12.5 Å, y: 75.5 Å, z: 0.5 Å, box 35 × 25 × 27 Å); (**C**) Docking of compound **9** to 3O8Y (center x: −12.5 Å, y: 75.5 Å, z: 0.5 Å, box 35 × 25 × 27 Å); (**D**) Docking of compound **11** 3O8Y (center x: −12.5 Å, y: 75.5 Å, z: 0.5 Å, box 35 × 25 × 27 Å).

**Figure 11:**
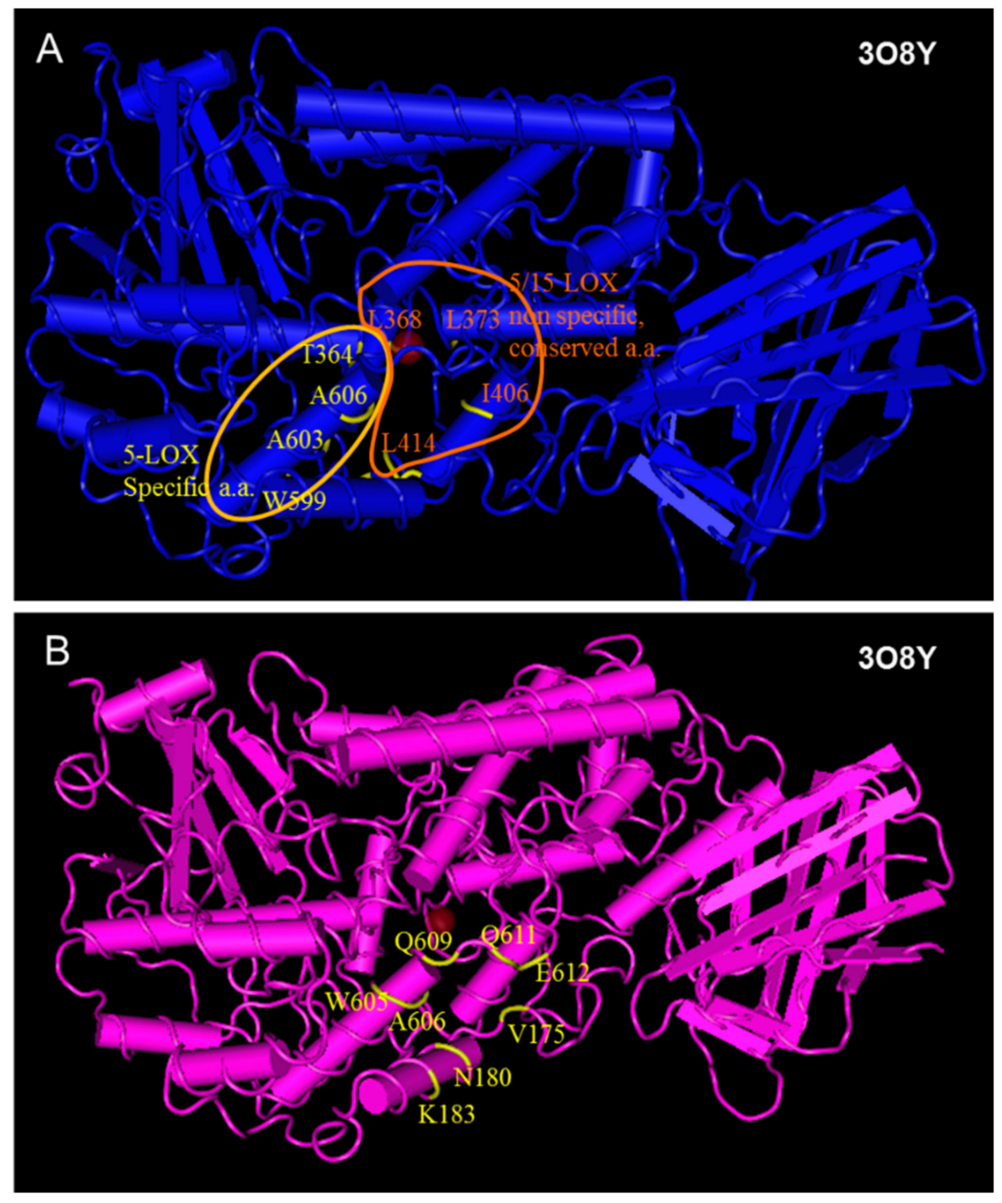
(**Α**) Conserved and 5-LOX specific amino-acids which participate in interactions with substrates and inhibitors at the active site of 3O8Y, indicated in yellow (some of them barely seen because they are hidden inside the cavity); (**B**) Amino acids interacting with compound **11** in the preferred docking pose to 308Y (center x: −12.5 Å, y: 75.5 Å, z: 0.5 Å, box 35 × 25 × 27). The interacting amino acids are located at the external area of the molecule.

In an analogous way with 1YGE preferential docking of the compounds to other sites in the neighborhood or around the entrance of the active site was observed, probably because of the large volume and length of the compounds and the restricted area of the active site. Docking of compound **9** to the human 5-LOX structure 3O8Y at 35 × 35 × 35 Å target box (target center: x = 8.59 Å y = 22.65 Å, z = −1.02 Å), and when the target box was set at 35 × 25 × 27 Å (target center: x: −12.5 Å, y: 75.5 Å, z: 0.5 Å), are shown in Figure 10A,C, respectively. Docking of compound **11** to 3O8Y (center x: −12.5, y: 75.5 Å, z: 0.5 Å, box 35 × 25 × 27 Å) is shown in Figure 10D. The binding site of the compound, although close to the active site cavity, is located at the external area of the molecule (Figure 11), so the second available human 5-LOX structure, 3V99, was preferably used for inhibition evaluation.

Docking to the human 5-LOX structure, 3V99, (box 30 × 30 × 30) and to the rat 15-LOX, 1LOX, (box 35 × 35 × 35), led to adequate amino acid interaction patterns (Table 5, Figure 4, Figure 6, Figure 7 and Figure 8). These results can be used for the prediction of activity to the human 5-LOX and for specificity evaluation.

The use of the soybean LOX-3 structure, 1JNQ also led to adequate amino acid interaction patterns. Docking studies were performed using the Molegro virtual docker. The re-rank scores which indicated improved docking accuracy over the Moldock score correlated relatively well with the observed LOX inhibitory activity (pIC_50_).

## 3. Materials and Methods

### 3.1. General Information

Solvents, unless otherwise specified, were of analytical reagent grade or of the highest quality commercially available. Synthetic starting materials, reagents and solvents were purchased from InterBioscreen (Chernogolovka, Russia, https://www.ibscreen.com/) and mycophenolic acid (CAS number 24280-93-1) was from Sigma-Aldrich Chemie (Steinheimm, Germany). Melting points (°C) were determined with a Boetius apparatus and are reported without correction. ^1^H-NMR spectra of the novel synthesized compounds in DMSO-*d*_6_ solutions were recorded on an AC 300 instrument (Bruker, (Bruker, Karlsruhe, Germany) at 298 K. ^13^C-NMR in d-CHCl_3_ were recorded on Bruker AC 500 instrument. Chemical shift (δ) values for ^1^H-NMR spectra are reported in parts per million (ppm) with the solvent resonance as the internal standard. MS spectra were recorded on an ESI-MS instrument (Micromass ZMD Waters, Milford, MA, USA). TLC analyses were performed with Merck silica gel 60 F254 precoated plates, and each of the synthesized compounds showed a single spot. High-resolution mass spectra (HRMS) were registered on Bruker MicrOTOF ESI-TOF mass spectrometer (see Supplementary Material).

### 3.2. Biological Activity Spectra Prediction

Prediction of activity spectra (PASS) is software training set of which consist of 950,801 drugs, drug candidates and toxic substances. It simultaneously predicts 7158 kinds of biological activities, mechanism of action, toxicity etc. The average accuracy of prediction in leave-one-out cross validation (LOO CV) for the whole training set is 95%. Details of the method used for prediction are described in the website [71] and in our paper [27].

### 3.3. Docking Analysis

Docking analysis to soybean LOX-1 (1YGE) and to human 5-LOX structures 3O8Y and 3V99 were carried out using Molecular Docking Server [72] as previously described in our paper [56]. In all cases, during enzyme preparation for docking analysis, the bound ligand was extracted but the Fe atom was maintained in the structure, a practice used in conjugated proteins or metaloenzymes/metalloproteins such as LOX. The pH for ligand preparation was set at 7.0 in all cases.

Docking analysis to soybean 3-LOX was performed using the Molegro virtual docker 4.0, and Discovery studio visualizer 3.0 (DS3.0). MolDock, a docking module of Molegro Virtual Docker (MVD) software [68], is based on a new hybrid search algorithm, called guided differential evolution (DE) which combines the DE optimization techniques with a cavity prediction algorithm, as described in previous paper [73]. This algorithm enables a fast and accurate identification of potential binding modes (poses). Moreover, a re-ranking process was applied to the highest ranked poses to further increase docking accuracy. The epigallocatechin from 1JNQ was used as the template with the default settings for docking studies, in a grid size of 15 Å radius defining the binding site with a grid resolution of 0.30, as previously reported by our group [74].

For the validation of the docking protocol, the reference compound epigallocatechin was docked in the binding site of LOX protein (PDB ID 1JNQ). Similarity of the docking interactions with important residues of the binding site between the tested compounds and co-crystalized ligand was observed (Figure 5A).

### 3.4. Chemistry

The initial compounds were purchased from the InterBioscreen Ltd. (Chernogolovka, Russia) chemical library, consisting of over 600,000 unique HTS-compounds including ~60,000 natural products, their derivatives and mimetics. Compound **1** was mentioned in the literature [75].

#### General Method for the Synthesis of Mycophenolic Acid Derivatives

To a solution of mycophenolic acid (2 mmol) in absolute DMF (1.5 mL) carbonyldiimidazole (2.2 mmol) was added and stirred for 2 h at room temperature. Then appropriate amine (2.2 mmol) was added and the reaction mixture is stirred at 70 °C for 10 h. The mixture was cooled to room temperature and distilled water (10 mL) was added. The precipitate formed was filtered and crystallized from aqueous methanol.

(E)-6-(4-Hydroxy-6-methoxy-7-methyl-3-oxo-1,3-dihydroisobenzofuran-5-yl)-4-methyl-N-(thiazol-2-yl)hex-4-enamide (**1**) [75]. Yield 62%, m.p. 216–217 °C. ^1^H-NMR (300 MHz, DMSO-d_6_) δ: 1.77 (s, 3H, CH_3_), 2.05(s, 3H, CH_3__(arom)_), 2.26 (t, 2H, J = 7.0 Hz, CH_2_CH_2_), 2.48 (t, 2H, J = 7.0 Hz, CH_2_CH_2_), 3.29 (d, 2H, J = 6.1 Hz, CH_2_CH), 3.64 (s, 3H, OCH_3_), 5.16 (t, 1H, J = 6.1 Hz, CH_2_CH), 5.23 (s, 2H, OCH_2_), 7.15 (s, 1H_(thiaz)_), 7.42 (s, 1H_(thiaz)_), 9.32 (bs, 1H, OH), 11.97 (bs, 1H, NH). ^13^C-NMR (500 MHz, CHCl_3_-d_6_): 11.50, 16.46, 23.42, 34.32, 34.76, 60.97, 68.31, 112.40, 113.39, 119.94, 123.89, 128.70, 133.54, 136.06, 146.69, 156.66, 159.59, 162.63, 168.80, 170.59. EI MS (m/z): 402 (M^+^, 16), 261 (14), 207 (22), 195 (43), 159 (22), 142 (100), 127 (21), 100 (69), 91 (11), 55 (13). HRMS (ESI), m/z found 403.1322 [M + H] C_20_H_23_N_2_O_5_S. Calculated: [M + H] 403.1328.

(E)-6-(4-Hydroxy-6-methoxy-7-methyl-3-oxo-1,3-dihydroisobenzofuran-5-yl)-4-methyl-N-(5-methylthiazol-2-yl)hex-4-enamide (**2**). Yield 65%, m.p. 215–216 °C. ^1^H-NMR (300 MHz, DMSO-d_6_) δ: 1.76 (s, 3H, CH_3_), 2.03(s, 3H, CH_3(arom)_), 2.25 (t, 2H, J = 7.5 Hz, CH_2_CH_2_), 2.31(s, 3H, CH_3(thiaz)_), 2.46 (t, 2H, J = 7.5 Hz, CH_2_CH_2_), 3.30 (d, 2H, J = 6.8 Hz, CH_2_CH), 3.66 (s, 3H, OCH_3_), 5.16 (t, 1H, J = 6.7 Hz, CH_2_CH), 5.22 (s, 2H, OCH_2_), 7.06 (s, 1H_(thiaz)_), 9.14 (bs, 1H, OH), 11.64 (bs, 1H, NH). ^13^C-NMR (500 MHz, CHCl_3_-d_6_): 11.48, 16.22, 22.94, 34.21, 34.30, 61.07, 69.54, 106.83, 116.42, 122.26, 124.59, 124.63, 126.99, 133.34, 133.69, 144.29, 153.72, 157.59, 163.17, 170.47, 172.15. EI MS (m/z): 416 (M^+^, 40), 209 (41), 195 (9), 169 (7), 156 (66), 141 (24), 114 (100), 81 (7), 69 (10), 44 (9). HRMS (ESI), m/z found 417.1479 [M + H] C_21_H_25_N_2_O_5_S. Calculated: [M + H] 417.1484.

(E)-N-(4,5-Dimethylthiazol-2-yl)-6-(4-hydroxy-6-methoxy-7-methyl-3-oxo-1,3-dihydroisobenzofuran-5-yl)-4-methylhex-4-enamide (**3**). Yield 72%, m.p. 202–203 °C. ^1^H-NMR (500 MHz, DMSO-d_6_) δ: 1.79 (s, 3H, CH_3_), 2.05 (s, 3H_(arom)_), 2.12 (s, 3H, CH_3(thiaz)_), 2.20 (s, 3H, CH_3(thiaz)_), 2.22 (t, 2H, J = 7.5 Hz, CH_2_CH_2_), 2.52 (m, 2H, CH_2_CH_2_), 3.28 (d, 2H, J = 6.6 Hz, CH_2_CH), 3.65 (s, 3H, OCH_3_),5.13 (t, 1H, J = 6.6 Hz, CH_2_CH), 5.22 (s, 2H, OCH_2_), 9.37 (br, 1H, OH), 11.37 (br, 1H, NH).^13^C-NMR (500 MHz, DMSO-d_6_) ^13^C-NMR: 10.74, 14.26, 16.38, 22.72, 34.41,34.92, 61.02, 69.83, 106.42, 116.59, 119.87, 122.02, 123.54, 133.70, 141.30, 144.08, 153, 58, 154.34, 163.45, 170.00, 172.56. EI MS (m/z): 430 (M^+^, 8), 223 (21), 170 (42), 155 (20), 141 (13), 128 (100), 114 (18), 95 (17), 85 (16), 59 (10). HRMS (ESI), m/z found 431.1635 [M + H]. C_22_H_27_N_2_O_5_S. Calculated: [M + H] 431.1640.

(E)-6-(4-Hydroxy-6-methoxy-7-methyl-3-oxo-1,3-dihydroisobenzofuran-5-yl)-4-methyl-N-(4-methylthiazol-2-yl)hex-4-enamide (**4**). Yield 68%, m.p. 185–186 °C. ^1^H-NMR (300 MHz, DMSO-d_6_) δ: 1.74 (s, 3H, CH_3_), 2.03 (s, 3H, CH_3(arom)_), 2.21 (s, 3H, CH_3(thiaz)_), 2.24 (m, 2H, CH_2_CH_2_), 2.44 (t, 2H, J = 7.8 Hz, CH_2_CH_2_), 3.26 (d, 2H, J = 6.6 Hz, CH_2_CH), 3.63 (s, 3H, OCH_3_), 5.13 (t, 1H, J = 6.0 Hz, CH_2_CH), 5.21 (s, 2H, OCH_2_), 6.66 (s, 1H_(thiaz)_), 9.32 (bs, 1H, OH) 11.88 (bs, 1H, NH). ^13^C-NMR (500 MHz, CHCl_3_-d_6_): 11.50, 16.40, 22.73, 34.35, 34.94, 51.09, 60.96, 69.85, 106.42, 108.00, 116.60, 121.96, 123.62, 133.66, 144.09, 146.77, 153.55, 157.60, 162.44, 170.19, 172.57. EI MS (m/z): 416 (M^+^, 19), 209 (29), 195 (7), 156 (60), 141 (23), 124 (11), 114 (100), 69 (8), 55 (7), 41 (6). HRMS (ESI), m/z found: 417.1479 [M + H] C_21_H_25_N_2_O_5_S. Calculated: [M + H] 417.1484.

(E)-Ethyl-2-(6-(4-hydroxy-6-methoxy-7-methyl-3-oxo-1,3-dihydroisobenzofuran-5-yl)-4-methylhex-4-enamido)-4-methylthiazole-5-carboxylate (**5**). Yield 63%, m.p. 189–190 °C. ^1^H-NMR (300 MHz, DMSO-d_6_) δ: 1.26 (t, 3H, J = 7.1 Hz, CH_2_CH_3_), 1.74 (s, 3H, CH_3_), 1.99 (s, 3H, CH_3(arom)_), 2.24 (t, 2H, J = 7.1 Hz, CH_2_CH_2_), 2.45 (m, 5H, CH_3(thiaz),_ CH_2_CH_2_), 3.24 (d, 2H, J = 6.6 Hz, CH_2_CH), 3.62 (s, 3H, OCH_3_), 4.21 (q, 2H, J = 7.1 Hz, CH_2_CH_3_), 5.12 (m, 1H, CH_2_CH), 5.14 (s, 2H, OCH_2_), 9.28 (bs, 1H, OH), 12.32 (bs, 1H, NH). ^13^C-NMR (500 MHz, DMSO-d_6_) δ: 11.49, 14.32, 16.42, 16.98, 22.76, 34.28, 34.96, 60.85, 61.04, 62.41, 69.89, 116.61, 120.31, 121.78, 123.90, 133.37, 144.12, 148.44, 153.48, 156.14, 159.21, 170.55, 178.25. EI MS (m/z): 488 (M^+^, 9), 281 (33), 228 (51), 207 (24), 186 (100), 159 (44), 141 (29), 114 (22), 91 (12), 71 (10). HRMS (ESI), m/z found: 489.1683 [M + H] C_24_H_28_N_2_O_7_S. Calculated: [M + H] 489.1695.

(E)-Ethyl-2-(2-(6-(4-hydroxy-6-methoxy-7-methyl-3-oxo-1,3-dihydroisobenzofuran-5-yl)-4-methylhex-4-enamido)thiazol-4-yl)acetate (**6**). Yield 59%, m.p. 233–234 °C. ^1^H-NMR (500 MHz, CHCl_3_-d_6_) δ: 1.16 (t, 3H, J = 7.0 Hz, CH_2_CH_3_), 1.74 (s, 3H, CH_3_), 2.03 (s, 3H, CH_3(arom)_), 2.23 (t, 2H, J = 7.1 Hz, CH_2_CH_2_), 2.45 (t, 2H, J = 7.0 Hz), CH_2_CH_2_), 3.26 (d, 2H, J = 6.0 Hz, CH_2_CH), 3.61 (s, 3H, OCH_3_), 3.64 (s, 2H, CH_2_CO), 4.06 (q, 2H, J = 7.0 Hz, CH_2_CH_3_), 5.12 (m, 1H, CH_2_CH), 5.21 (s, 2H, OCH_2_), 6.90 (s, 1H_(thiaz)_), 9.31 (s, 1H, OH), 12.03 (s, 1H, NH). ^13^C-NMR (500 MHz, DMSO-d_6_): 11.49, 14.32, 16.42, 16.98, 22.76, 34.28, 34.96, 60.85, 61.04, 62.41, 69.89, 116.61, 120.31, 121.78, 123.90, 133.37, 140.11, 144.12, 148.44, 153.48, 156.14, 159.21, 170.55, 178.25. EI MS (m/z): 488 (M^+^, 6), 281 (28), 228 (46), 207 (33), 186 (100), 159 (24), 141 (11), 128 (11), 113 (78), 91 (22), 71 (14). HRMS (ESI), m/z found: 489.1690 [M + H]. C_24_H_28_N_2_O_7_S. Calculated: [M + H] 489.1695.

(E)-6-(4-Hydroxy-6-methoxy-7-methyl-3-oxo-1,3-dihydroisobenzofuran-5-yl)-4-methyl-N-(4-(pyridin-4-yl)thiazol-2-yl)hex-4-enamide (**7**). Yield 56%, m.p. 193–194 °C. ^1^H-NMR (300 MHz, DMSO-d_6_) δ: 1.76 (s, 3H, CH_3_), 1.98 (s, 3H, CH_3(arom)_), 2.26 (t, 2H, J = 7.5 Hz, CH_2_CH_2_), 2.52 (t, 2H, J = 7.5 Hz, CH_2_CH_2_), 3.26 (d, 2H, J = 6.6 Hz, CH_2_CH), 3.62 (s, 3H, O CH_3_), 5.15 (t, 1H, J = 6.6 Hz, CH_2_CH), 5.16 (s, 2H, OCH_2_), 7.80 (d, 2H, J = 4.8 Hz, H_(py)_), 7.91 (s, 1H, H_(thiaz)_), 8.60 (d, 2H, J = 4.8 Hz, H_(py)_), 9.31 (s, 1H, OH), 12.22 (1H, NH). ^13^C-NMR (500 MHz, DMSO-d_6_) 11.50, 16.40, 16.55, 19.95, 22.73, 34.55, 34.94, 60.96, 66.80, 69.02, 106.42, 108.00, 115.60, 116.60, 120.78, 121.96, 123.62, 144.09, 146.77, 153.55, 157.68, 163.44, 170.19, 172.97. EI MS (m/z): 479 (M^+^, 8), 272 (16), 219 (35), 207 (15), 177 (100), 159 (32), 135 (37), 105 (34), 91 (33), 77 (28), 55 (15). HRMS (ESI), m/z found: 480.1588 [M + H] C_25_H_26_N_3_O_5_S. Calculated: [M + H] 480.1593.

E)-6-(4-Hydroxy-6-methoxy-7-methyl-3-oxo-1,3-dihydroisobenzofuran-5-yl)-4-methyl-N-(4-(pyridin-3-yl)thiazol-2-yl)hex-4-enamide (**8**). Yield 61%, m.p. 182–183 °C. ^1^H-NMR (300 MHz, DMSO-d_6_) δ: 1.77 (s, 3H, CH_3_), 2.00 (s, 3H, CH_3(arom)_), 2.26 (t, 2H, J = 6.8 Hz, CH_2_CH_2_), 2.53 (t, 2H, J = 6.8 Hz, CH_2_CH_2_), 3.26 (d, 2H, J = 6.5 Hz, CH_2_CH), 3.64 (s, 3H, OCH_3_), 5.15 (t, 1H, J = 6.5 Hz, CH_2_CH), 5.17 (s, 2H, OCH_2_), 7.45 (dd, 1H, J = 4.8, 7.6 Hz, H_(py)_), 7.74 (s, 1H, H_(thiaz)_), 8.19 (d, 1H, J = 7.6 Hz, H_(py)_), 8.50 (d, 1H, J = 4.8 Hz, H_(py)_), 9.08 (s, 1H, OH), 9.34 (s, 1H, H_(py)_), 12.21 (s, 1H, NH). ^13^C-NMR (500 MHz, DMSO-d_6_): 11.50, 16.40, 22.73, 34.33, 34.94, 60.94, 69.70, 106.42, 108.00, 116.60, 121.96, 123.62, 125.04, 133.34, 133.66, 134.55, 144.09, 146.77, 147.75, 148.56, 153.55, 157.60, 163.44, 170.19, 172.57. EI MS (m/z): 479 (M^+^, 9), 272 (12), 219 (27), 204 (9), 177 (100), 159 (23), 135 (37), 105 (23), 91 (23), 77 (16), 55, (14). HRMS (ESI), m/z found: 480.1588 [M + H] C_25_H_26_N_3_O_5_S. Calculated: [M + H] 480.1593.

(E)-6-(4-Hydroxy-6-methoxy-7-methyl-3-oxo-1,3-dihydroisobenzofuran-5-yl)-4-methyl-N-(4-(pyridin-2-yl)thiazol-2-yl)hex-4-enamide (**9**). Yield 58%, m.p. 217–218 °C. ^1^H-NMR (300 MHz, CHCl_3_-d_6_) δ: 1.77 (s, 3H, CH_3_), 2.02 (s, 3H, CH_3(arom)_), 2.30 (t, 2H, J = 7.2 Hz, CH_2_CH_2_), 2.52 (t, 2H, J = 7.2 Hz, CH_2_CH_2_), 3.29 (d, 2H, J = 6.7 Hz, CH_2_CH), 3.67 (s, 3H, OCH_3_), 5.13 (s, 2H, OCH_2_), 5.21(t, 1H, J = 6.7 Hz, CH_2_CH), 7.24 (m, 1H, H_(py)_), 7.63 (s, 1H, H_(thiaz)_), 7.79 (m, 1H, H_(py)_), 7.89 (d, 1H, J = 7.9 Hz, H_(py)_), 8.54 (d, 1H, J = 4.6 Hz, H_(py)_), 8.78 (s, 1H, OH), 11.96 (s, 1H, NH).^.^^13^C- NMR (500 MHz, DMSO-d_6_): 11.50, 16.40, 22.73, 34.33, 34.94, 60.94, 69.70, 106.42, 108.00, 116.60, 121.96, 123.62, 123.62, 127.00, 128.91, 133.66, 144.09, 146.77, 149.82, 150.50, 150.96, 153.55, 157.55, 163.44, 170.19, 172.57. EI MS (m/z): 479 (M^+^, 19), 272 (11), 219 (41), 177 (100), 159 (18), 135 (69), 105 (48), 91 (48), 78 (72), 67 (38), 55 (30). HRMS (ESI), m/z found: 480.1588 [M + H] C_25_H_26_N_3_O_5_S. Calculated: [M + H] 480.1593.

(E)-N-(Benzo[d]thiazol-2-yl)-6-(4-hydroxy-6-methoxy-7-methyl-3-oxo-1,3-dihydroisobenzofuran-5-yl)-4-methylhex-4-enamide (**10**). Yield 60%, m.p. 201–202 °C. ^1^H-NMR (300 MHz, DMSO-d_6_) δ: 1.77 (s, 3H, CH_3_), 1.98 (s, 3H, CH_3(arom)_), 2.30 (t, 2H, J = 7.5 Hz, CH_2_CH_2_), 2.56 (t, 2H, J = 7.5 Hz, CH_2_CH_2_), 3.28 (d, 2H, J = 6.6 Hz, CH_2_CH), 3.65 (s, 3H, OCH_3_), 5.06 (s, 2H, OCH_2_), 5.20 (t, 1H J = 6.6 Hz, CH_2_CH), 7.29 (t, 1H, J = 7.5 Hz, H_(arom)_), 7.42 (t, 1H, J = 7.5 Hz, H_(arom)_), 7.68 (d, 1H, J = 8.0 Hz, H_(arom)_), 7.91 (d, 1H, J = 8.0 Hz, H_(arom)_), 9.10 (bs, 1H, OH), 12.11 (bs, 1H, NH). ^13^C-NMR (500 MHz, DMSO-d_6_): 11.45, 16.22, 22.94, 26.82, 34.21, 34.30, 61.07, 69.54, 106.65, 116.42, 120.71, 122.26, 124.59, 124.63, 126.99, 133.34, 133.69, 144.29, 153.72, 127.59, 163.17, 170.47, 172.15, 172.76. EI MS (m/z): 452 (M^+^, 25), 45 (40), 207 (14), 192 (49), 177 (20), 163 (6), 150 (100), 135 (10), 73 (6), 45 (14). HRMS (ESI), m/z found: 453.1479 [M + H] C_24_H_25_N_2_O_5_S. Calculated: [M + H] 453.1484.

(E)-6-(4-Hydroxy-6-methoxy-7-methyl-3-oxo-1,3-dihydroisobenzofuran-5-yl)-N-(6-methoxybenzo[d]thiazol-2-yl)-4-methylhex-4-enamide (**11**). Yield 64%, m.p. 205–206 °C. ^1^H-NMR (300 MHz, DMSO-d_6_) δ: 1.78 (s, 3H, CH_3_), 1.98 (s, 3H, CH_3(arom)_), 2.28 (t, 2H, J = 7.0 Hz, CH_2_CH_2_), 2.51 (t, 2H, J = 7.0 Hz, CH_2_CH_2_), 3.28 (d, 2H, J = 6.1 Hz, CH_2_CH), 3.65 (s, 3H, OCH_3_), 3.81 (s, 3H, OCH_3_) 5.08 (s, 2H, OCH_2_), 5.17 (t, 1H J = 6.1 Hz, CH_2_CH), 7.01 (d, 1H, J = 8.2 Hz, H_(arom)_), 7.52 (s, 1H, H_(arom)_), 7.59 (d, 1H, J = 8.2 Hz, H_(arom)_), 9.31 (bs, 1H, OH), 12.08 (bs, 1H, NH). ^13^C-NMR (500 MHz, DMSO-d_6_): 11.50, 16.40, 16.92, 19.95, 22.73, 34.35, 34.94, 60.96, 66.80, 69.85, 106.42, 108.00, 115.60, 116.60, 120.78, 121.96, 123.62, 133.66, 144.09, 146.77, 153.55, 157.68, 163.44, 170.194, 172.57. EI MS (m/z): 482 (M^+^, 13), 281 (11), 222 (25), 207 (40), 193 (25), 180 (100), 165 (77), 160 (52), 135 (54), 115 (34), 103 (24), 91 (52), 77 (37). HRMS (ESI), m/z found: 483.1584 [M + H] C_25_H_27_N_2_O_6_S. Calculated: [M + H] 483.1589

(E)-6-(4-Hydroxy-6-methoxy-7-methyl-3-oxo-1,3-dihydroisobenzofuran-5-yl)-4-methyl-N-(4,5,6,7-tetrahydrobenzo[d]thiazol-2-yl)hex-4-enamide (**12**). Yield 69%, m.p. 179–180 °C. ^1^H-NMR (300 MHz, DMSO-d_6_) δ: 1.73 (m, 7H, CH_3,_ CH_2_CH_2_CH_2_CH_2_), 2.04 (s, 3H, CH_3(arom)_), 2.21 (m, 2H, CH_2_CH_2_), 2.42 (m, 2H, CH_2_CH_2_), 2.59 (m, 4H, CH_2_CH_2_CH_2_CH_2_), 3.27 (d, 2H, J = 6.6 Hz, CH_2_CH), 3.64 (s, 3H, OCH_3_), 5.12 (m, 1H, CH_2_CH), 5.21 (s, 2H, OCH_2_), 9.37(bs, 1H, OH), 11.76 (bs, 1H, NH). ^13^C-NMR (500 MHz, DMSO-d_6_): 11.48, 16.22, 22.94, 22.94, 24.21, 24.30, 34.21, 34.30, 61.07, 69.54, 106.83, 116.42, 122.26, 124.59., 124.63, 126.99, 133.34, 133.69, 144.29, 153.72, 157.59, 163.17, 170.47, 172.15. EI MS (m/z): 456 (M^+^, 7), 249 (24), 207 (9), 196 (32), 181 (16), 154 (100), 126 (58), 11 (11), 91 (14), 77 (18), 67 (24). HRMS (ESI), m/z found: 457.1792 [M + H] C_24_H_29_N_2_O_5_S. Calculated: [M + H] 457.1797.

(E)-6-(4-Hydroxy-6-methoxy-7-methyl-3-oxo-1,3-dihydroisobenzofuran-5-yl)-4-methyl-N-(5,6,7,8-tetrahydro-4H-cyclohepta[d]thiazol-2-yl)hex-4-enamide (**13**). Yield 67%, m.p. 198–199 °C. ^1^H-NMR (300 MHz, DMSO) δ: 1.62 (m, 4H, 2x CH_2_), 1.75 (s, 3H, CH_3_), 1.78 (m, 2H, CH_2_), 2.07 (s, 3H, CH_3(arom)_), 2.24 (t, 2H, J = 7.6 Hz, CH_2_CH_2_), 2.43 (t, 2H, J = 7.6 Hz, CH_2_CH_2_), 2.65 (t, 2H, J = 5.4 Hz, CH_2_), 2.72 (t, 2H, t, 2H, J = 5.4 Hz, CH_2_), 3.29 (d, 2H, J = 6.6 Hz, CH_2_CH), 3.66 (s, 3H, OCH_3_), 5.15 (t, 1H, J = 6.6 Hz, CH_2_CH), 5.22 (s, 2H, OCH_2_), 9.13 (bs, 1H, OH), 11.57 (bs, 1H, NH). ^13^C-NMR (500 MHz, DMSO-d_6_): 11.560, 16.40, 16.92, 19.95, 22.73, 25.19, 34.35, 60.96, 66.00, 69.05, 106.42, 108.00, 115.60, 116.60, 121.96, 123.62, 133.66, 144.09, 146.77, 146.77, 153.55, 157.69, 163.44, 170.19, 172.57, 173.75. EI MS (m/z): 470 (M^+^, 10), 263 (36), 210 (35), 195 (24), 168 (100), 153 (8), 139 (12), 126 (14), 114 (16), 91 (13), 81 (11), 55 (9). HRMS (ESI), m/z found: 471.1948 [M + H] C_25_H_31_N_2_O_5_S. Calculated: [M + H] 471.1953.

(E)-N-(5,6-Dihydro-4H-cyclopenta[d]thiazol-2-yl)-6-(4-hydroxy-6-methoxy-7-methyl-3-oxo-1,3-dihydroisobenzofuran-5-yl)-4-methylhex-4-enamide (**14**). Yield 62%, m.p. 166–167 °C. ^1^H-NMR (300 MHz, DMSO-d_6_) δ: 1.76 (s, 3H, CH_3_), 2.06 (s, 3H, CH_3(arom)_), 2.25 (t, 2H, J = 7.4 Hz, CH_2_CH_2_), 2.37 (m, 2H, CH_2_CH_2_CH_2_), 2.46 (m, 2H, CH_2_CH_2_), 2.64 (t, 2H, J = 7.0 Hz, CH_2_CH_2_CH_2_), 2.79 (t, 2H, J = 7.0 Hz, CH_2_CH_2_CH_2_), 3.30 (d, 2H, J = 6.6 Hz, CH_2_CH), 3.67 (s, 3H, OCH_3_), 5.16 (t, 1H, J = 6.6 Hz, CH_2_CH), 5.22 (s, 2H, OCH_2_), 9.17 (bs 1H, OH), 11.68 (bs, 1H, NH). ^13^C-NMR (500 MHz, DMSO-d_6_) δ: 11.51, 16.38, 22.72, 25.72, 25.83, 31.92, 34.41, 34.92, 61.02, 69.83, 106.42, 116.59, 119.87, 122.02, 122.54, 133.78, 141.30, 144.08, 153.58, 154.34, 163.45, 170.00, 172.56. EI MS (m/z): 442 (M^+^, 10), 182 (11), 159 (15), 140 (100), 128 (12), 115 (15), 97 (18), 81 (45), 67 (14), 53 (15), 41 (11). HRMS (ESI), m/z found: 443.1635 [M + H] C_23_H_27_N_2_O_5_S. Calculated: [M + H] 443.1640.

(E)-6-(4,6-Dimethoxy-7-methyl-3-oxo-1,3-dihydroisobenzofuran-5-yl)-4-methyl-N-(thiazol-2-yl)hex-4-enamide (**15**). Yield 74%, m.p. 219–220 °C. ^1^H-NMR (300 MHz, DMSO) δ: 1.77 (s, 3H, CH_3_), 2.09 (s, 3H, CH_3(arom)_), 2.26 (t, 2H, J = 7.2 Hz, CH_2_CH_2_), 2.52 (m, 2H, CH_2_CH_2_), 3.28 (d, 2H, J = 6.5 Hz, CH_2_CH), 3.67 (s, 3H, OCH_3_), 3.87 (s, 3H, OCH_3_), 5.09 (t, 1H, J = 6.5 Hz, CH_2_CH), 5.24 (s, 2H, OCH_2_), 7.12 (d, 1H, J = 3.6 Hz, H_(thiaz)_), 7.40 (d, 1H, J = 3.6 Hz, H_(thiaz)_), 11.97 (s, 1H, NH). ^13^C-NMR (500 MHz, DMSO-d_6_), δ: 11.50, 16.46, 23.42, 34.32, 34.76, 60.97, 62.58, 68.31, 112.40, 113.39, 119.94, 123.89, 128.70, 133.54, 136.06, 146.69, 156.66, 159.59, 162.63, 168.80, 170.59. EI MS (m/z): 416 (M^+^, 5), 221 (24), 209 (15), 195 (55), 182 (14), 142 (100), 127 (16), 115 (12), 100 (56), 69 (13), 55 (16). HRMS (ESI), m/z found 417.1479 [M + H] C_21_H_25_N_2_O_5_S. Calculated: [M + H] 417.1484.

(E)-6-(4,6-Dimethoxy-7-methyl-3-oxo-1,3-dihydroisobenzofuran-5-yl)-4-methyl-N-(5-methylthiazol-2-yl)hex-4-enamide (**16**). Yield 74%, m.p. 185–186 °C. ^1^H-NMR (300 MHz, DMSO) δ: 1.75 (s, 3H, CH_3_), 2.09 (s, 3H, CH_3(arom)_), 2.24 (t, 2H, J = 6.8 Hz, CH_2_CH_2_), 2.28 (s, 3H, CH_3(thiaz)_), 2.45 (t, 2H, J = 6.8 Hz, CH_2_CH_2_), 3.28 (d, 2H, J = 6.6 Hz, CH_2_CH), 3.68 (s, 3H, OCH_3_), 3.87 (s, 3H, OCH_3_), 5.08 (t, 1H, J = 6.6 Hz, CH_2_CH), 5.23 (s, 2H, OCH_2_), 7.05 (s, 1H, H_(thiaz)_), 11.76 (bs, 1H, NH). ^13^C-NMR (500 MHz, DMSO-d_6_), δ: 11.48, 16.41, 22.42, 34.45, 34.72, 46.70, 60.94, 62.58, 69.29, 112.47, 119.91, 123.95, 127.23, 128.73, 132.75, 133.56, 133.42, 141.30, 146.68, 156.66, 168.85, 170.32. EI MS (m/z): 430 (M^+^, 9), 221 (24), 209 (100), 196 (22), 175 (10), 156 (57), 141 (18), 127 (13), 114 (60), 91 (10). HRMS (ESI), m/z found 431.1635 [M + H] C_22_H_27_N_2_O_5_S. Calculated: [M + H] 431.1640.

### 3.5. Biological Assay

#### Soybean Lipoxygenase Inhibition Study In Vitro

For the evaluation of lipoxygenase inhibition soybean LOX type 1b was used as reported previously [26,27]. The selection was based on the structural and functional similarity of sLOX-1 with mammalian LOX [36,50,51,52] which makes it the most commonly used LOX enzyme in drug screening, followed by sLOX-3 [40]. Interestingly, a greater identity is observed between human 5-LOX and soybean LOX b than with potato 5-LOX which has also been used in LOX inhibition assays, recently.

The assay conditions were as previously reported [27,64]. Different compound concentrations were used for the calculation of IC_50_ values. The experiments were performed in triplicate.

## 4. Conclusions

All compounds exhibited inhibitory action with IC_50_ values ranging from 2.5 μm to 150 μΜ depending on the structure of the compounds. Hydrogen bond formation and pi-pi interactions are involved in complex stabilization of the most potent compounds according to docking studies. The presence of an electronegative atom close to position 4 of the thiazolyl moiety seams to enhance the activity of the compounds of this series, probably due to participation of hydrogen bond formation.

Docking studies using two different human 5-LOX structure (3O8Y and 3V99) one mammalian 15-LOX structure (1LOX) and two soybean structures (sLOX-1: 1YGE and LOX-3: 1JNQ) showed that the structures of enzymes crystallized without substrate or inhibitor (1YGE, 3O8Y) cannot be used for prediction of activity of this series of compounds. On the other hand, docking to soybean 1JNQ using the MolDock software adequately estimated inhibitory action and amino acid interactions. Moreover, the human 5-LOX structure 3V99 and the mammalian 15-LOX structure 1LOX can be used for estimation of human 5-LOX and 15-LOX inhibition, selectivity and amino acid interactions.

## Figures and Tables

**Table 1:**
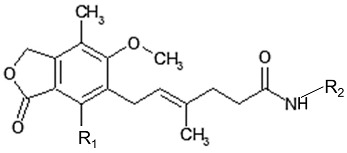
Structure of the designed compounds.

Comp.	R_1_	R_2_	Comp.	R_1_	R_2_
1	-OH	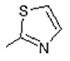	9	-OH	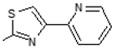
2	-OH	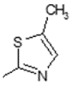	10	-OH	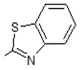
3	-OH	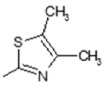	11	-OH	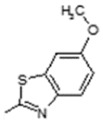
4	-OH	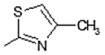	12	-OH	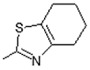
5	-OH	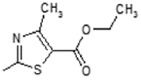	13	-OH	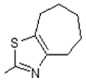
6	-OH	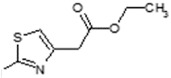	14	-OH	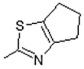
7	-OH	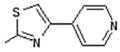	15	-OCH_3_	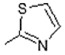
8	-OH	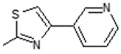	16	-OCH_3_	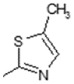

**Table 2:**
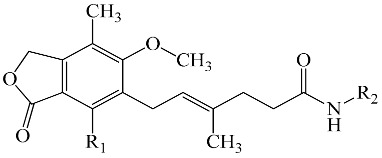
Prediction of the biological activity spectra of the designed compounds.

No.	R_2_	Predicted Activity	Pa
1	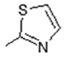	Systemic lupus erythematosus treatment	0.905
		Lipoxygenase inhibitor	0.846
		Transcription factor inhibitor	0.768
		Immunosuppressant	0.727
		Lipid peroxidase inhibitor	0.682
		Antiulcerative	0.674
2	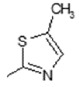	Lipoxygenase inhibitor	0.879
		Systemic lupus erythematosus treatment	0.72
		Immunosuppressant	0.726
		Antineoplastic	0.696
		Antiinflammatory	0.645
		Lipid peroxidase inhibitor…	0.6
3	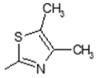	Antiulcerative	0.748
		Lipoxygenase inhibitor	0.743
		Immunosuppressant	0.72
		Systemic lupus erythematosus treatment	0.662
		Antiinflammatory	0.632
		Lipid peroxidase inhibitor	0.601
		Antineoplastic…	0.639
4	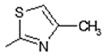	Lipoxygenase inhibitor	0.808
		Antiulcerative	0.794
		Transcription factor inhibitor	0.76
		Systemic lupus erythematosus treatment	0.734
		Immunosuppressant	0.706
		Lipid peroxidase inhibitor	0.696
5	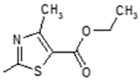	Immunosuppressant	0.745
		Lipoxygenase inhibitor	0.716
		Antiulcerative	0.689
		Systemic lupus erythematosus treatment	0.631
		Antiinflammatory	0.616
		Transcription factor inhibitor	0.594
		Antineoplastic…	0.581
6	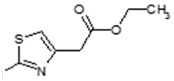	Lipoxygenase inhibitor	0.875
		Antiulcerative	0.799
		Transcription factor inhibitor	0.753
		Immunosuppressant	0.731
		Antiinflammatory	0.692
		Systemic lupus erythematosus treatment	0.587
		Antineoplastic	0.609
7	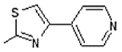	Antiulcerative	0.765
		Lipoxygenase inhibitor	0.726
		Transcription factor inhibitor	0.726
		Immunosuppressant	0.716
		Systemic lupus erythematosus treatment	0.699
		Mediator release inhibitor	0.642
8	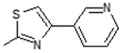	Transcription factor inhibitor	0.751
		Antiulcerative	0.709
		Immunosuppressant	0.7
		Systemic lupus erythematosus treatment	0.689
		Mediator release inhibitor	0.662
		Lipoxygenase inhibitor	0.661
9	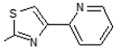	Antiulcerative	0.825
		Lipoxygenase inhibitor	0.716
		Transcription factor inhibitor	0.7
		Immunosuppressant	0.688
		Systemic lupus erythematosus treatment	0.615
		Mediator release inhibitor	0.609
10	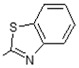	Lipoxygenase inhibitor	0.774
		Systemic lupus erythematosus treatment	0.746
		Lipid peroxidase inhibitor	0.722
		Immunosuppressant	0.7
		Antiulcerative	0.679
		Transcription factor inhibitor	0.673
11	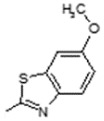	Lipoxygenase inhibitor	0.809
		Lipid peroxidase inhibitor	0.698
		Systemic lupus erythematosus treatment	0.688
		Immunosuppressant	0.687
		Antineoplastic	0.674
		Antiulcerative	0.63
		Apoptosis agonist...	0.615
12	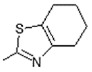	Antiulcerative	0.748
		Lipoxygenase inhibitor	0.743
		Immunosuppressant	0.72
		Systemic lupus erythematosus treatment	0.662
		Antiinflammatory	0.632
		Lipid peroxidase inhibitor	0.601
		Antineoplastic...	0.639
13	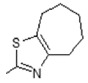	Antiulcerative	0.748
		Lipoxygenase inhibitor	0.743
		Immunosuppressant	0.72
		Systemic lupus erythematosus treatment	0.662
		Antiinflammatory	0.632
		Lipid peroxidase inhibitor	0.601
		Antineoplastic...	0.639
14	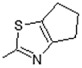	Antiulcerative	0.748
		Lipoxygenase inhibitor	0.743
		Immunosuppressant	0.72
		Systemic lupus erythematosus treatment	0.662
		Antiinflammatory	0.632
		Lipid peroxidase inhibitor	0,601
		Antineoplastic...	0.639
15	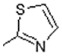	Systemic lupus erythematosus treatment	0.791
		Immunosuppressant	0.715
		Lipoxygenase inhibitor	0.697
		Transcription factor inhibitor	0.699
		Antiinflammatory	0.656
		Antineoplastic	0.664
		Antiulcerative	0.625
16	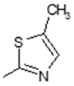	Lipoxygenase inhibitor	0.732
		Immunosuppressant	0.714
		Antineoplastic	0.692
		Antiinflammatory	0.625
		Systemic lupus erythematosus treatment	0.583
		Apoptosis agonist	0.576
		Antiulcerative	0.501

**Table 3:** Toxicity prediction by ToxPredict software.

No.	DDStox Carcinogenic Potency DBS MultiCellCall	DDStox Carcinogenic Potency DBS Mouse	DDStox Carcinogenic Potency DBS Rat	DDStox Carcinogenic Potency DBS SingleCellcall	DDStox Carcinogenic Potency DBS Hamster	DSSTox Carcinogenic Potency DBS Mutagenicity	Kazius-Bursi Salmonella Mutagenicity	FDA v3b Maximum Recommended Daily Dose mmol
	Non Carcinogen Confidence:	Non Carcinogen Confidence:	Non Carcinogen Confidence:	Non Carcinogen Confidence:	Non Carcinogen Confidence:	Non Mutagenic Confidence:	Non Mutagenic Confidence:	0.162 mmol Confidence:
1	0.084	0.132	0.082	0.0296	0.155	0.0345	0.0774	0.165
2	0.058	0.241	0.0541	0.0035	0.099	0.0801	0.0756	0.0735 mmol
3	0.139	0.252	0.0282	0.0108	0.084	0.0374	0.0791	0.0641 mmol
4	0.057	0.223	0.0342	0.0156	0.082	0.0567	0.0784	0.0724 mmol
5	0.120	0.253	0.0274	0.0245	0.096	0.0357	0.0698	0.188 mmol
6	0.086	0.142	0.0670	0.0276	0.081	0.0340	0.0768	0.0723 mmol
7	0.056	0.250	0.0567	0.0145	0.099	0.0786	0.0712	0.145 mmol
8	0.050	0.261	0.0022	0.0111	0.098	0.1051	0.0062	0.109 mmol
9	0.059	0.254	0.0459	0.0179	0.087	0.1131	0.0566	0.133 mmol
10	0.122	0.199	0.0677	0.0186	0.097	0.1291	0.0734	0.107 mmol
11	0.045	0.252	0.0141	0.0187	0.217	0.0146	0.0112	0.117 mmol
12	0.078	0.234	0.0781	0.0234	0.239	0.0358	0.0689	0.189 mmol
13	0.109	0.287	0.0678	0.0189	0.221	0.0347	0.0765	0.179 mmol
14	0.167	0.178	0.0443	0.0179	0.187	0.0156	0.0651	0.123 mmol
15	0.058	0.257	0.0448	0.0177	0.084	0.1132	0.0559	0.131 mmol
16	0.132	0.187	0.0657	0.0184	0.087	0.1275	0.0744	0.110 mmol
17	0.035	0.241	0.0124	0.0182	0.213	0.0191	0.0123	0.113 mmol

**Table 4:** Toxicity prediction by PTOTOX software.

Comp.	Predicted LD_50_	Predicted Toxicity Class	Hepatotoxicity	Carcinogenicity	Mutagenicity	Cytotoxicity
1	500 mg/kg	4	Inactive 0.64	Inactive 0.55	Inactive 0.63	Inactive 0.63
2	500 mg/kg	4	Inactive 0.64	Inactive 0.54	Inactive 0.63	Inactive 0.63
3	1000 mg/kg	4	Inactive 0.64	Inactive 0.55	Inactive 0.63	Inactive 0.63
4	1000 mg/kg	4	Inactive 0.64	Inactive 0.55	Inactive 0.63	Inactive 0.63
5	300 mg/kg	3	Inactive 0.64	Inactive 0.55	Inactive 0.63	Inactive 0.63
6	500 mg/kg	4	Inactive 0.64	Inactive 0.54	Inactive 0.63	Inactive 0.63
7	1000 mg/kg	4	Inactive 0.64	Inactive 0.55	Inactive 0.63	Inactive 0.63
8	1000 mg/kg	4	Inactive 0.64	Inactive 0.55	Inactive 0.63	Inactive 0.63
9	1000 mg/kg	4	Inactive 0.64	Inactive 0.55	Inactive 0.63	Inactive 0.63
10	500 mg/kg	4	Inactive 0.64	Inactive 0.54	Inactive 0.63	Inactive 0.63
11	1000 mg/kg	4	Inactive 0.64	Inactive 0.55	Inactive 0.63	Inactive 0.63
12	500 mg/kg	4	Inactive 0.64	Inactive 0.55	Inactive 0.63	Inactive 0.63
13	300 mg/kg	3	Inactive 0.64	Inactive 0.55	Inactive 0.63	Inactive 0.63
14	300 mg/kg	3	Inactive 0.64	Inactive 0.55	Inactive 0.63	Inactive 0.63
15	500 mg/kg	4	Inactive 0.64	Inactive 0.55	Inactive 0.63	Inactive 0.63
16	500 mg/kg	4	Inactive 0.64	Inactive 0.55	Inactive 0.63	Inactive 0.63

**Table 5:** Results of docking analysis to the rat 15-LOX, 1LOX, to human 5-LOX, 3V99 and the soybean LOX-3, 1JNQ, using different target center and box dimensions.

Comp.	Free Binding Energy (kcal/mole)			Docking Scores		IC_50_ (µM)	pIC_50_	Length
	Rat 15-LOX 1LOX		Human 5-LOX 3V99	1JNQ				
	Box 30	Box 35	Box 30	Moldock	Rerank			
1		−5.24		−117.175	−87.9702	26.25	−1.419	14.82
2		−6.68		−128.445	−72.0312	22.50	−1.352	15.72
3				−126.37	−75.89	43.75	−1.641	
4			−7.50	−128.45	−82.031	31.25	−1.495	16.29
5	−7.76	−6.43		−136.803	−83.9406	22.50	−3.343	18.79
6				−116.026	−57.1526	30.00	−1.477	15.23
7				−85.017	−61.202	78.75	−1.896	
8			−9.01	−119.09	−90.416	90.00	−1.954	18.22
9	−9.27	−8.29	−10.00	−142.373	−105.8901	2.50	−0.398	17.71
10				−61.93	−71.647	90.00	−1.954	
11	−9.25	−6.71	−7.49	−62.19	−59.631	156.25	−2.194	19.61
12						61.25	−1.787	
13				−142.186	−89.631	25.00	−1.398	16.90
14				−136.03	−77.1526	31.25	−1.498	
15			−6.27	−147.28	−47.554	96.25	−1.983	15.30
16				−152.14	−80.727	90.00	−1.954	
OPPA	−8.05	−8.09						
A.A			−6.46					

IC_50_ values are means of three determinations and deviation from the mean is <10% of the mean value. OPPA: 3-(2-oct-1-ynylphenyl) propanoic acid; A.A: Arachidonic acid.

**Table 6:** Results of docking analysis to the sLOX structure 1YGE and to human 5-LOX structure 3O8Y using different target center and box dimensions.

Free Binding Energy (Kcal/mole)											
Comp.	Soybean sLOX 1YGE					Human 5-LOX 3O8Y					IC_50_
	Center: x: 26.37, y: 42.69, z: 12.36			Center: x: 20.37, y: 46.69, z: 8.36		Center: x: 8.59, y: 22.65, z: −1.02			Center: x: −3.57, y: 17.66, z: 8.36	Center: x: −12.50, y: 75.50, z: 0.50	
	Box 20	Box 30	Box 35	Box 37 × 27 × 22	Box 36 × 20 × 20	Box 20	Box 30	Box 35	Box 20 × 25 × 20	Box 35 × 25 × 27	
2			−7.11			−7.42					22.50
4			−7.00								31.25
5	+90.68		−8.20				−5.84				22.50
8			−7.83								90.00
9	+90.14	−6.34	−8.26	−5.68	−1.08	−8.28	−7.77	−8.25	−5.49	−6.09	2.50
11	−95.16	−6.41	−6.24	−6.01	+5.37	−8.09	−7.77	−6.51	−5.11	−6.63	156.25
13			−7.75				−7.57	−6.75			25.00
14						−7.09		−8.51			31.25
15			−7.32								96.25
NDHDA			−7.00								
A.A			−6.63	−5.04	−4.37	−3.31		−6.72	−4.58	−4.97	

## Supplementary Materials

The Supplementary Materials are available online at http://www.mdpi.com/1420-3049/23/7/1621/s1.
