# Supplementary material for: Application of Docking Analysis in the Prediction and Biological Evaluation of the Lipoxygenase Inhibitory Action of Thiazolyl Derivatives of Mycophenolic Acid
Source: Molecules. 2018 Jul 3;23(7):1621. doi: 10.3390/molecules23071621 (PMC6099768; doi:10.3390/molecules23071621)

Display Report

Analysis Info

Analysis Name	D:\Data\Chizhov\Krayushkin\Melekhina\14_&clb.d	Acquisition Date	19.06.2018 16:11:48
Method	tune_wide.m	Operator	BDAL@DE
Sample Name	/MVGR 14	Instrument / Ser#	micrOTOF 10248
Comment	CH3CN 100 %, dil. 200, calibrant added		

Acquisition Parameter

Source Type	ESI	Ion Polarity	Positive	Set Nebulizer	0.4 Bar
Focus	Not active			Set Dry Heater	180 °C
Scan Begin	50 m/z	Set Capillary	4500 V	Set Dry Gas	4.0 l/min
Scan End	3000 m/z	Set End Plate Offset	-500 V	Set Divert Valve	Waste

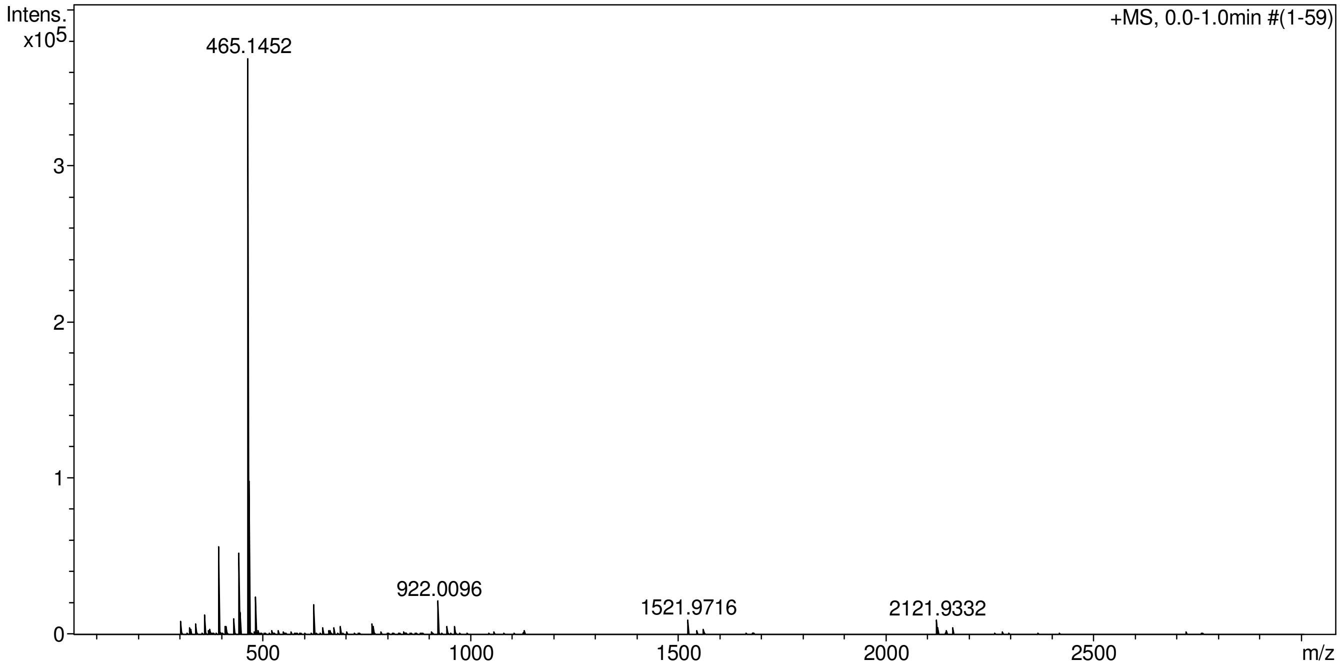

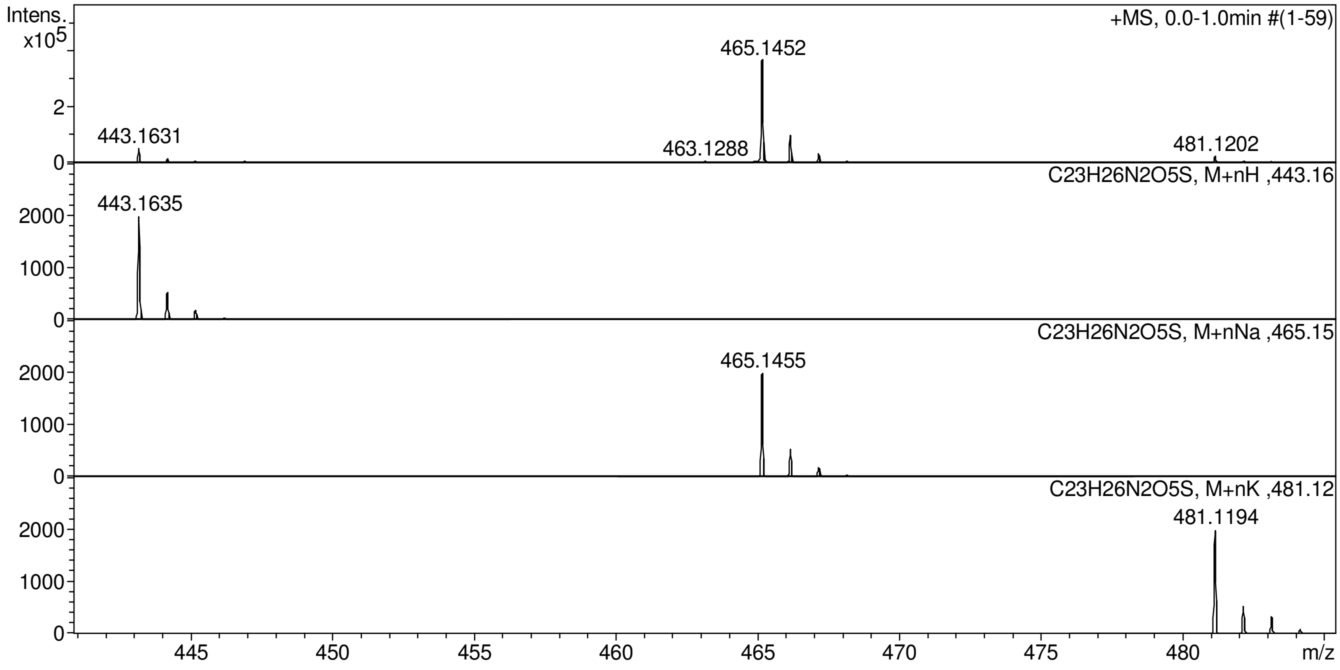

Supplement: Supplementary file 1 [file molecules-23-01621-s001.zip › Supplementary/HRMS/14.{MSHR}4500_wide.pdf]
