# Supplementary figures and images for: Application of Docking Analysis in the Prediction and Biological Evaluation of the Lipoxygenase Inhibitory Action of Thiazolyl Derivatives of Mycophenolic Acid
Source: Molecules. 2018 Jul 3;23(7):1621. doi: 10.3390/molecules23071621 (PMC6099768; doi:10.3390/molecules23071621)

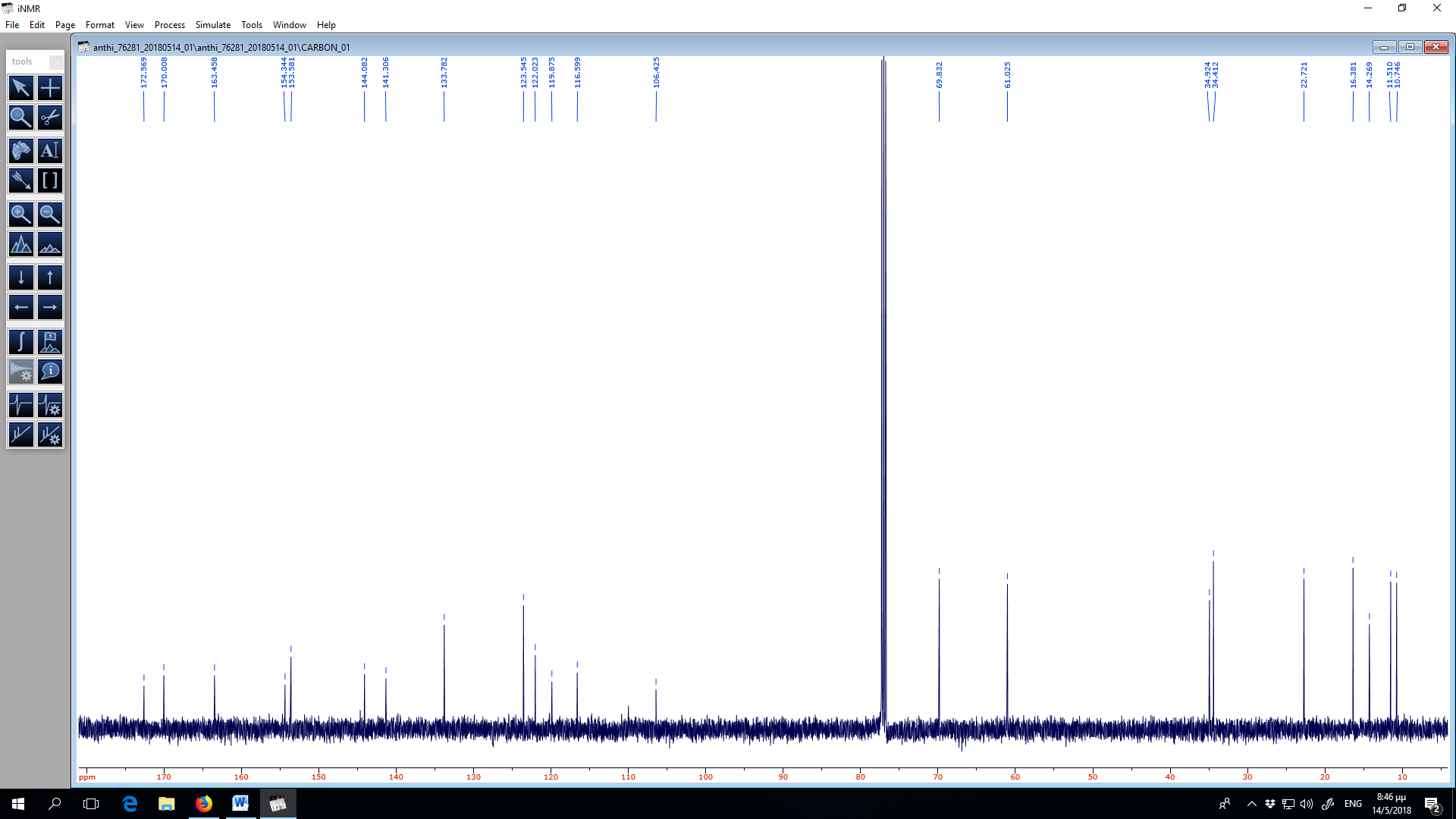


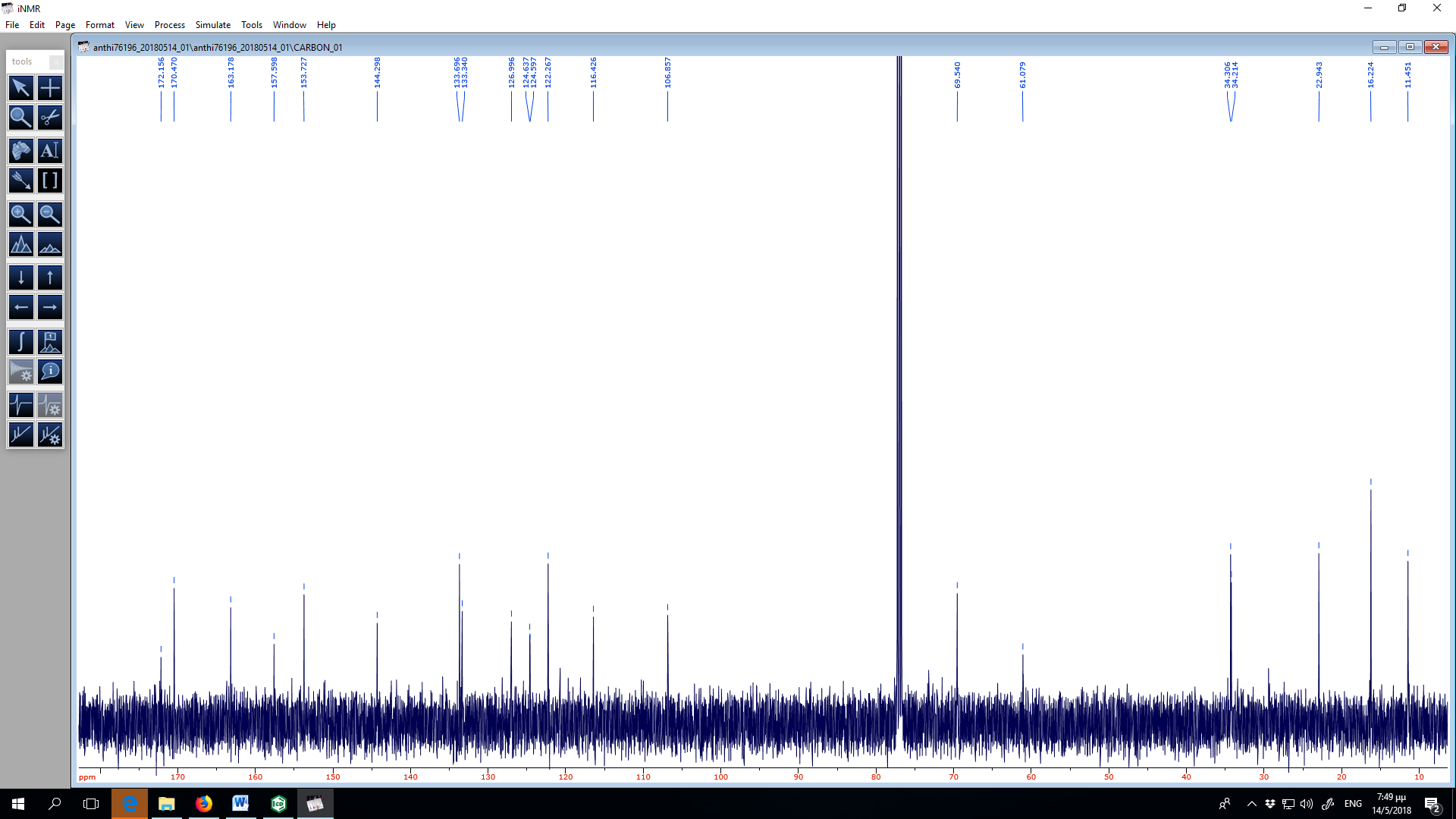


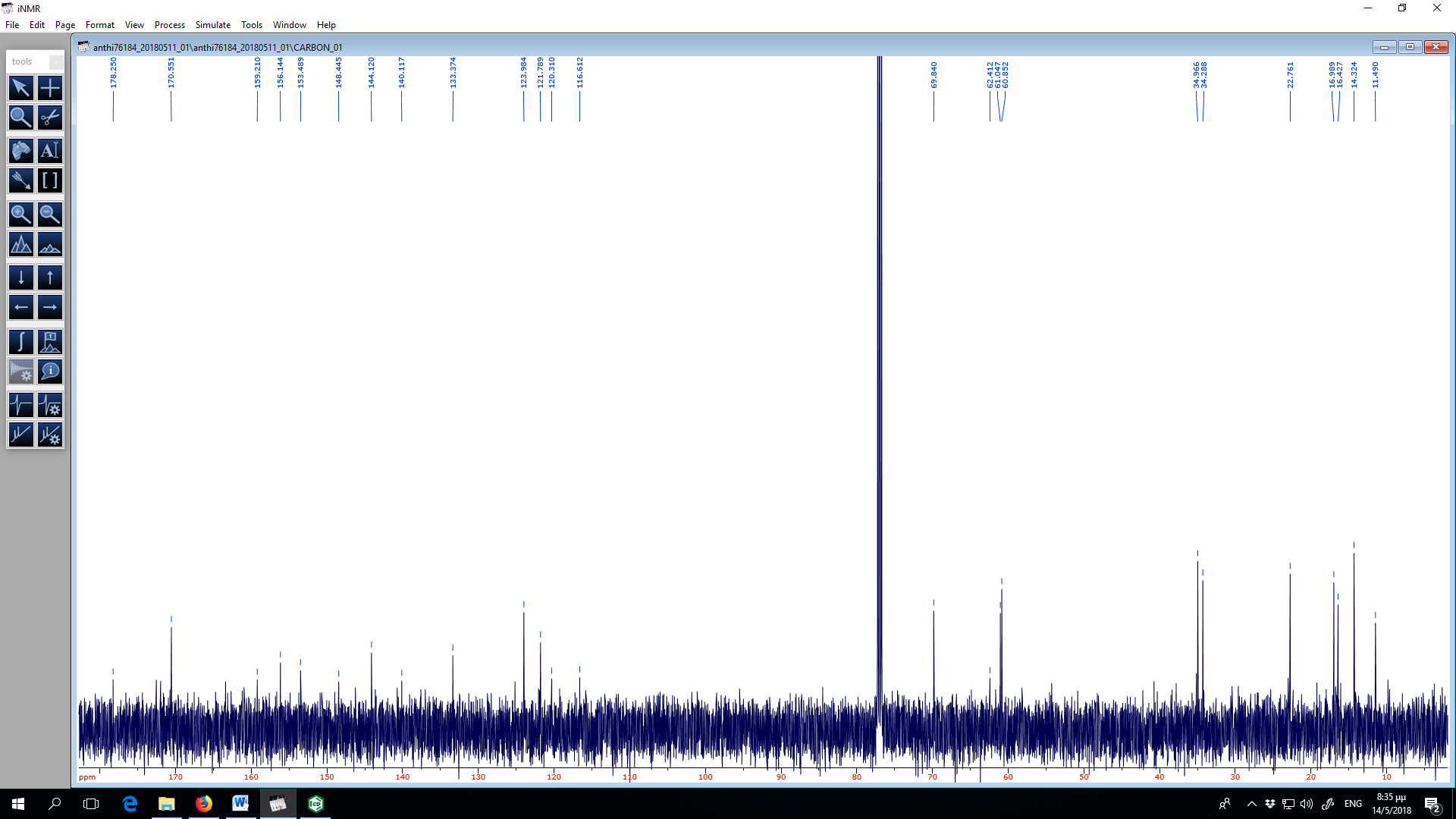


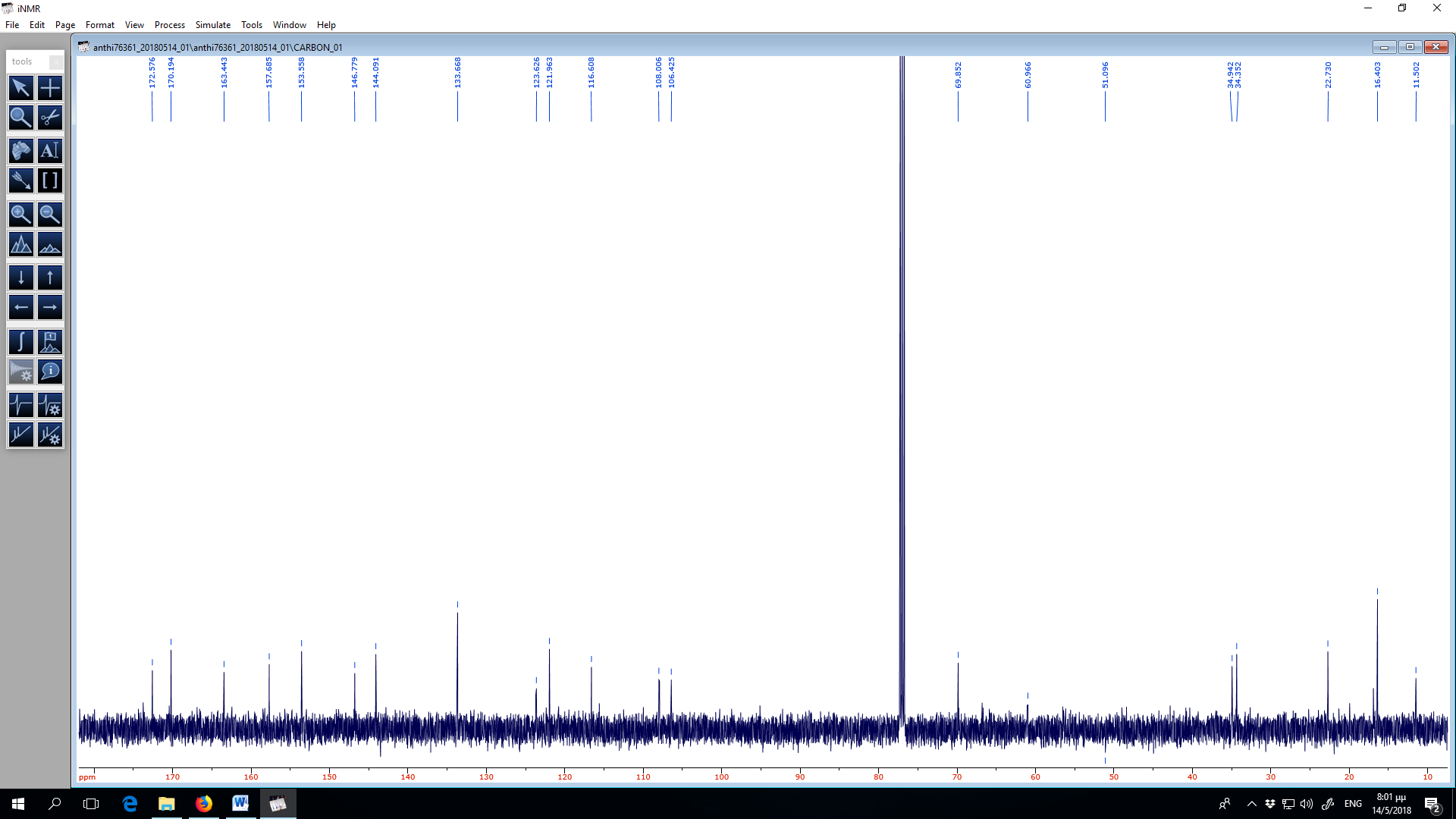


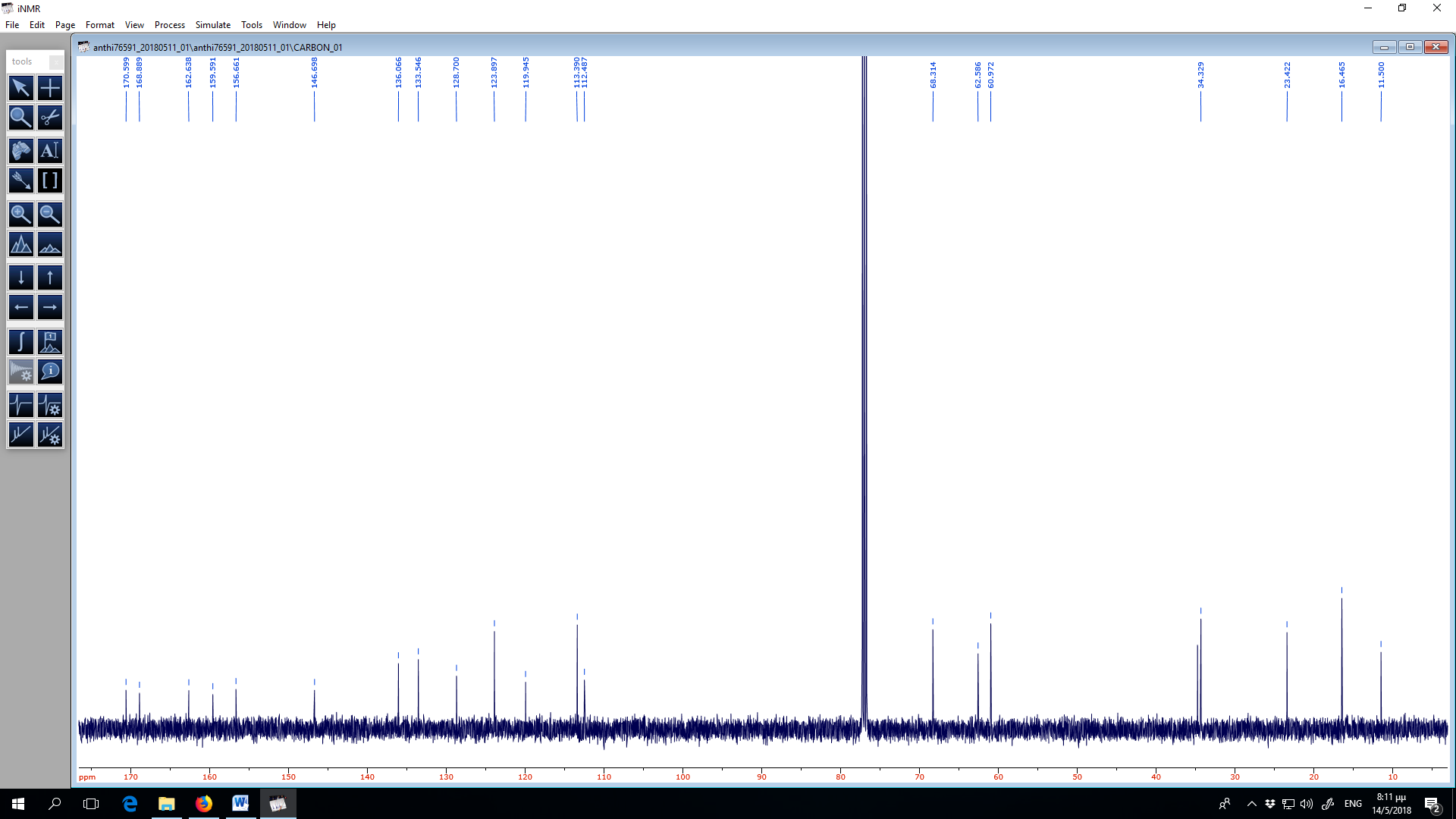


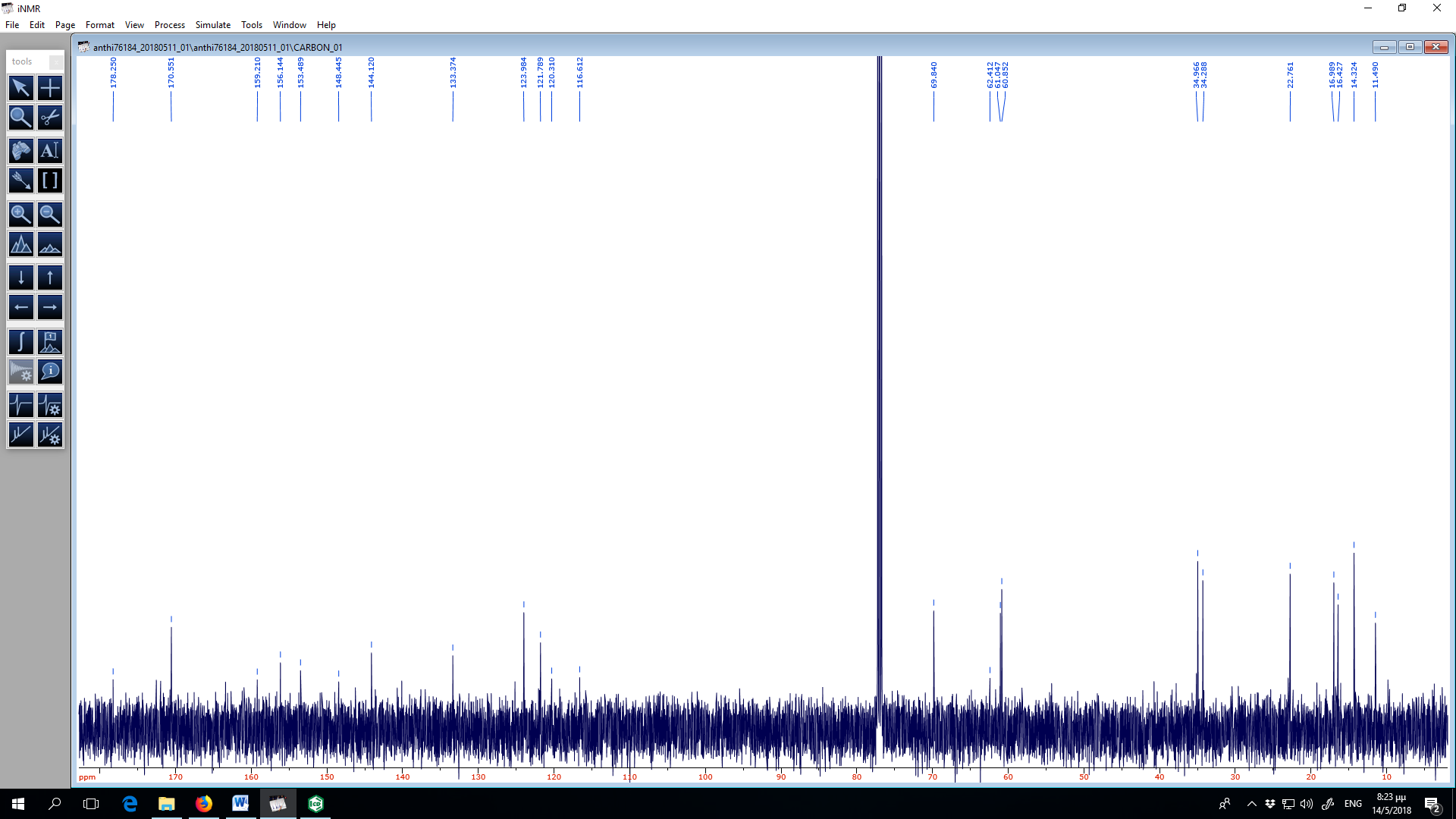


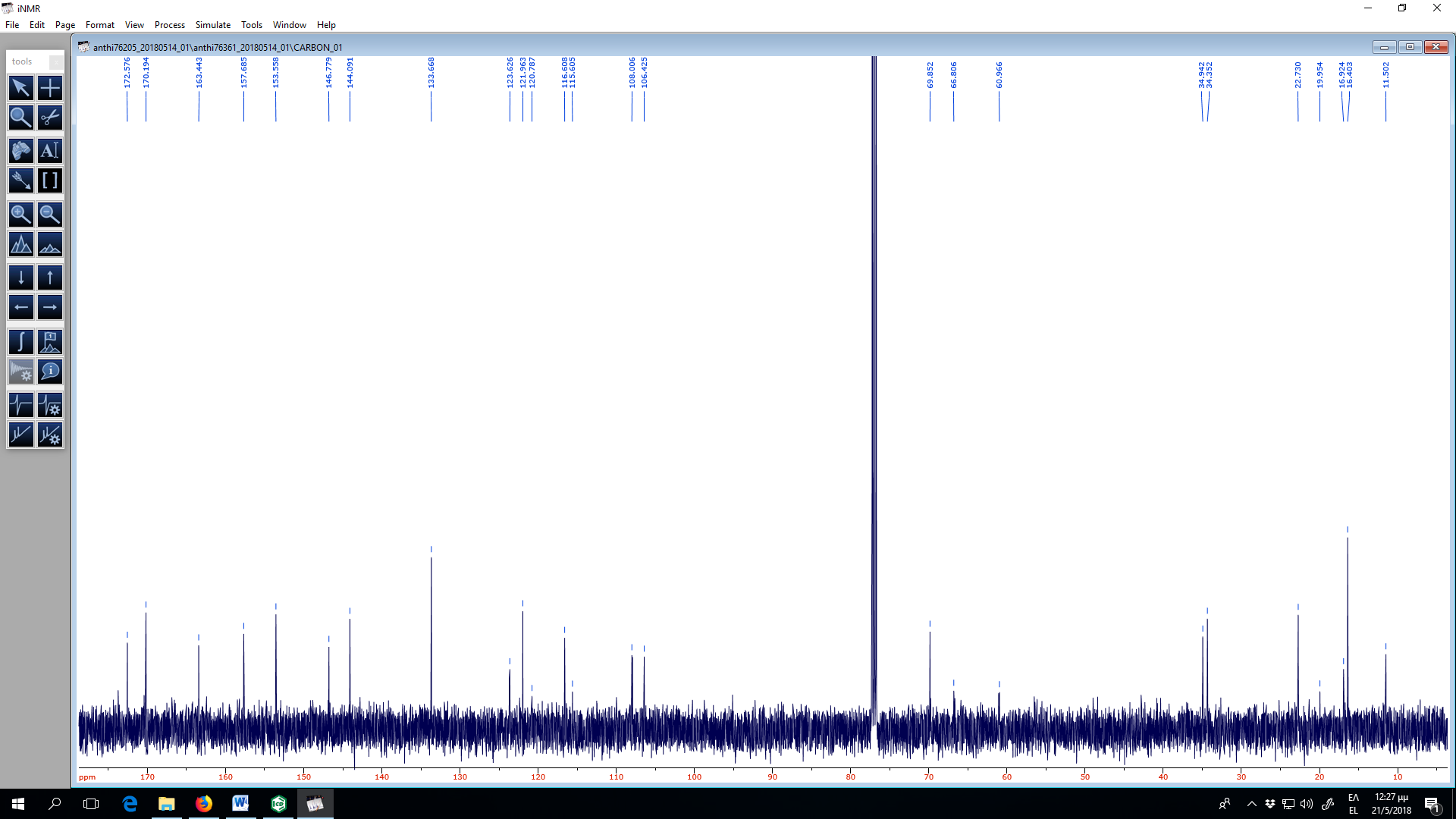


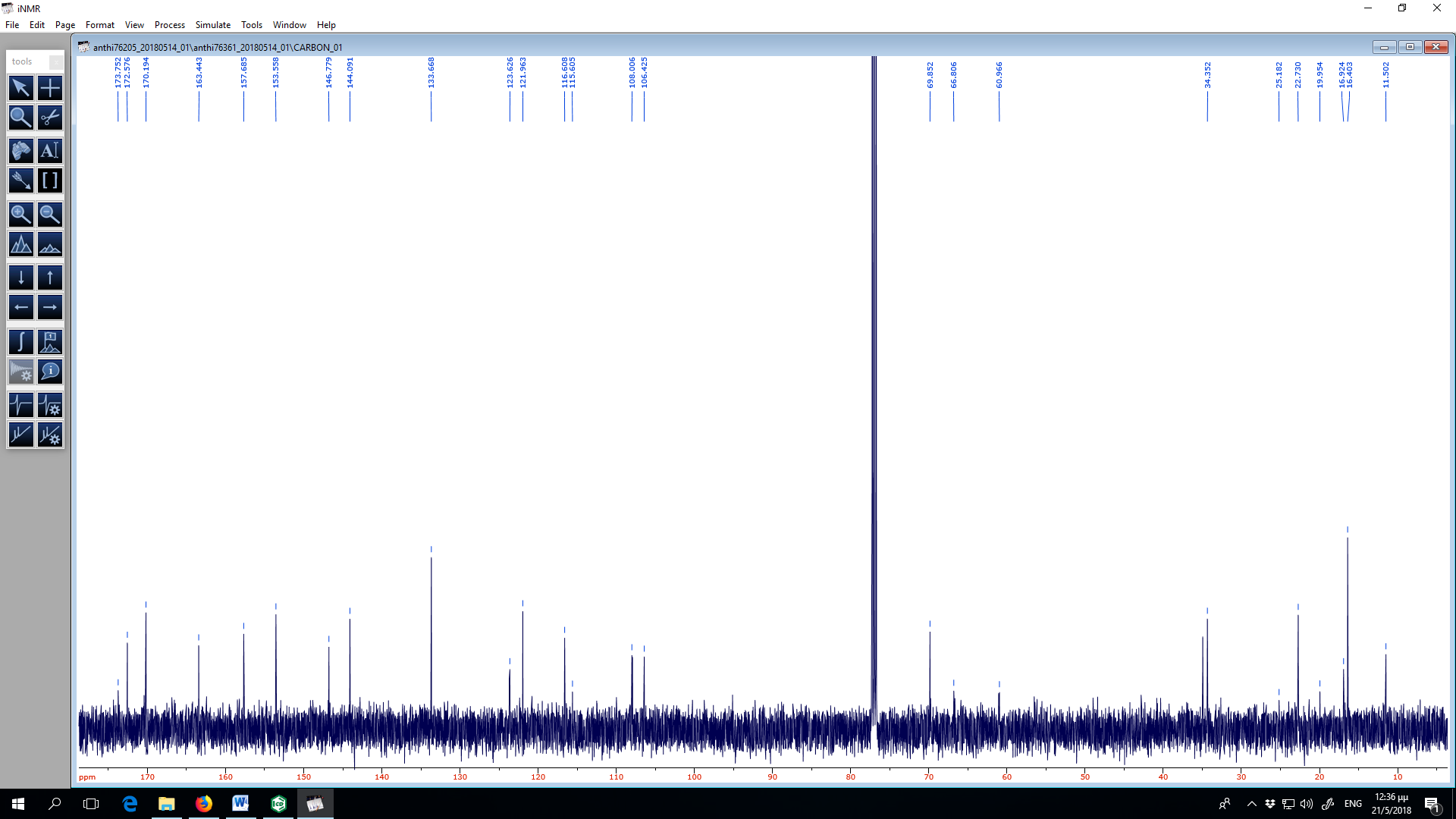


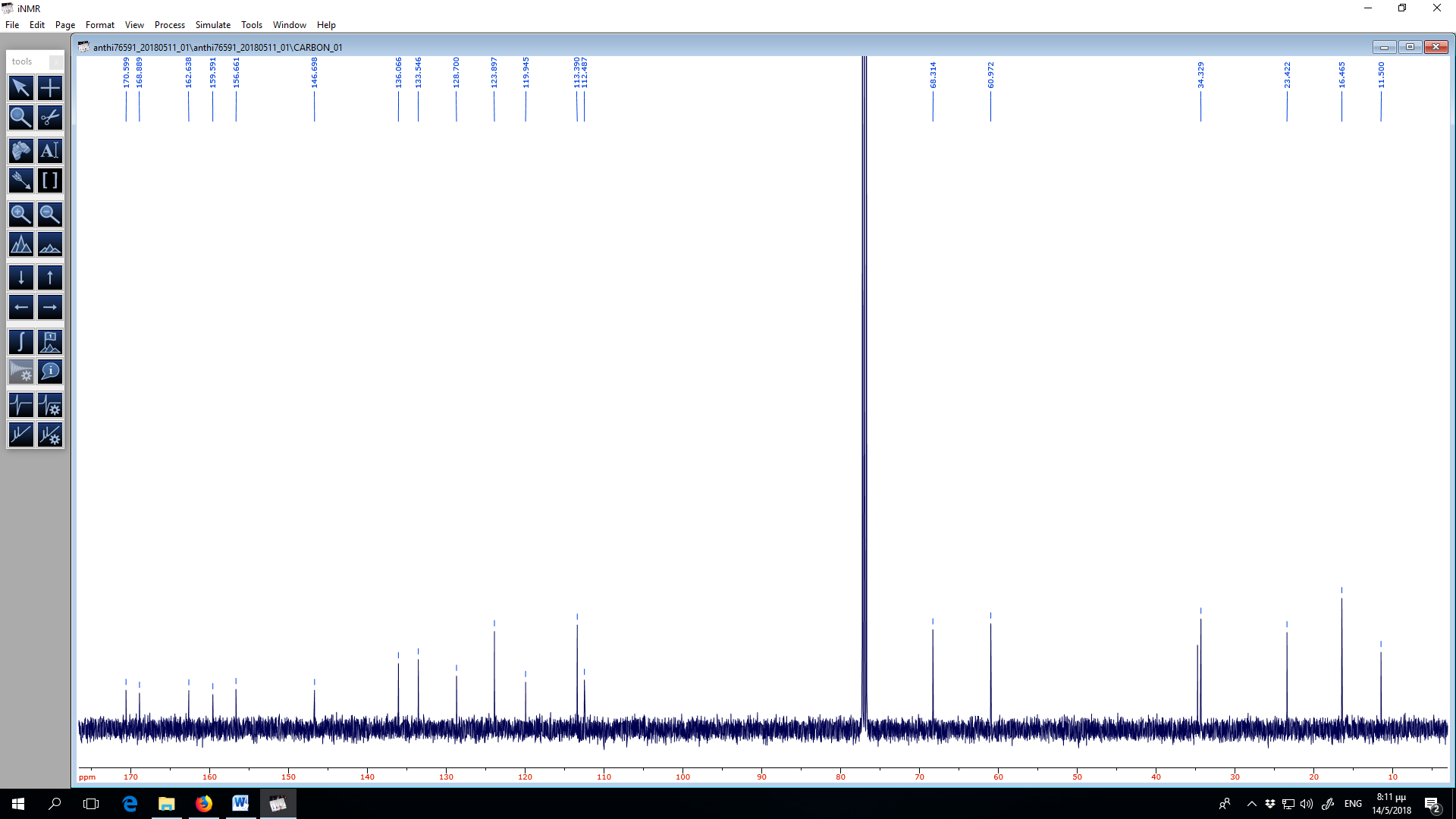


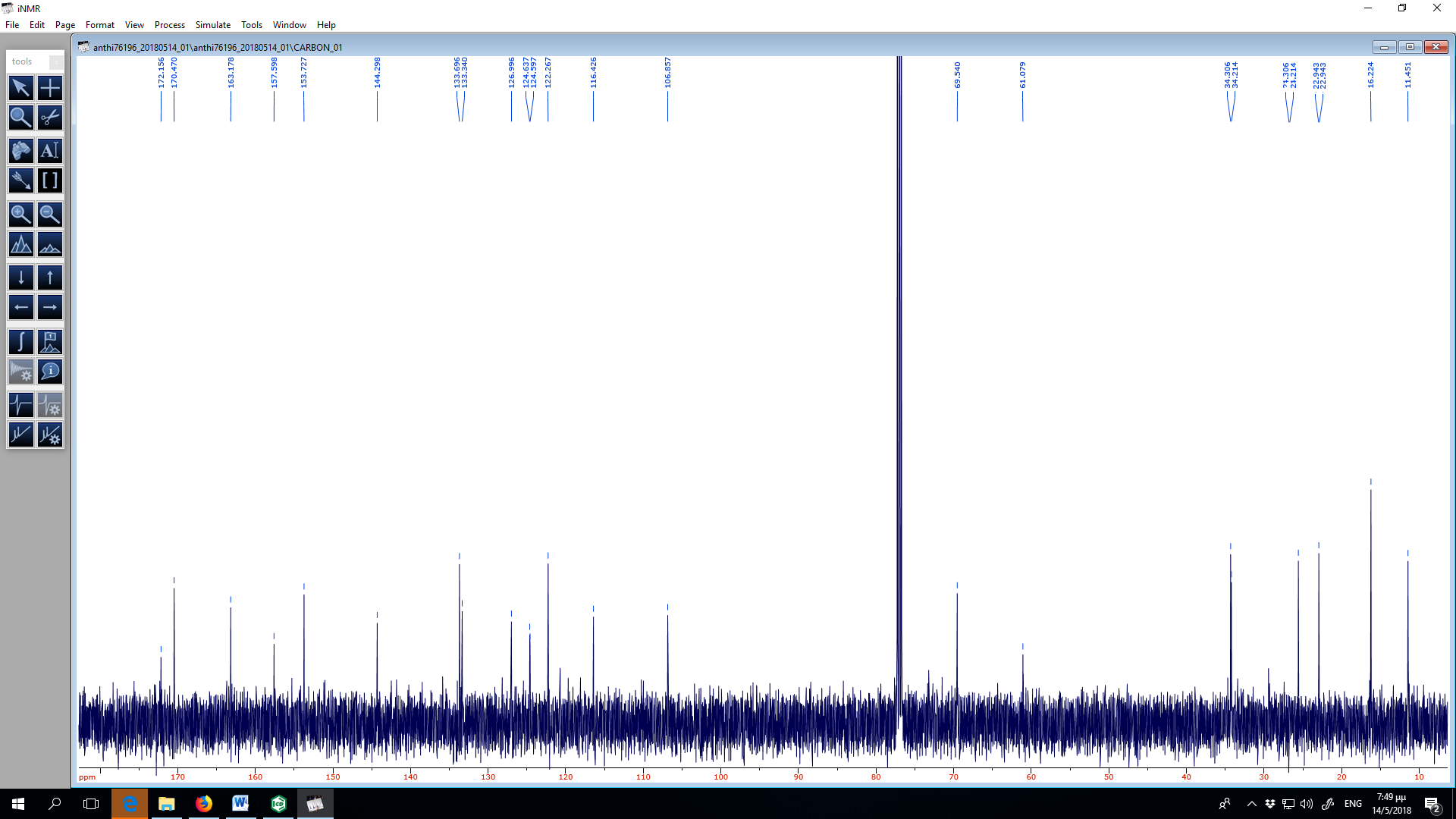


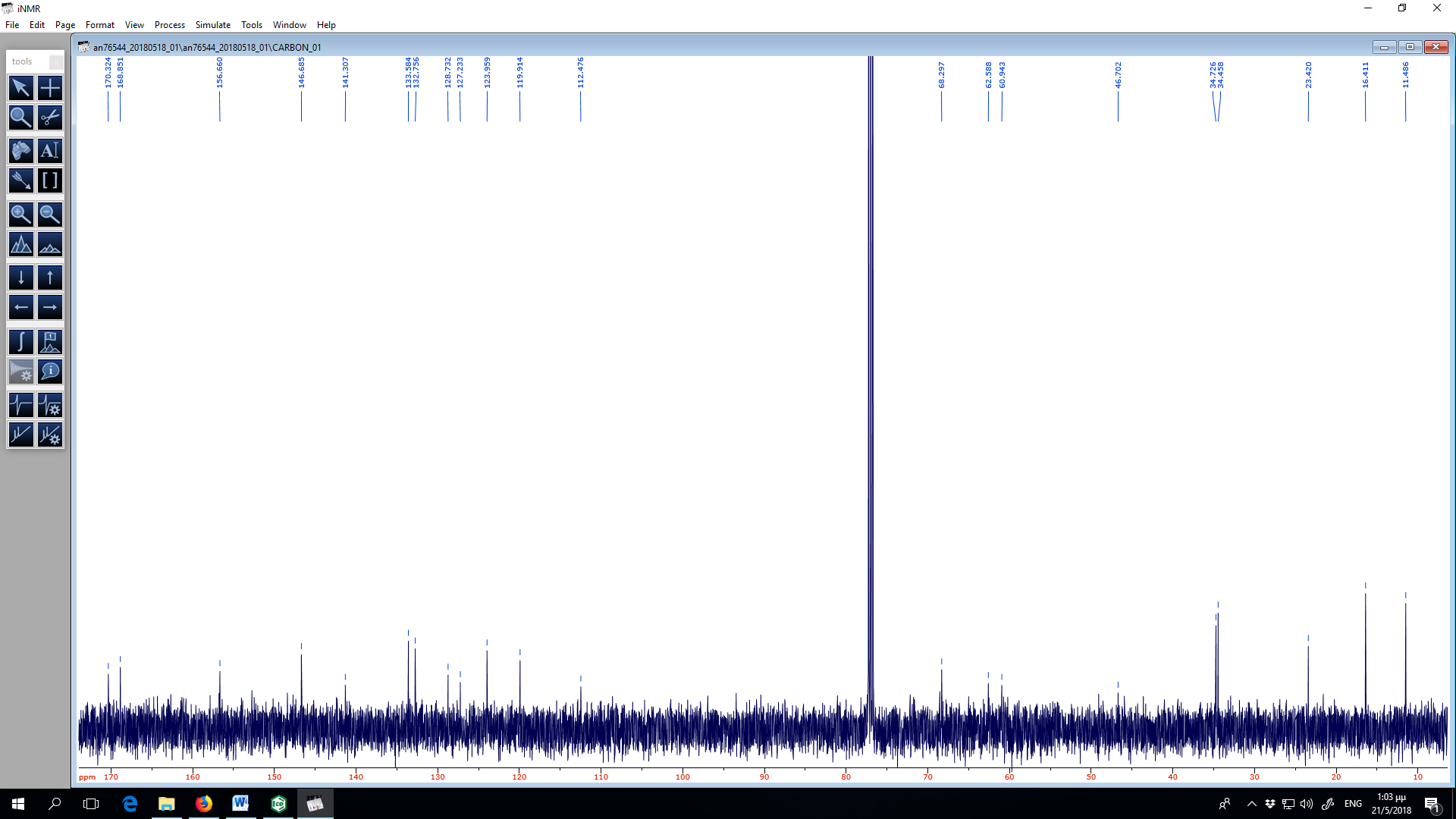


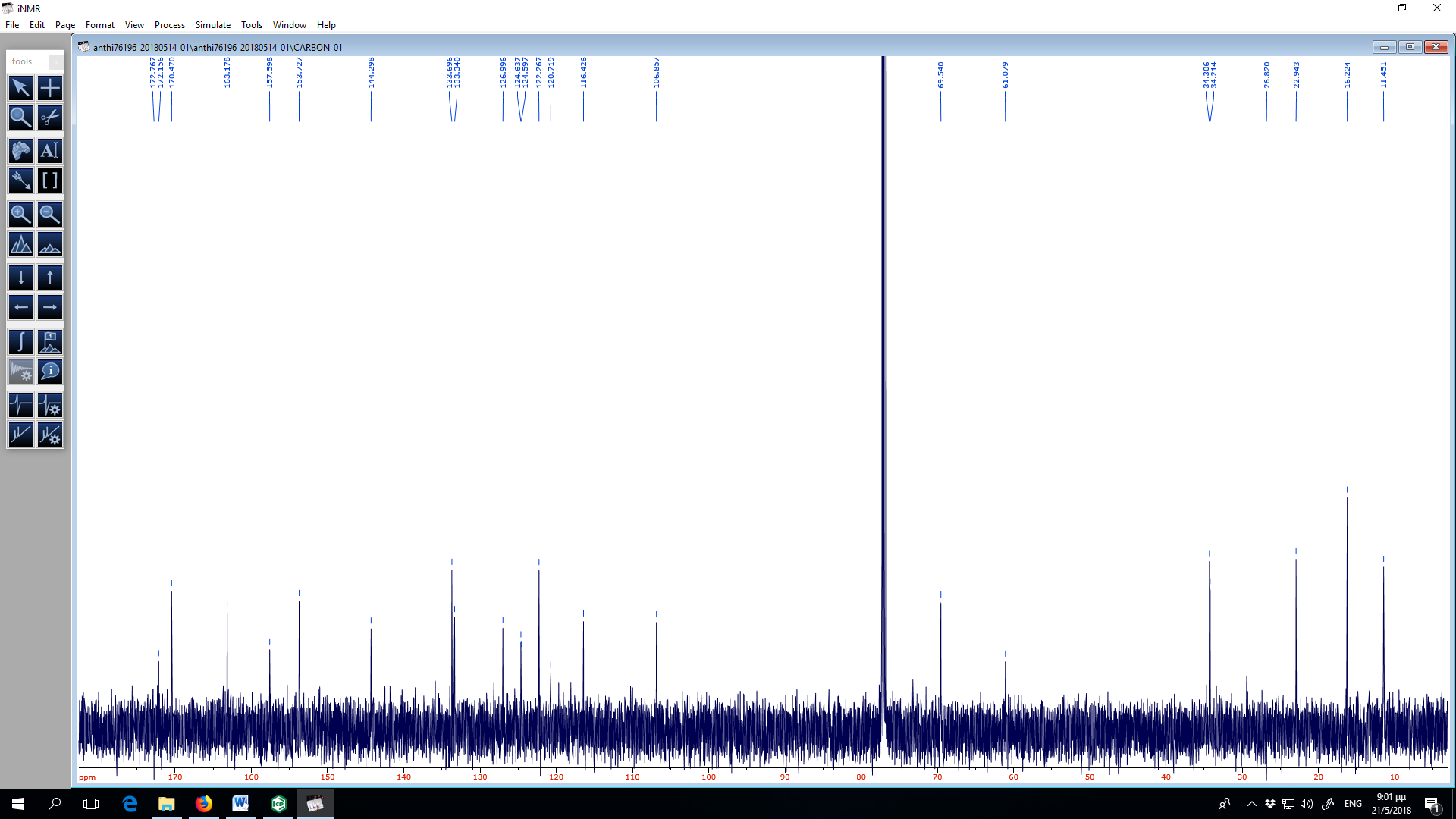


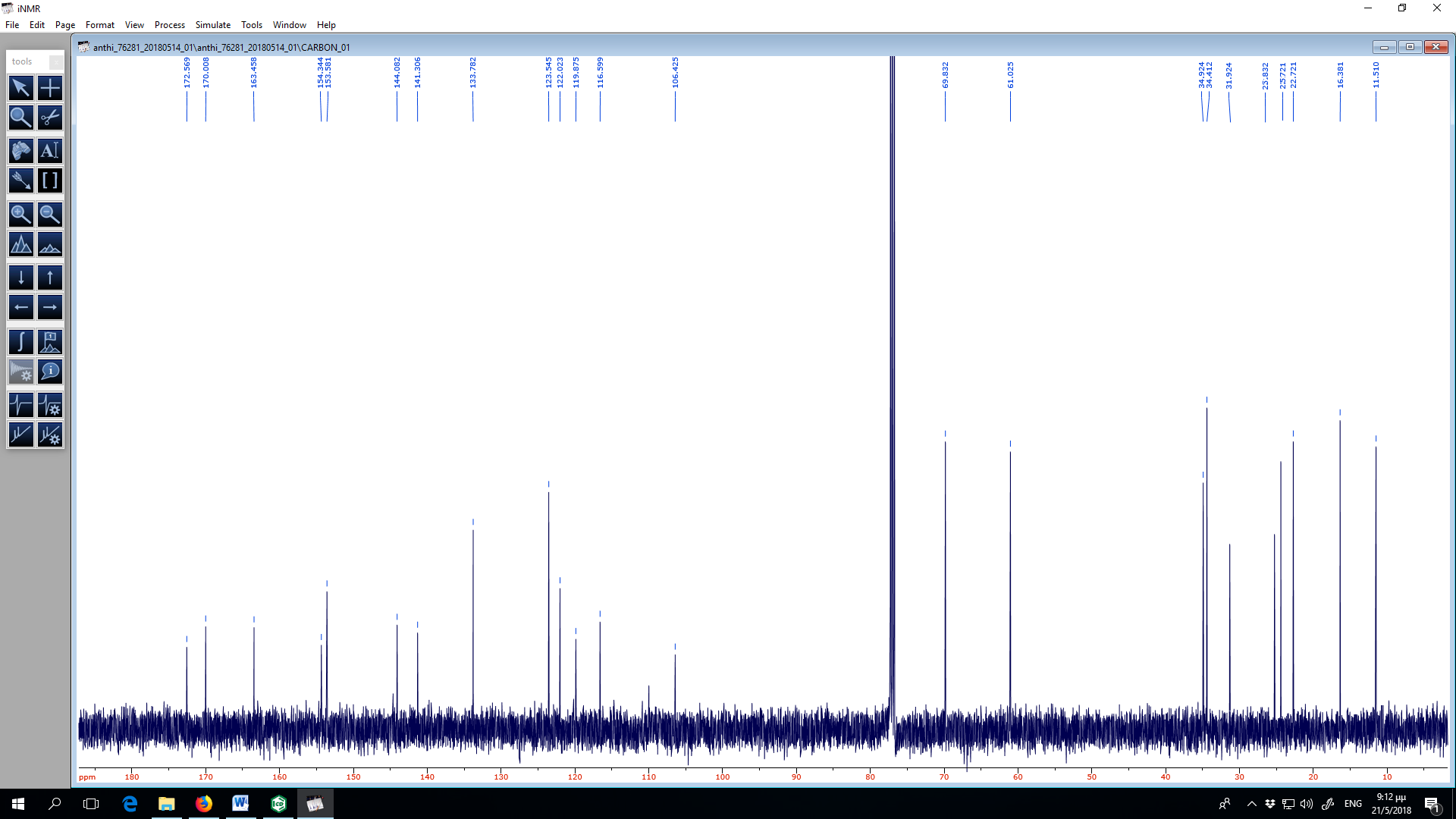


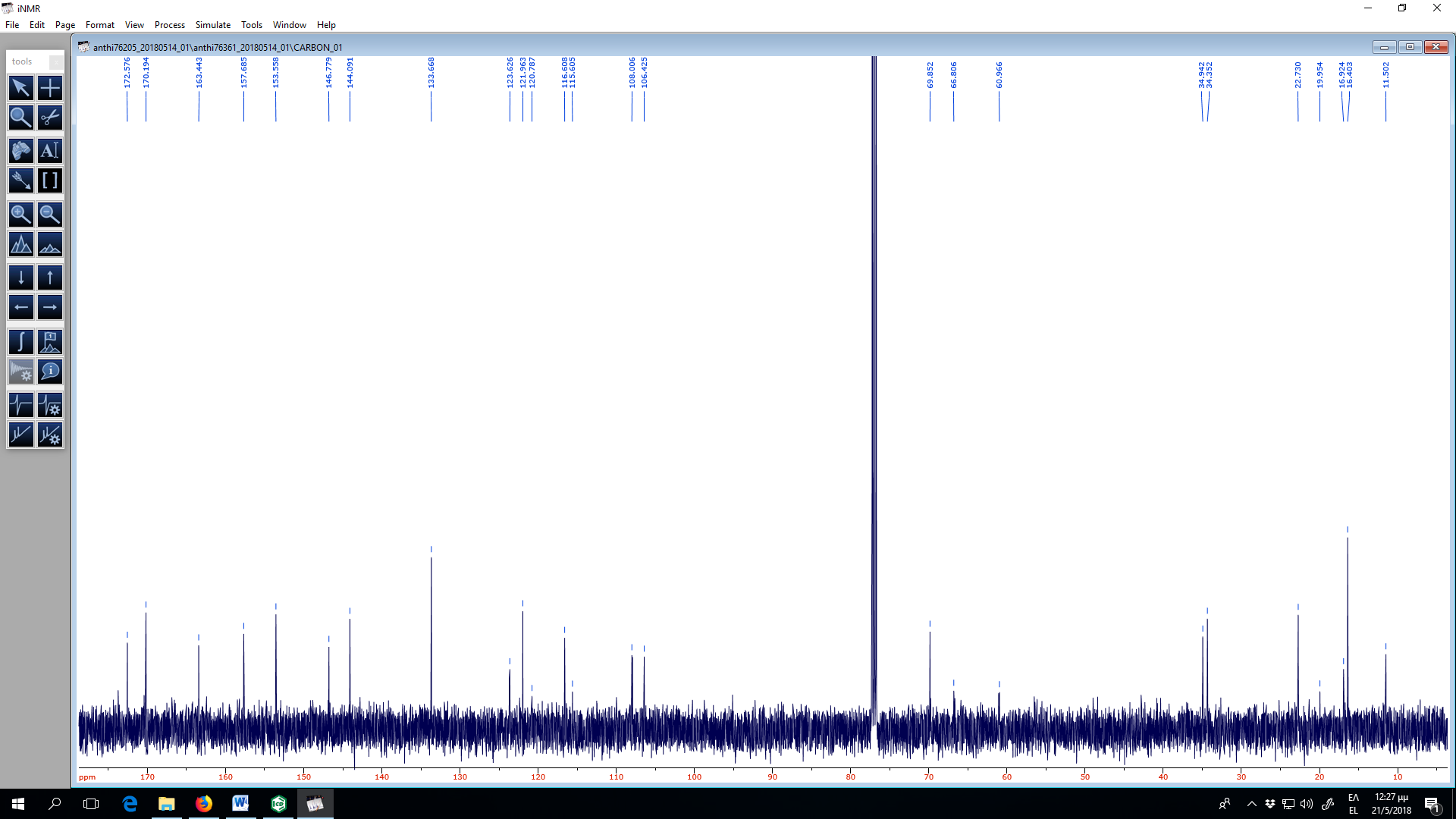


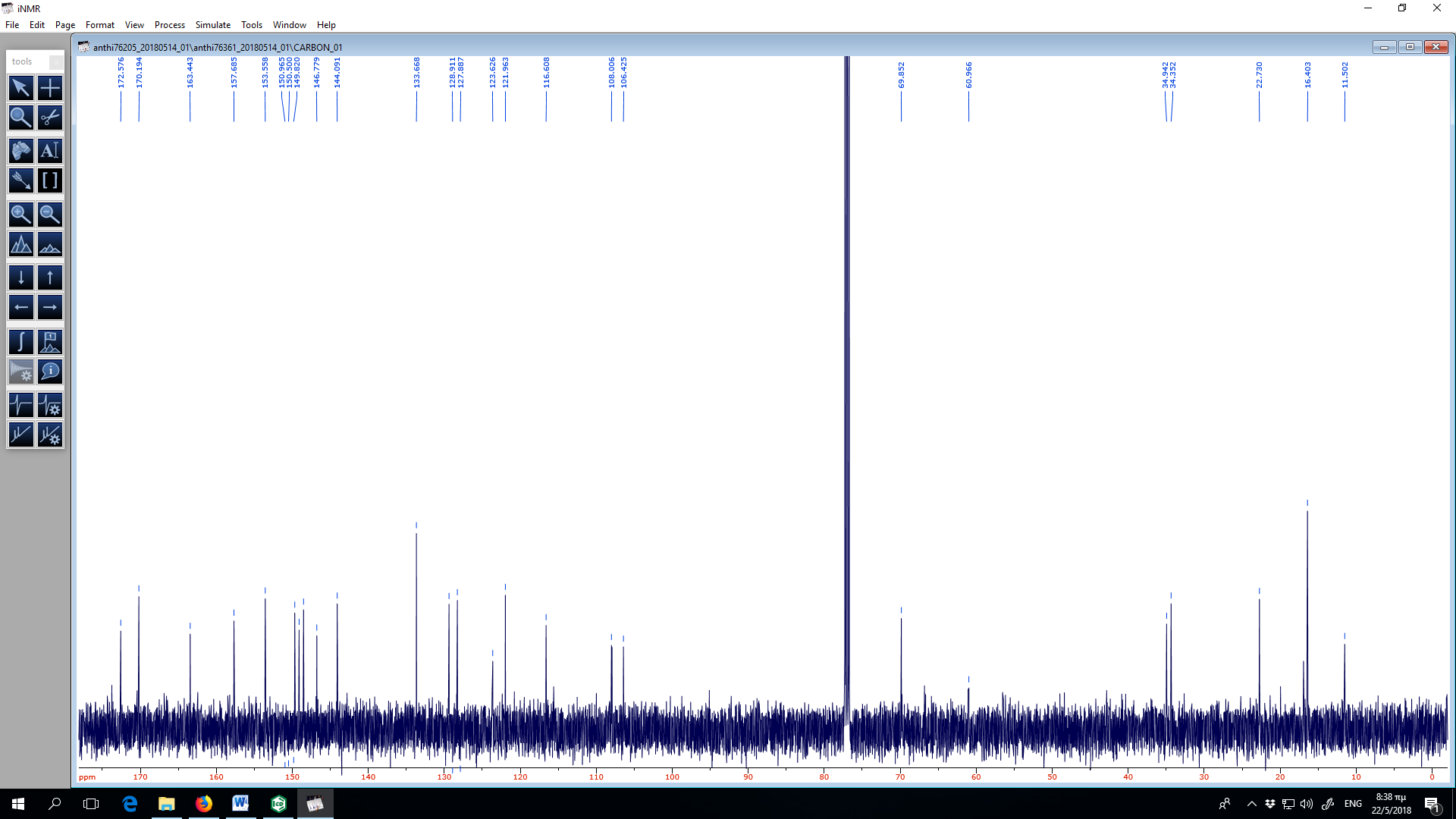


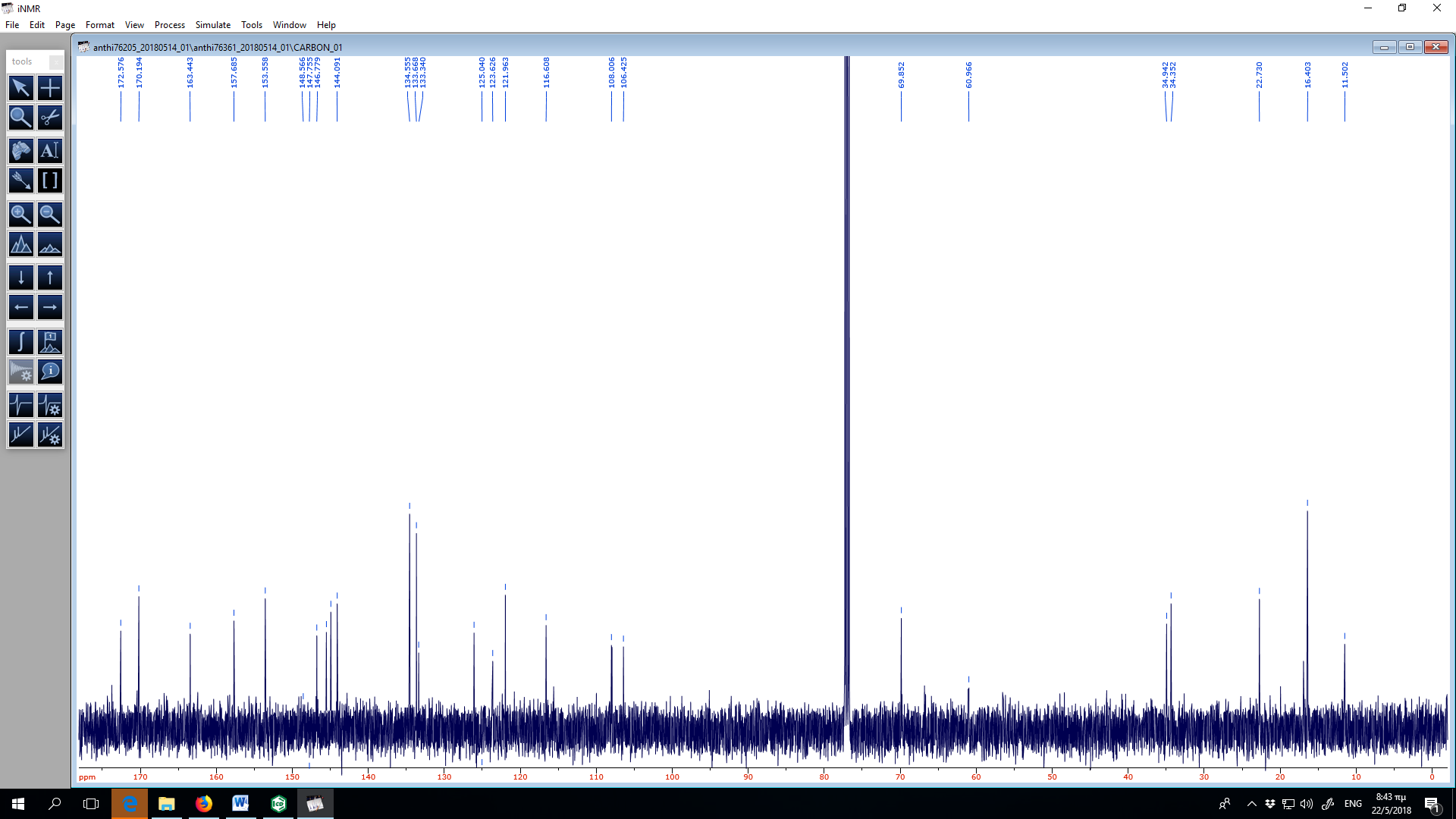

Supplement: Supplementary file 1 [file molecules-23-01621-s001.zip › Supplementary/C-nmr.docx]

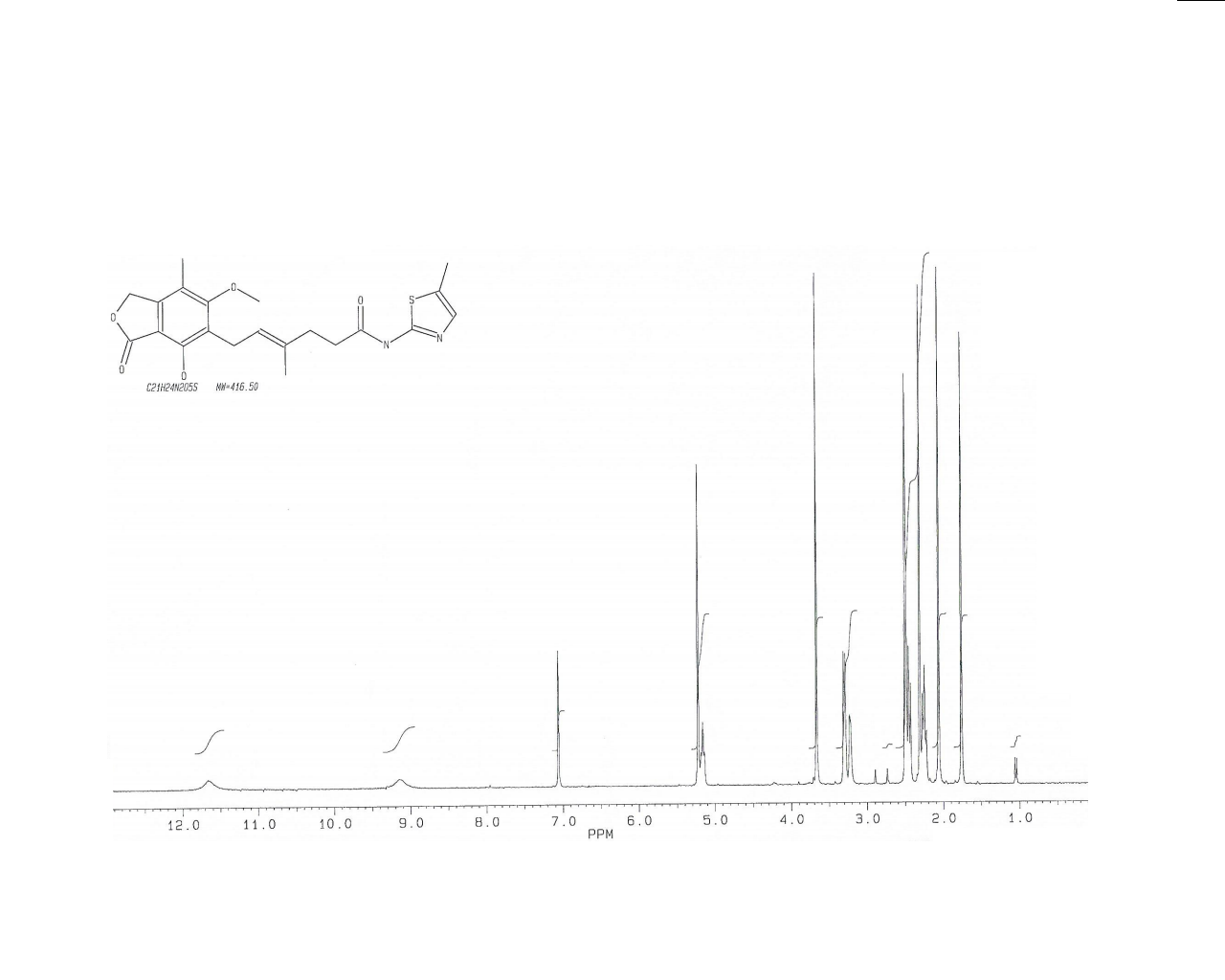

Supplement: Supplementary file 1 [file molecules-23-01621-s001.zip › Supplementary/H-NMR/1-76196 600.tif]

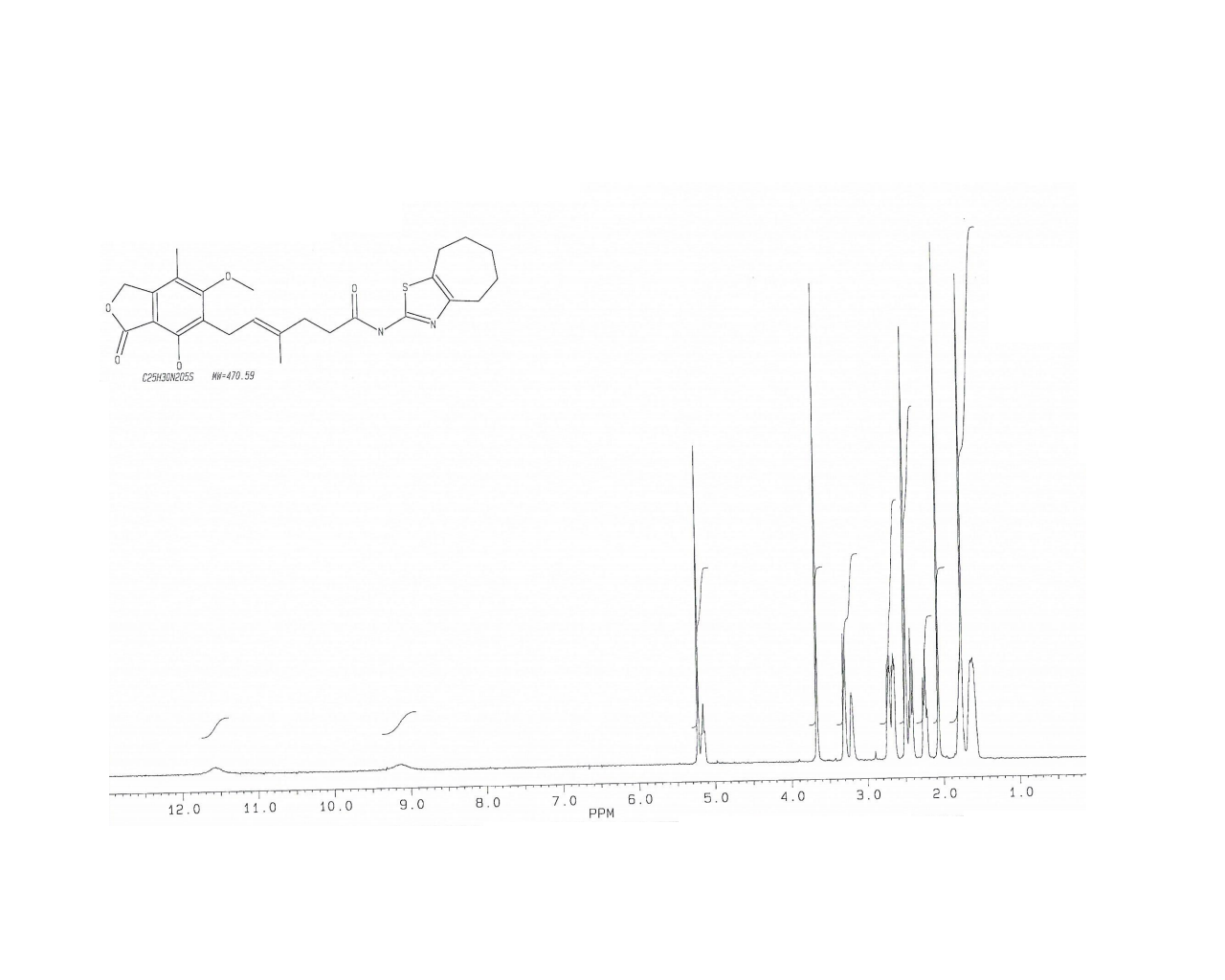

Supplement: Supplementary file 1 [file molecules-23-01621-s001.zip › Supplementary/H-NMR/10-76326 600.tif]

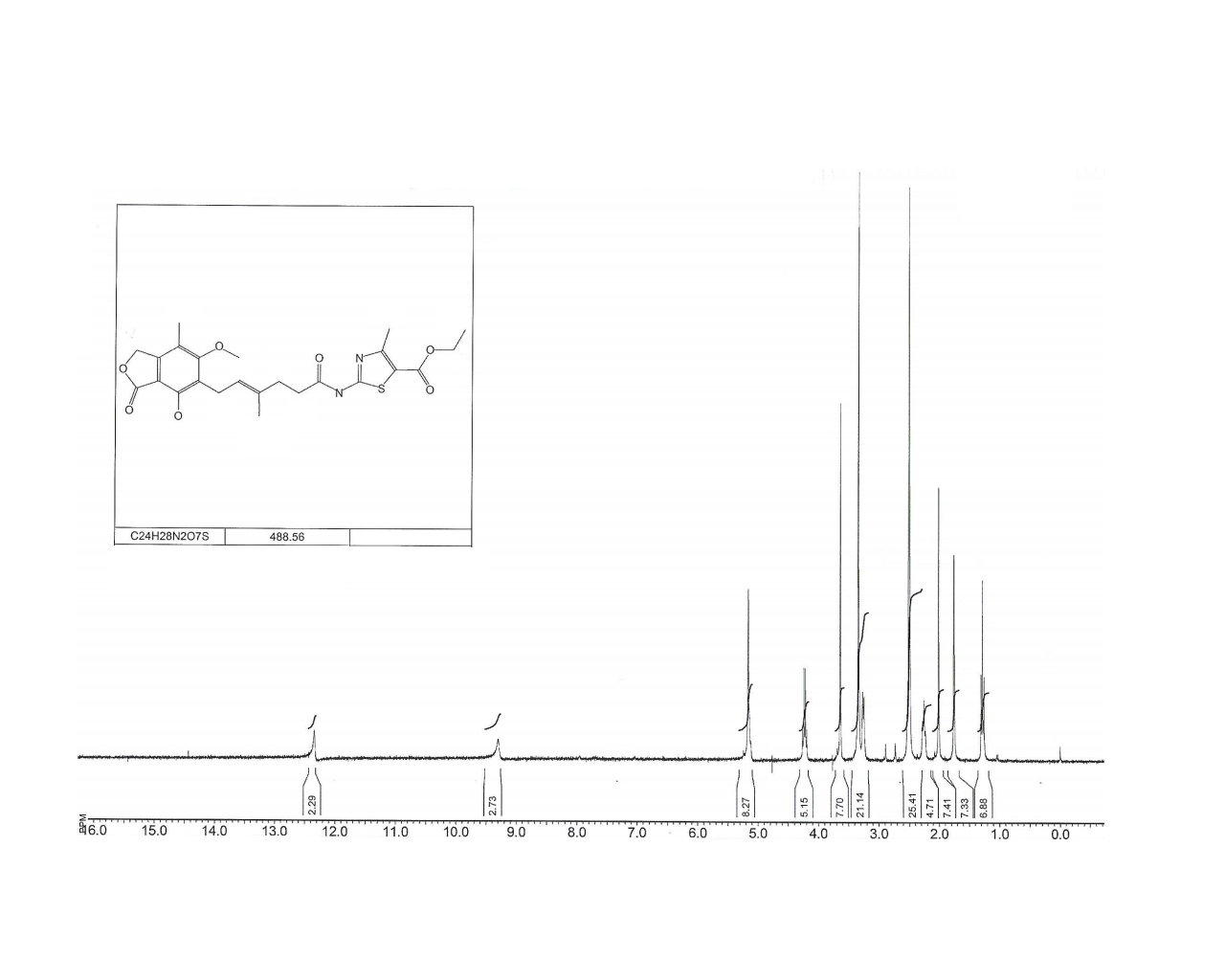

Supplement: Supplementary file 1 [file molecules-23-01621-s001.zip › Supplementary/H-NMR/11-76184 600.tif]

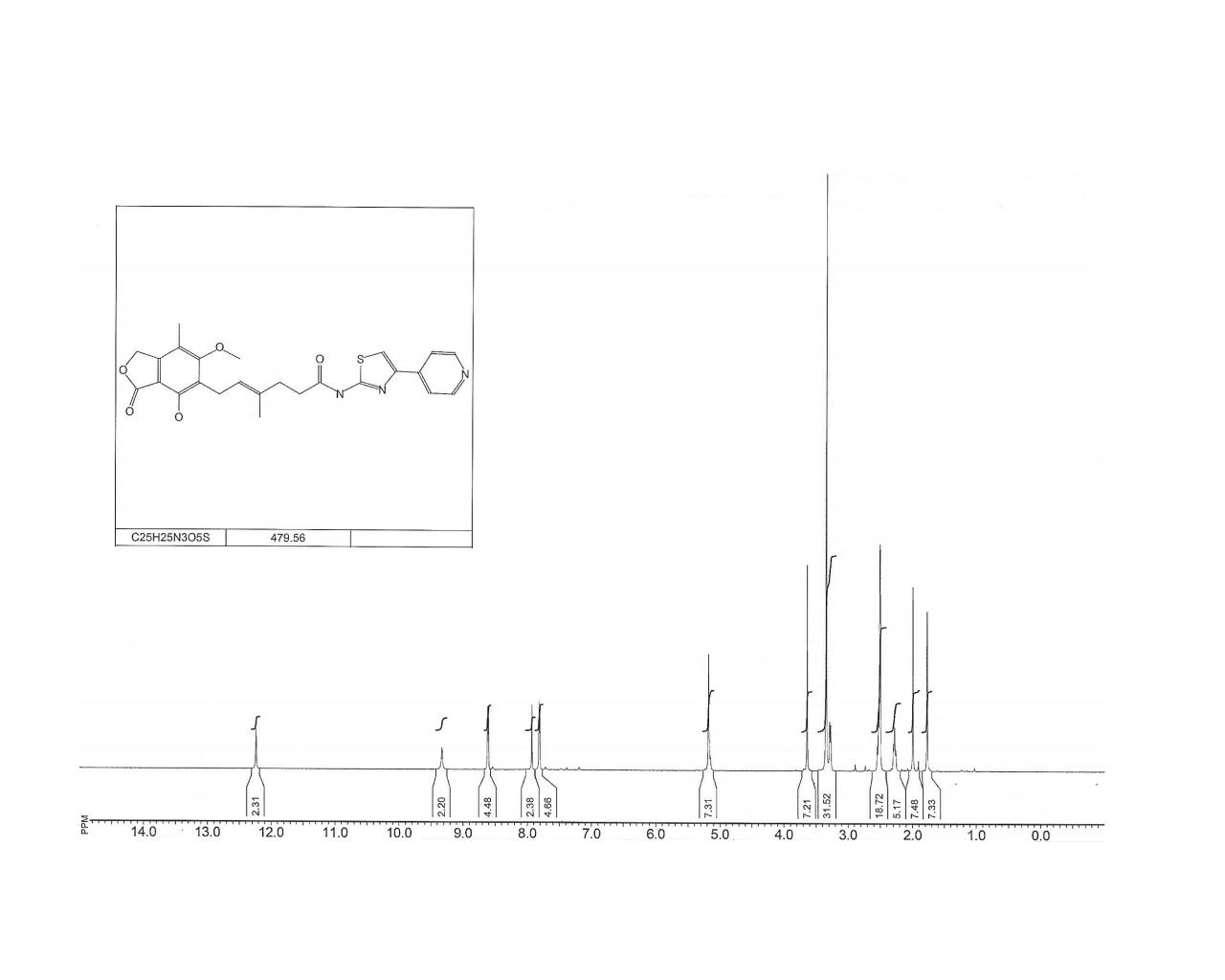

Supplement: Supplementary file 1 [file molecules-23-01621-s001.zip › Supplementary/H-NMR/12-76521 600.tif]

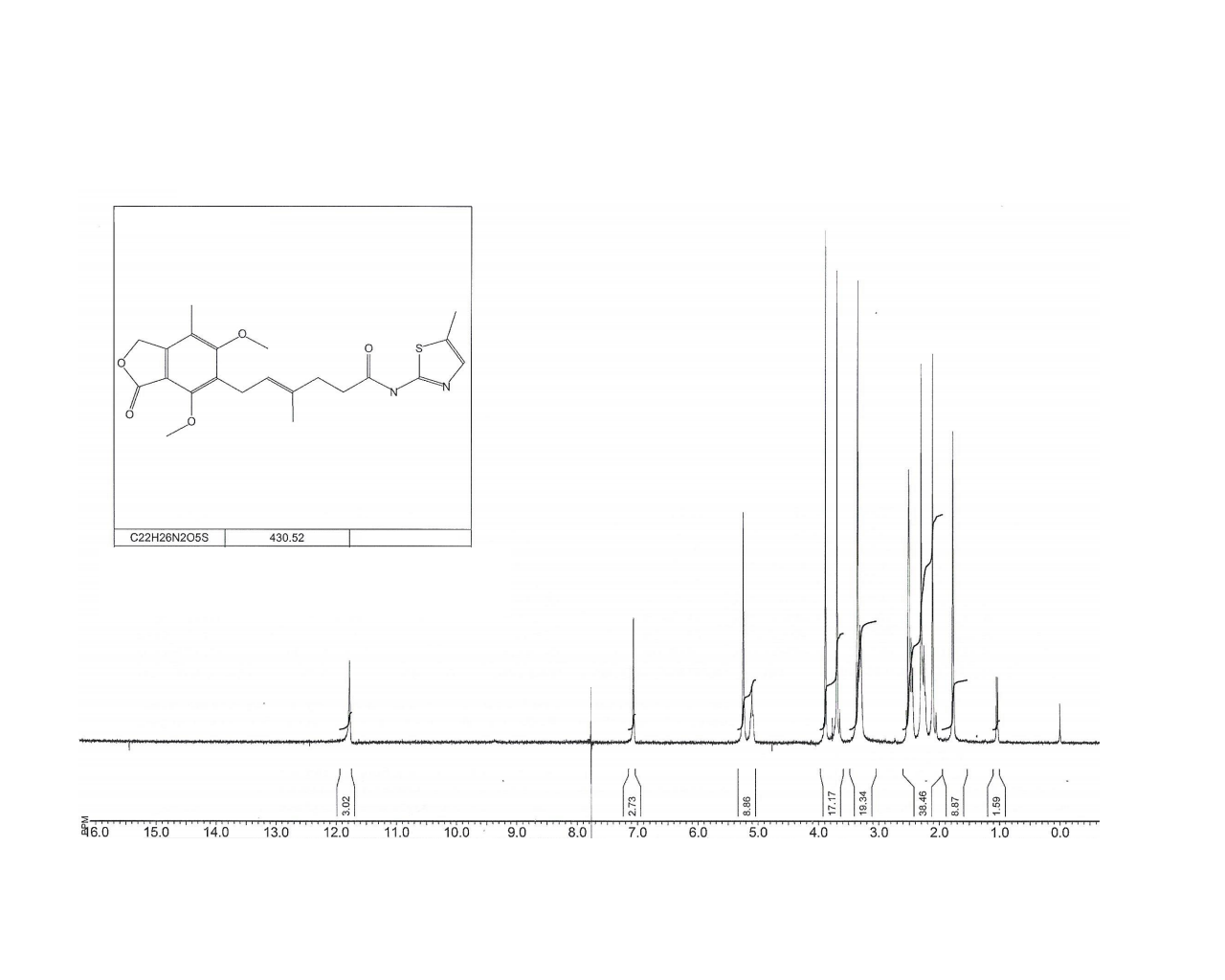

Supplement: Supplementary file 1 [file molecules-23-01621-s001.zip › Supplementary/H-NMR/13-76544 600.tif]

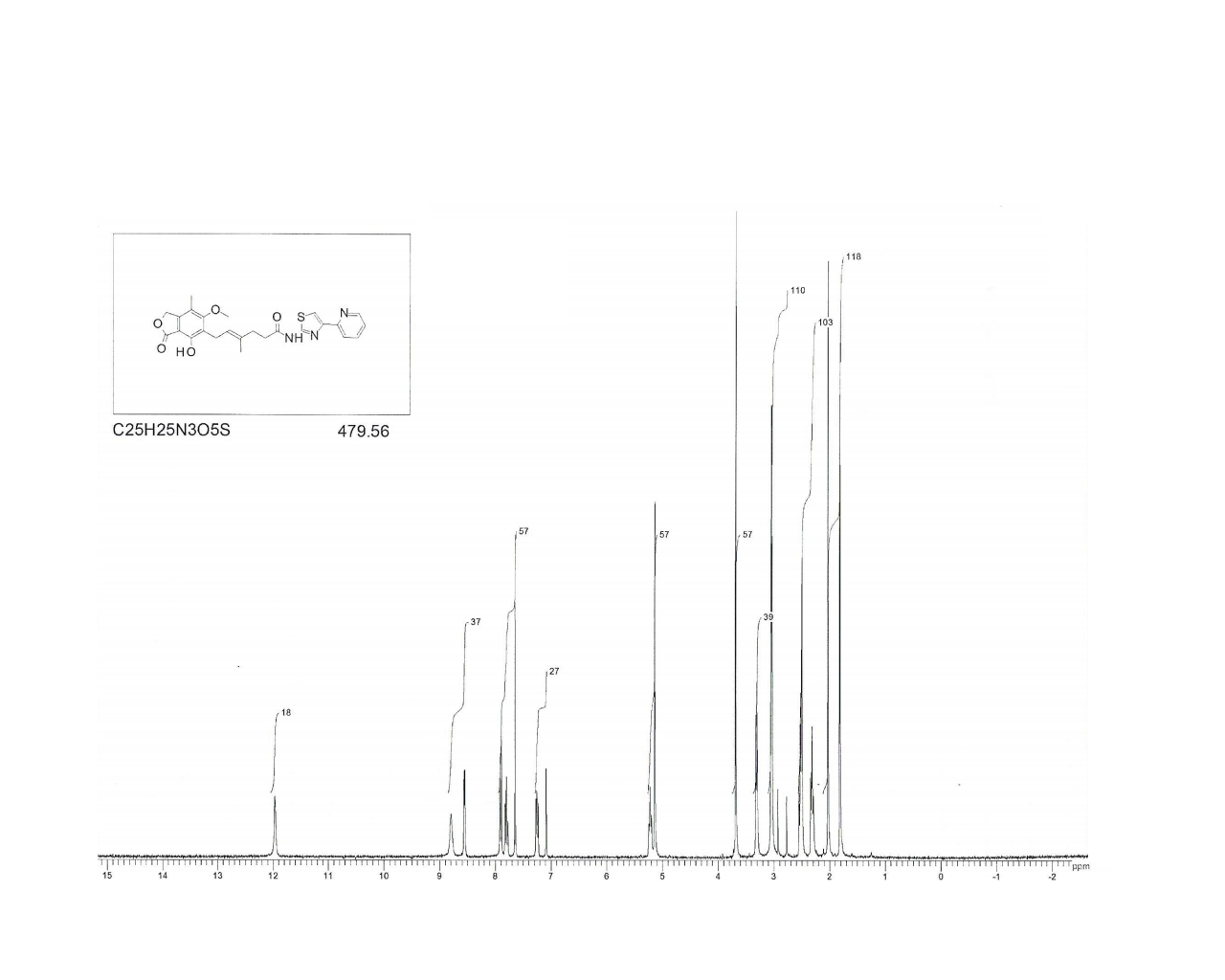

Supplement: Supplementary file 1 [file molecules-23-01621-s001.zip › Supplementary/H-NMR/14-85823 600.tif]

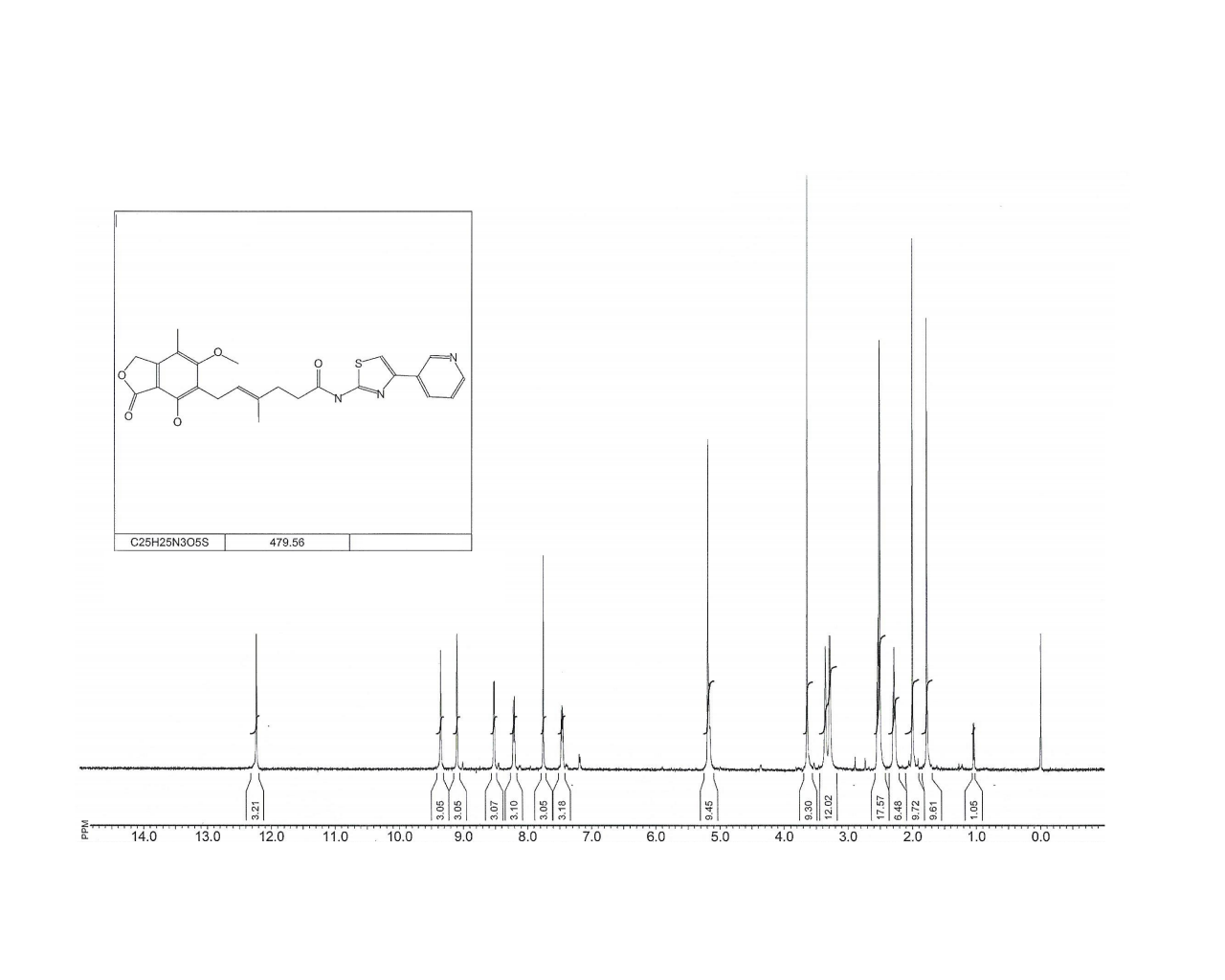

Supplement: Supplementary file 1 [file molecules-23-01621-s001.zip › Supplementary/H-NMR/15-77639 600.tif]

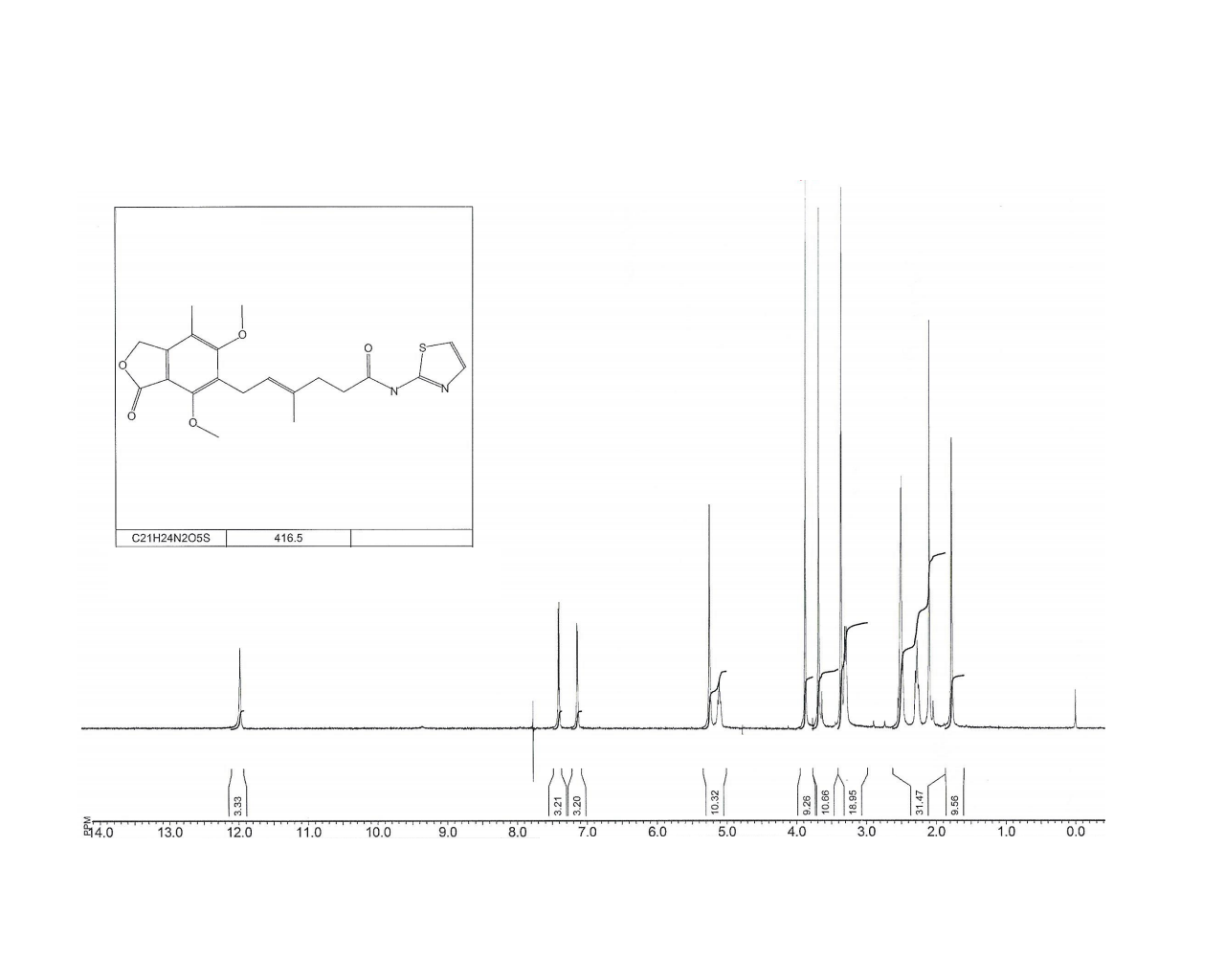

Supplement: Supplementary file 1 [file molecules-23-01621-s001.zip › Supplementary/H-NMR/16-76591 600.tif]

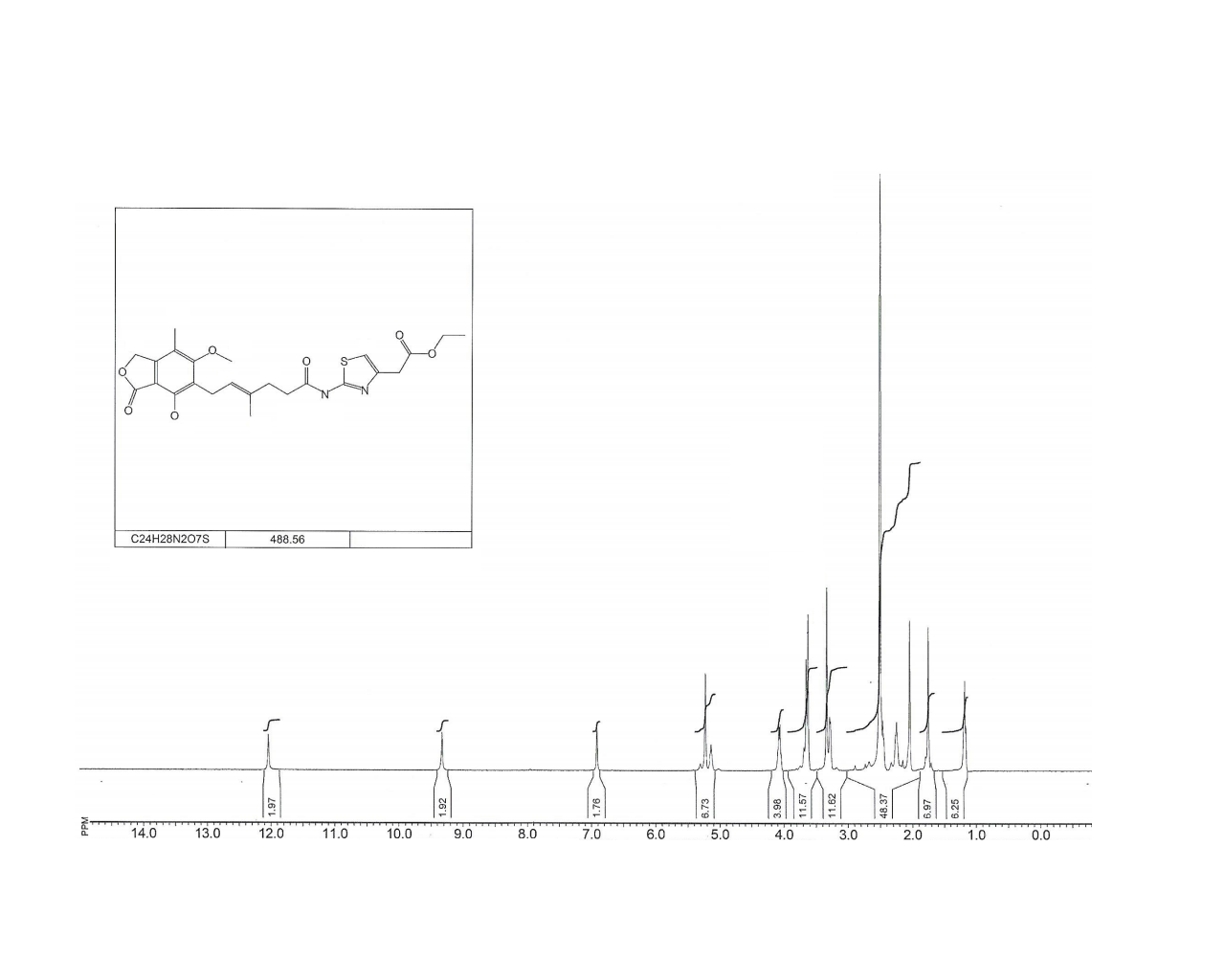

Supplement: Supplementary file 1 [file molecules-23-01621-s001.zip › Supplementary/H-NMR/2-76151 600.tif]

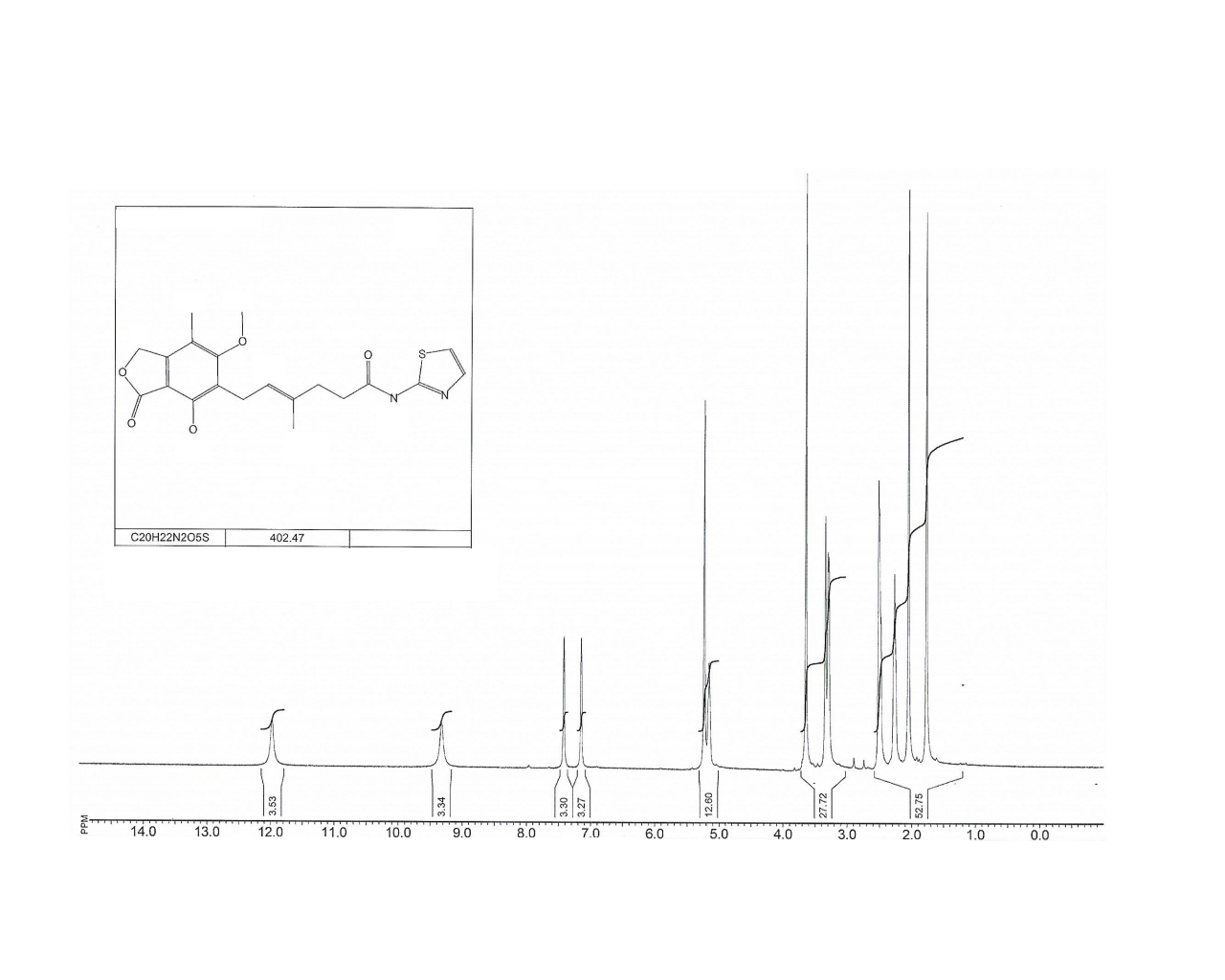

Supplement: Supplementary file 1 [file molecules-23-01621-s001.zip › Supplementary/H-NMR/3-76384 600.tif]

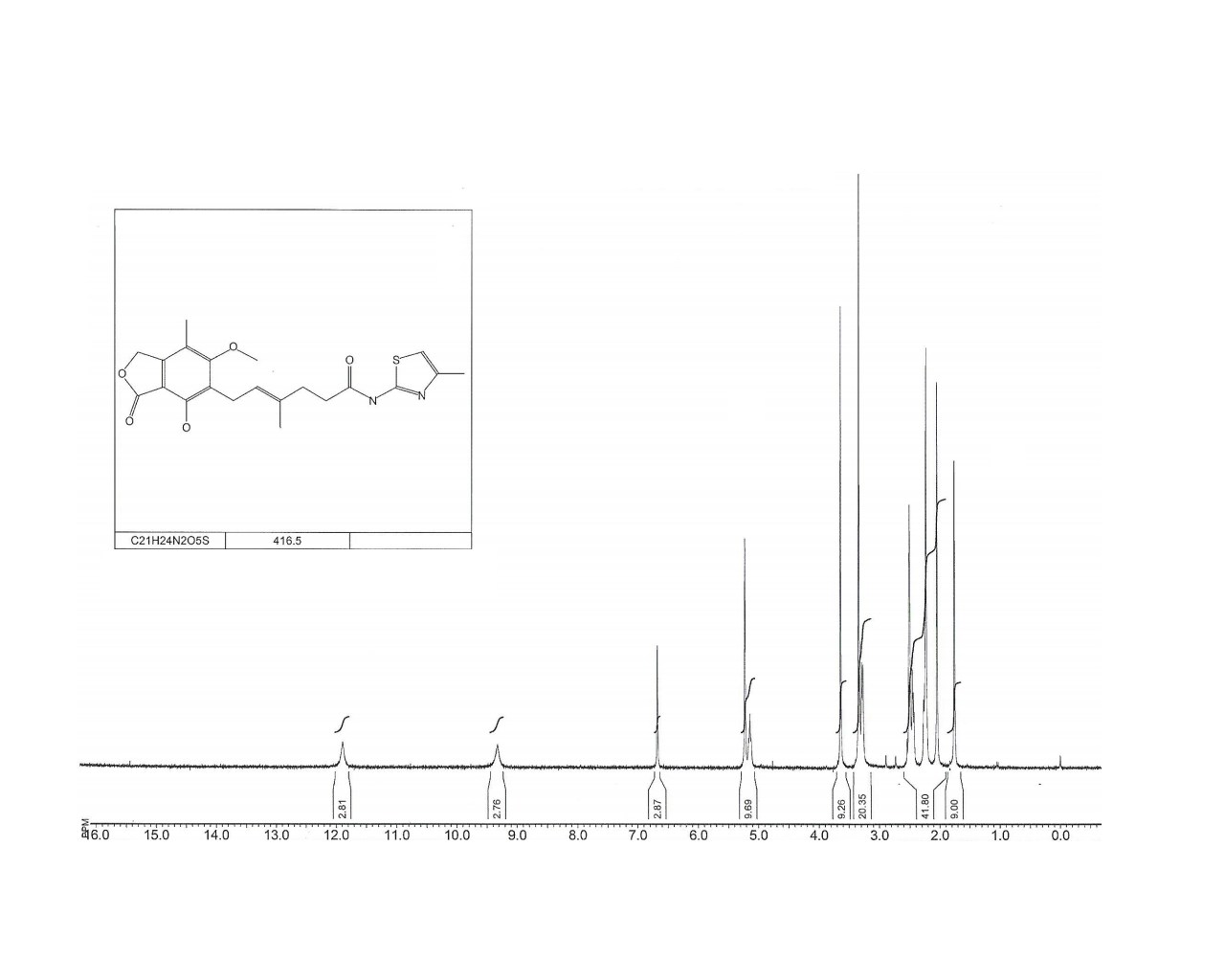

Supplement: Supplementary file 1 [file molecules-23-01621-s001.zip › Supplementary/H-NMR/4-76361 600.tif]

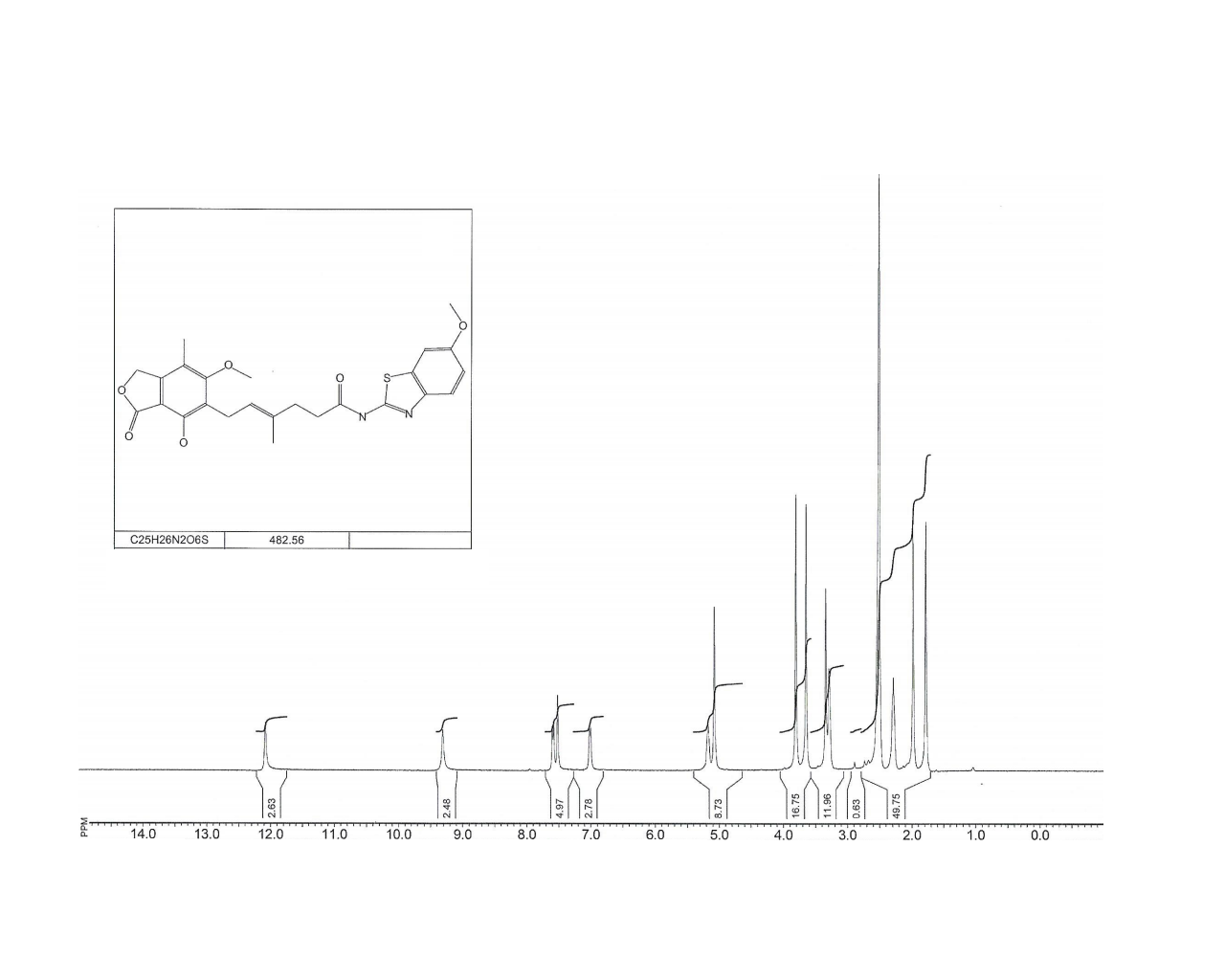

Supplement: Supplementary file 1 [file molecules-23-01621-s001.zip › Supplementary/H-NMR/5-76105 600.tif]

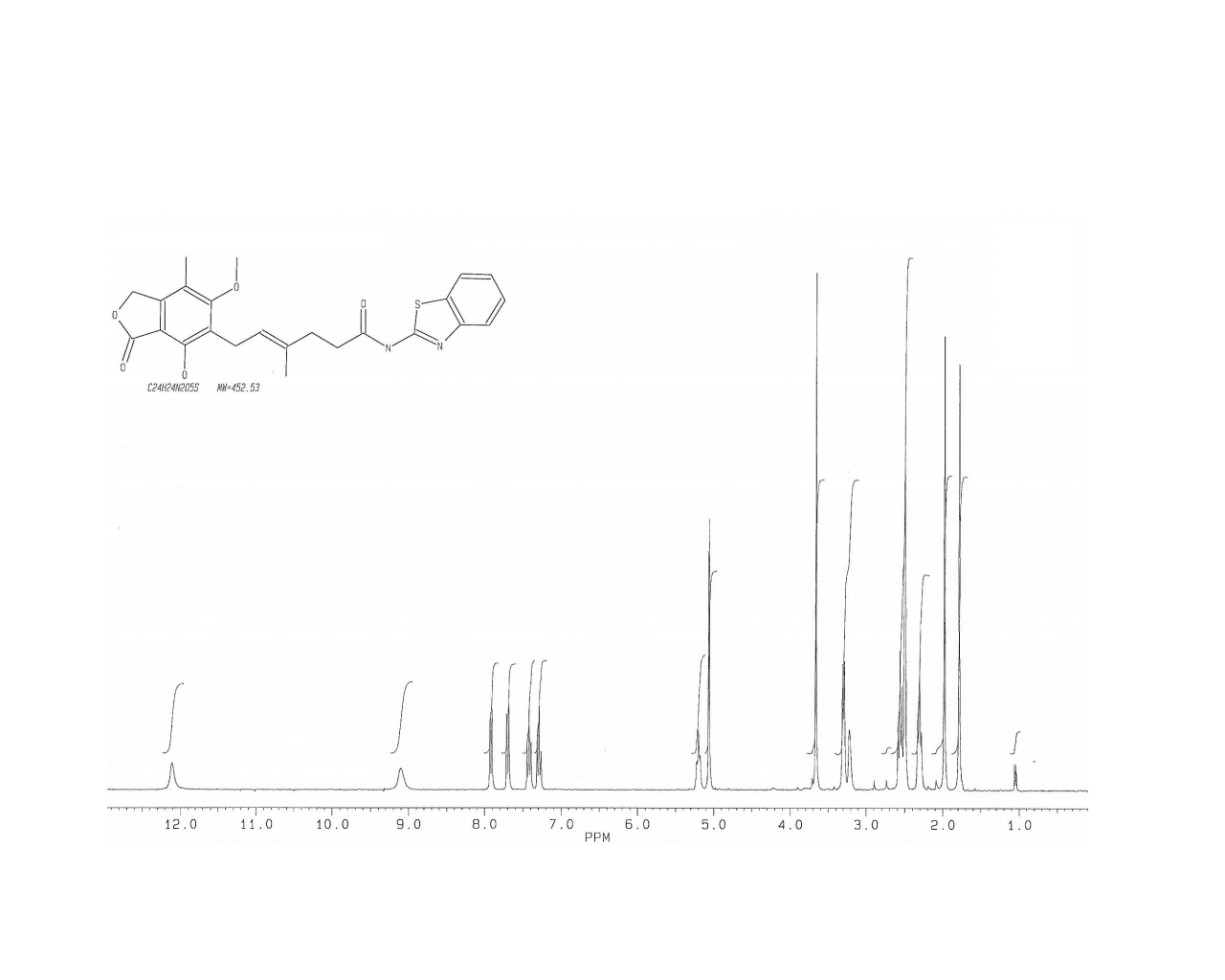

Supplement: Supplementary file 1 [file molecules-23-01621-s001.zip › Supplementary/H-NMR/6-76142 600.tif]

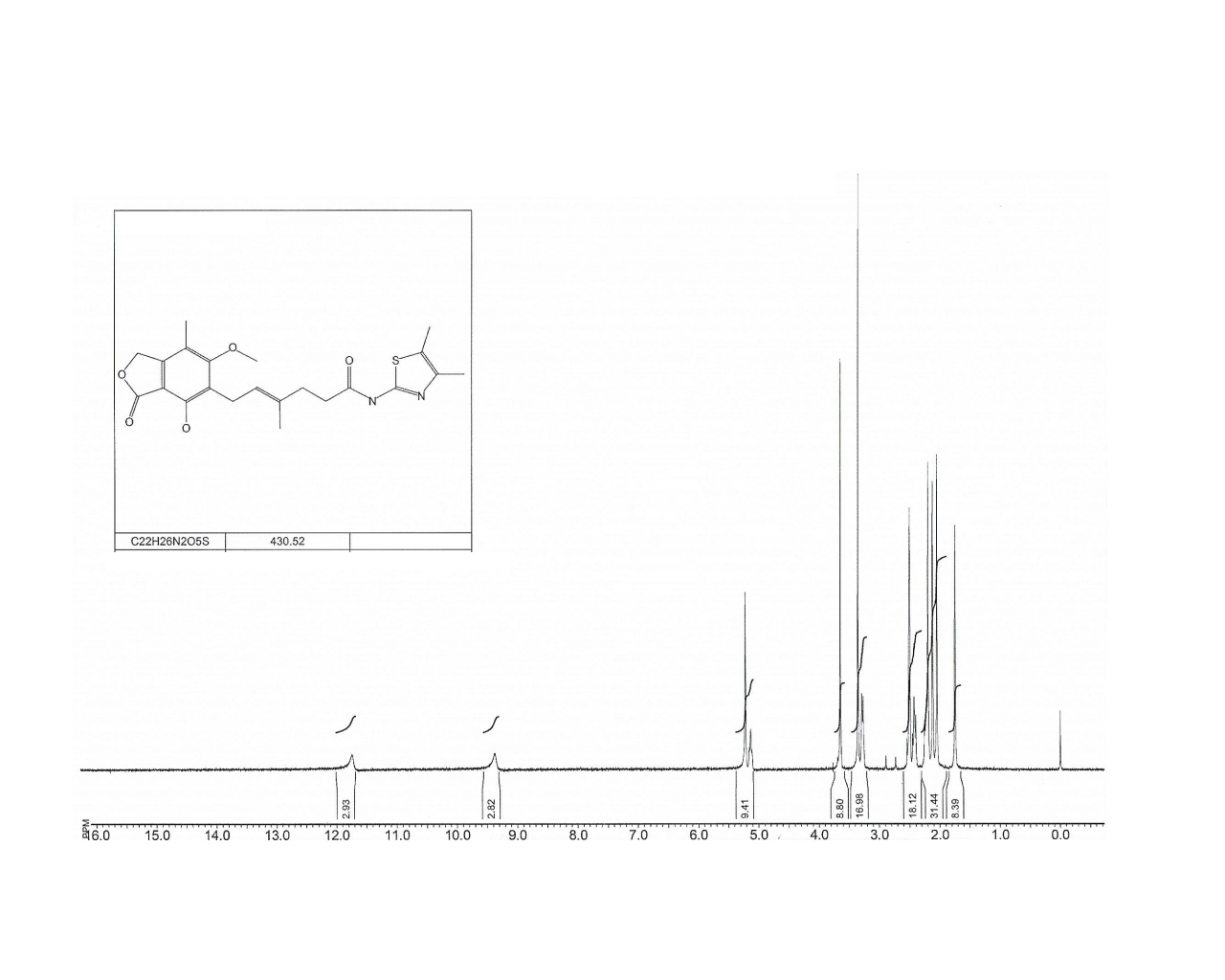

Supplement: Supplementary file 1 [file molecules-23-01621-s001.zip › Supplementary/H-NMR/7-76281 600.tif]

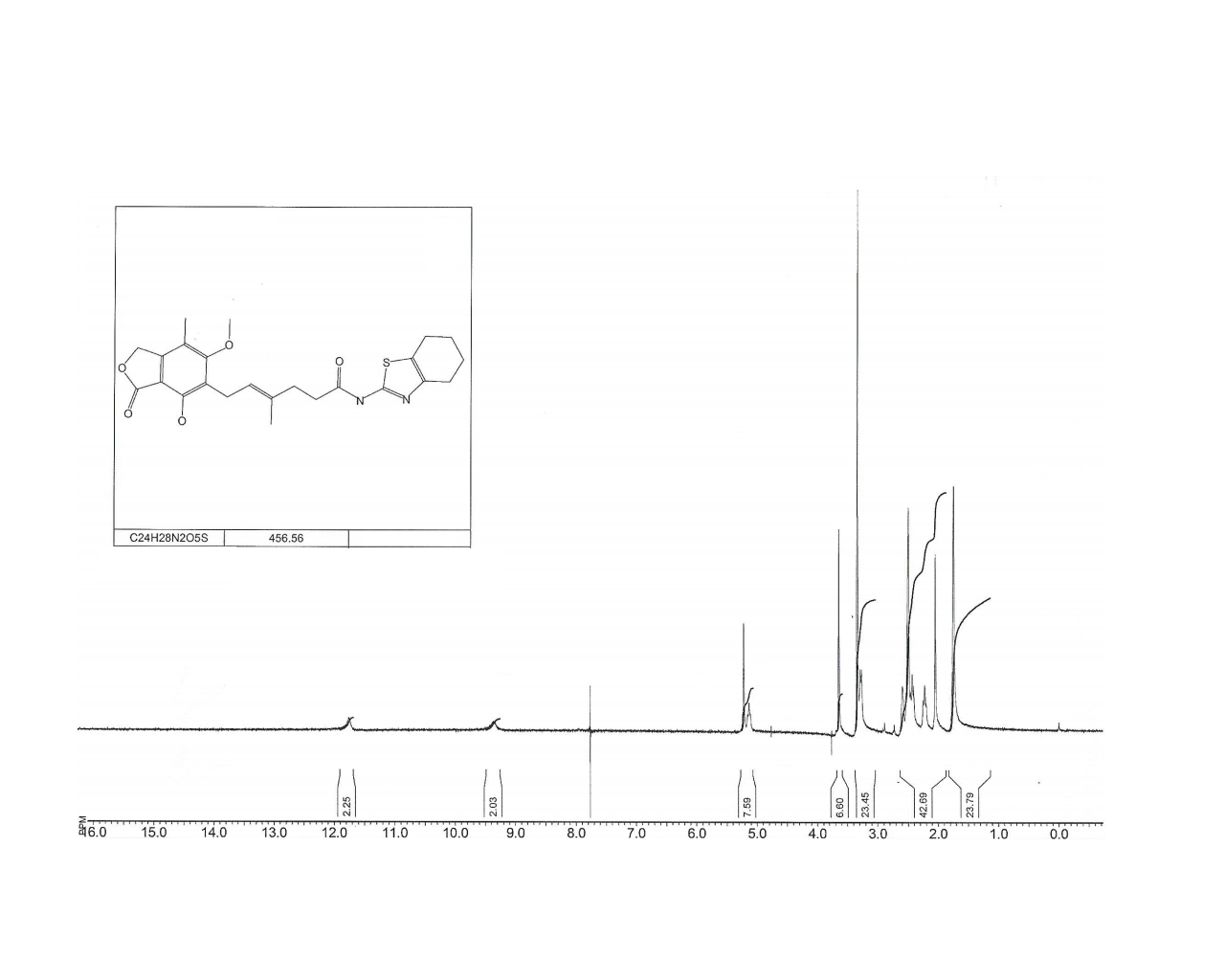

Supplement: Supplementary file 1 [file molecules-23-01621-s001.zip › Supplementary/H-NMR/8-76460 600.tif]

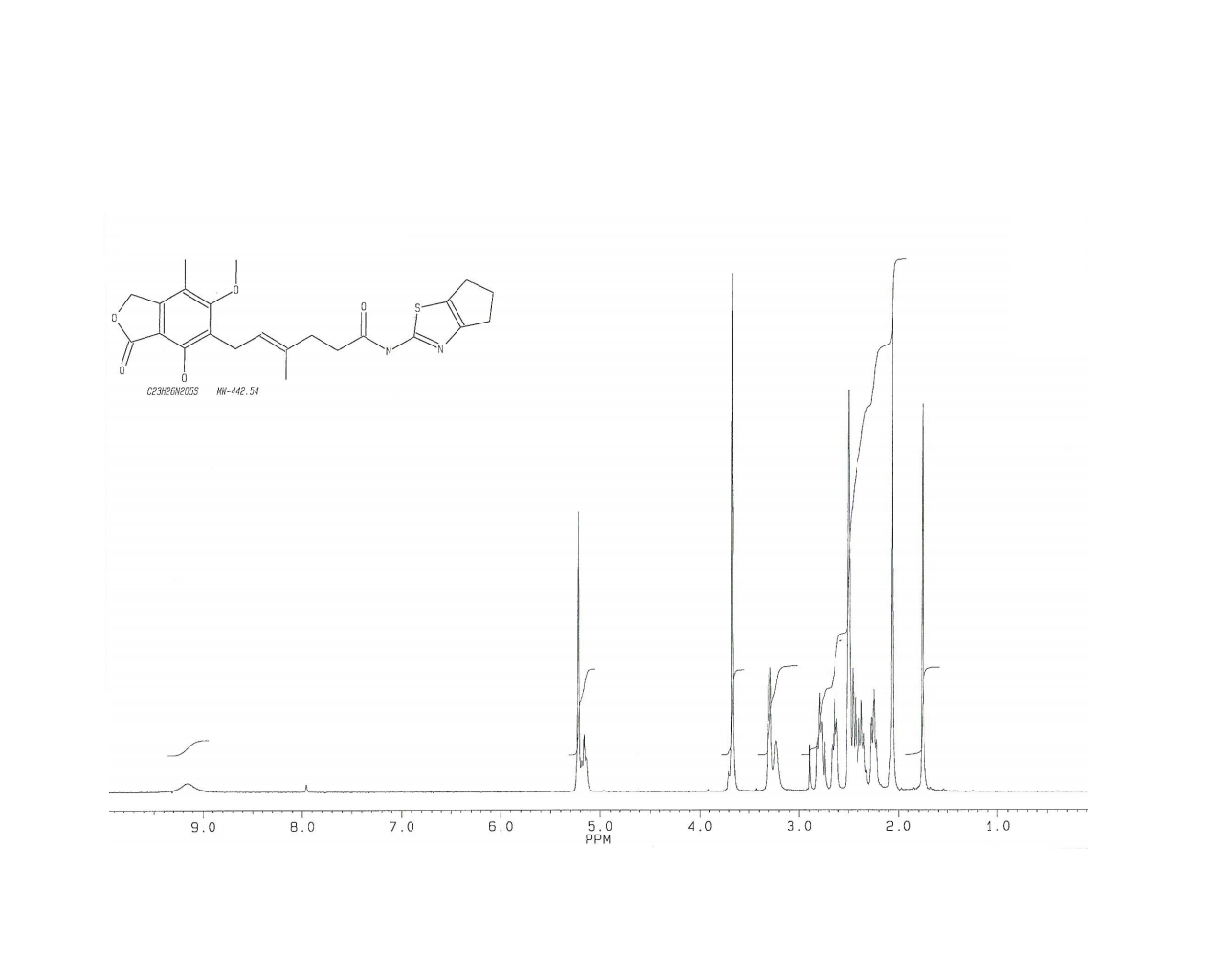

Supplement: Supplementary file 1 [file molecules-23-01621-s001.zip › Supplementary/H-NMR/9-76574 600.tif]

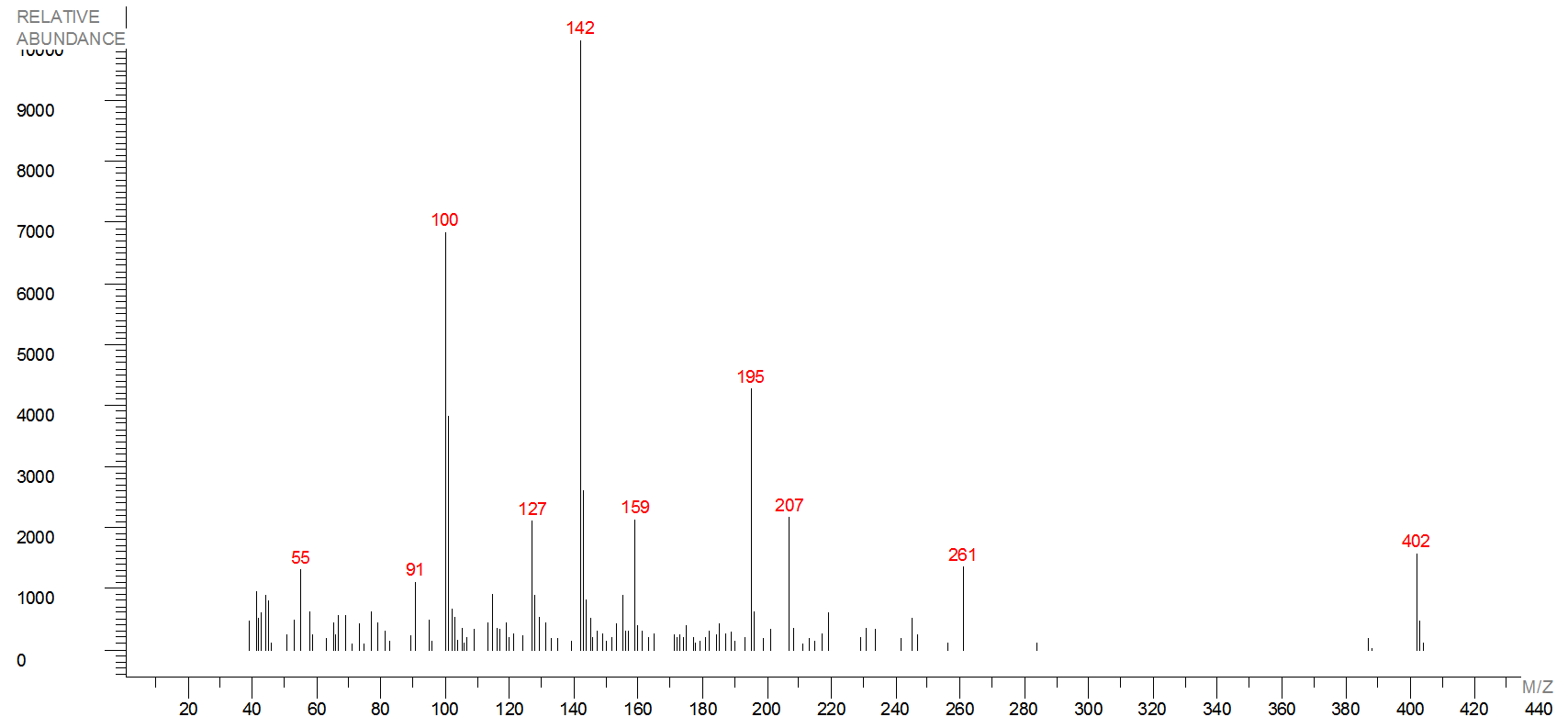

Supplement: Supplementary file 1 [file molecules-23-01621-s001.zip › Supplementary/MS/1.bmp]

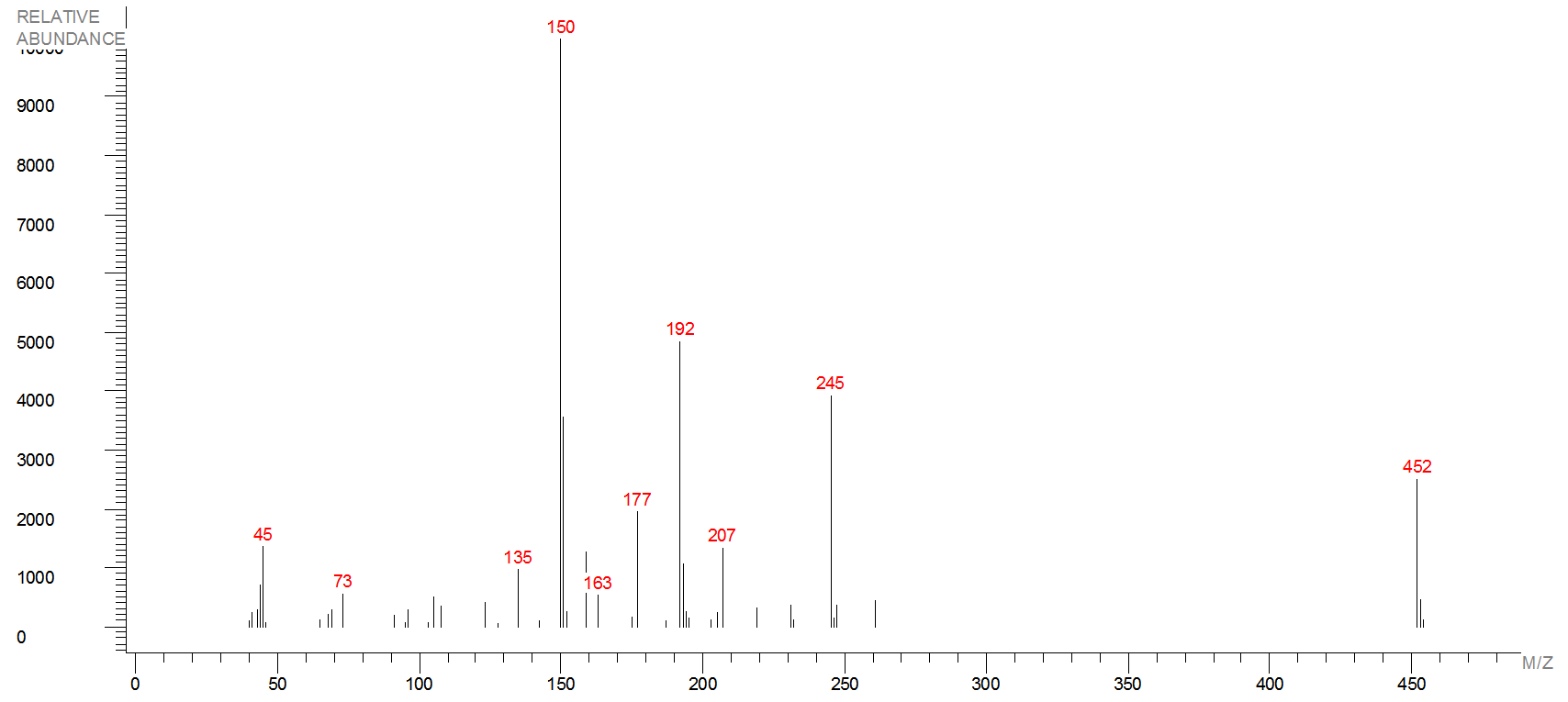

Supplement: Supplementary file 1 [file molecules-23-01621-s001.zip › Supplementary/MS/10.bmp]

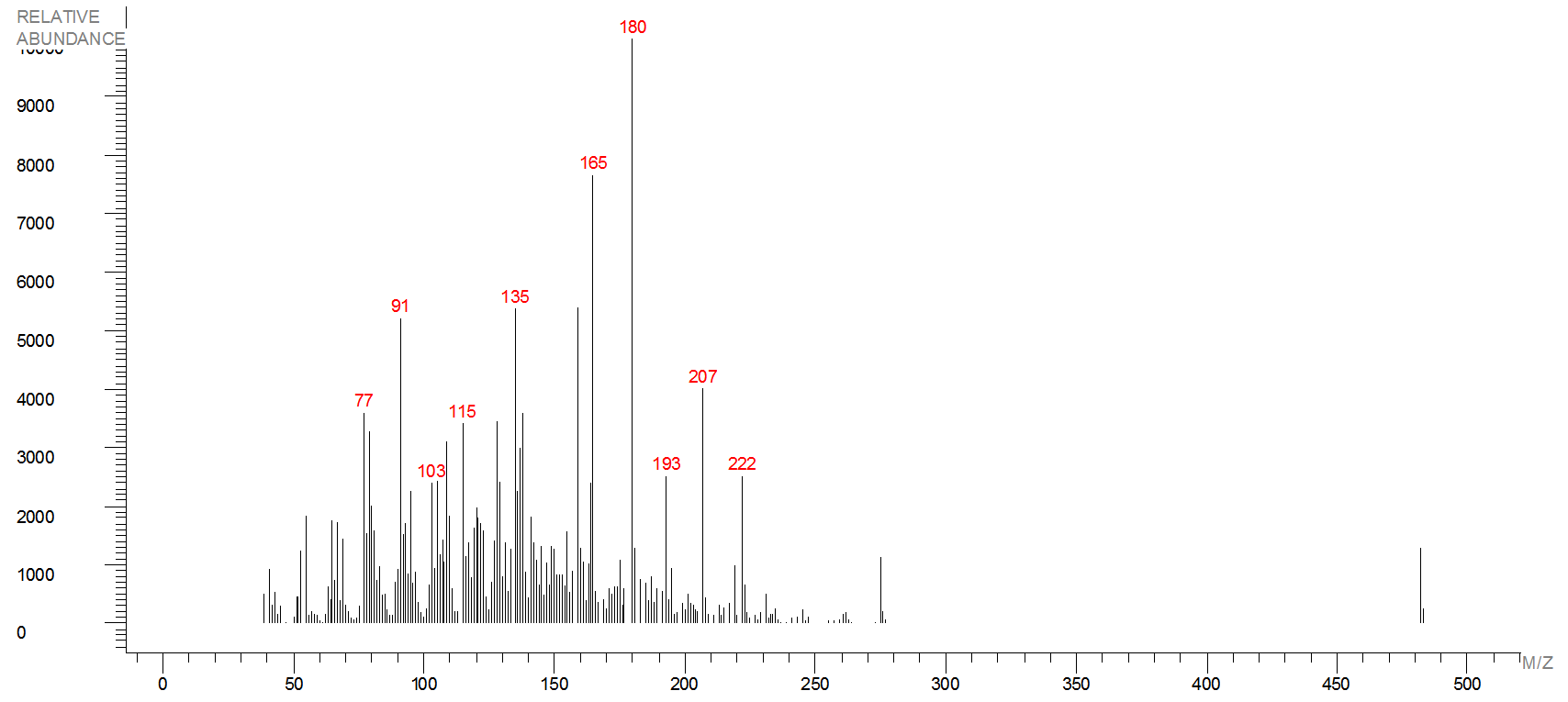

Supplement: Supplementary file 1 [file molecules-23-01621-s001.zip › Supplementary/MS/11.bmp]

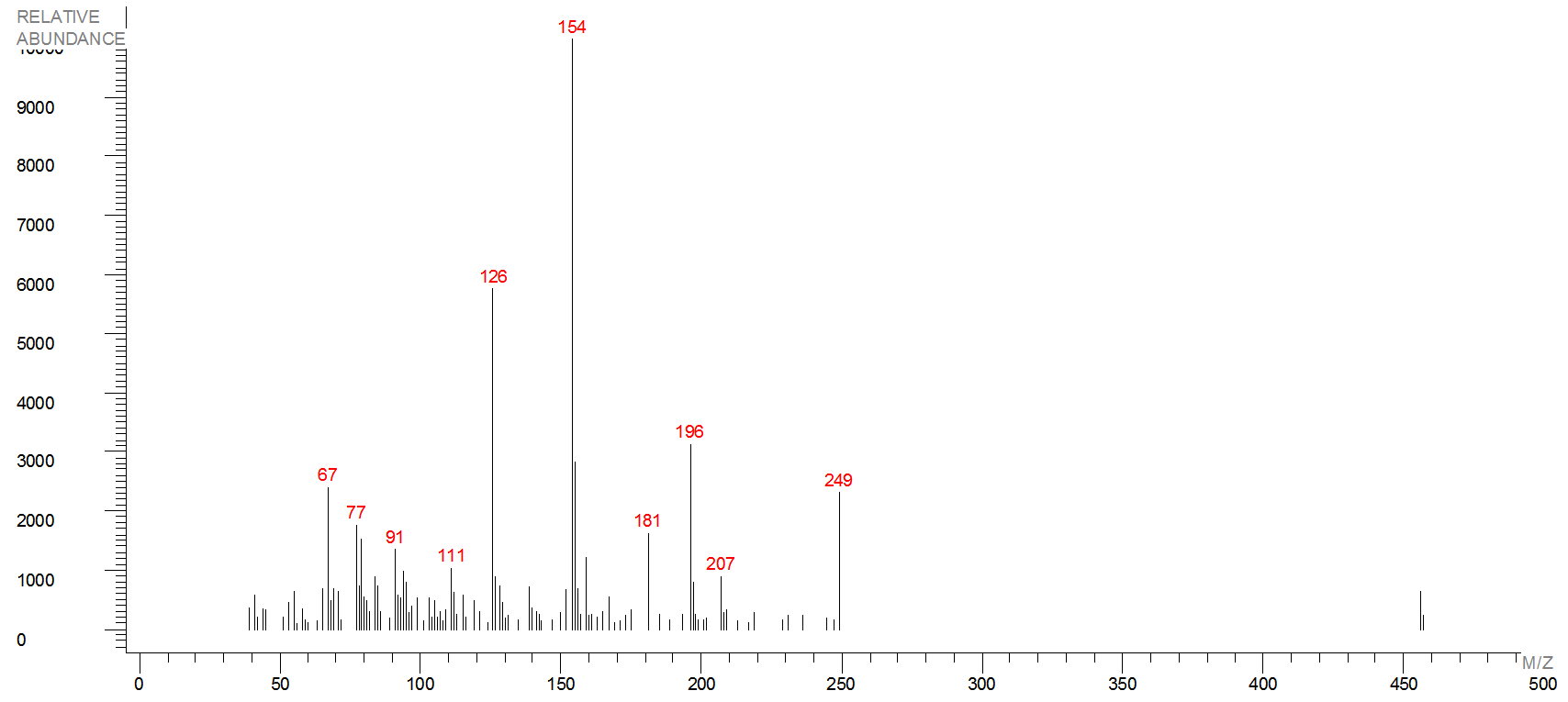

Supplement: Supplementary file 1 [file molecules-23-01621-s001.zip › Supplementary/MS/12.bmp]

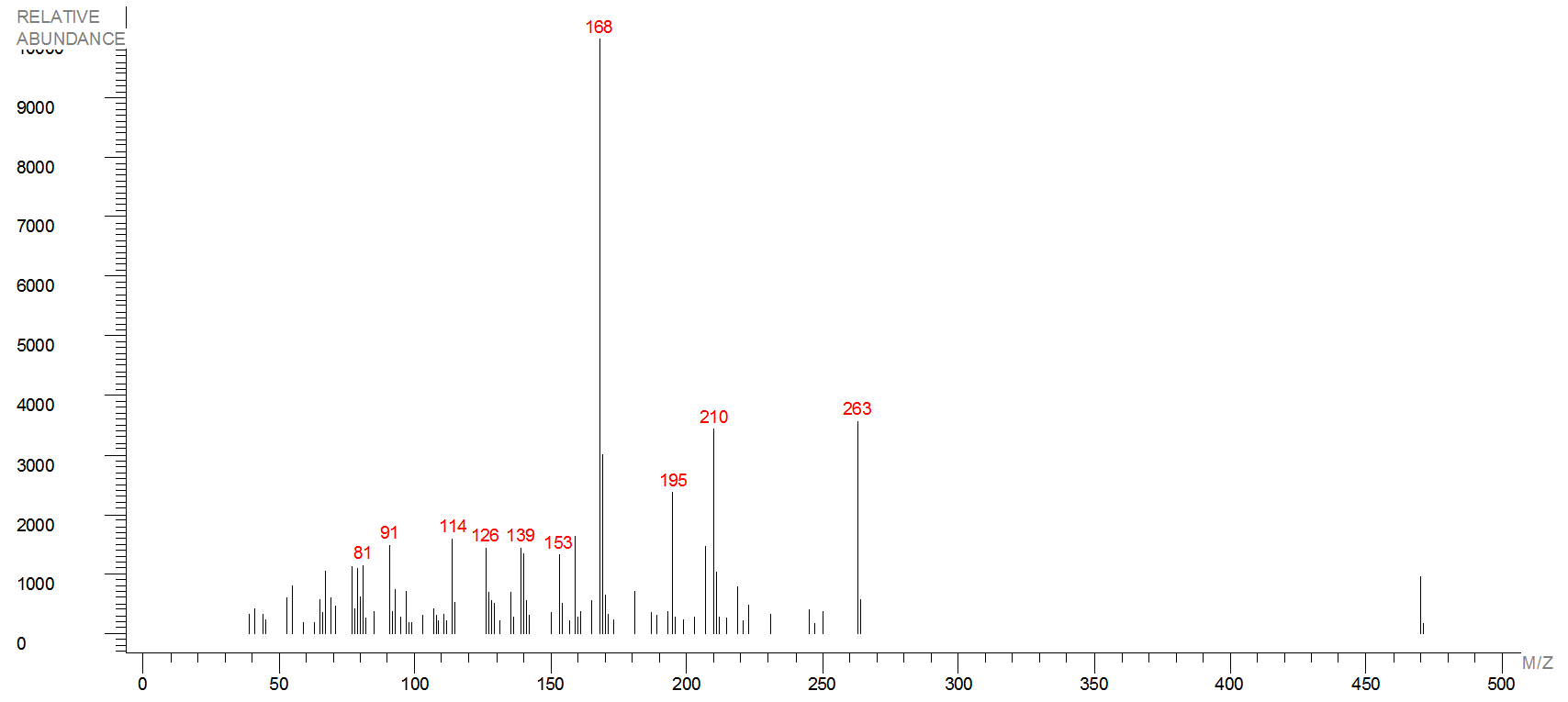

Supplement: Supplementary file 1 [file molecules-23-01621-s001.zip › Supplementary/MS/13.bmp]

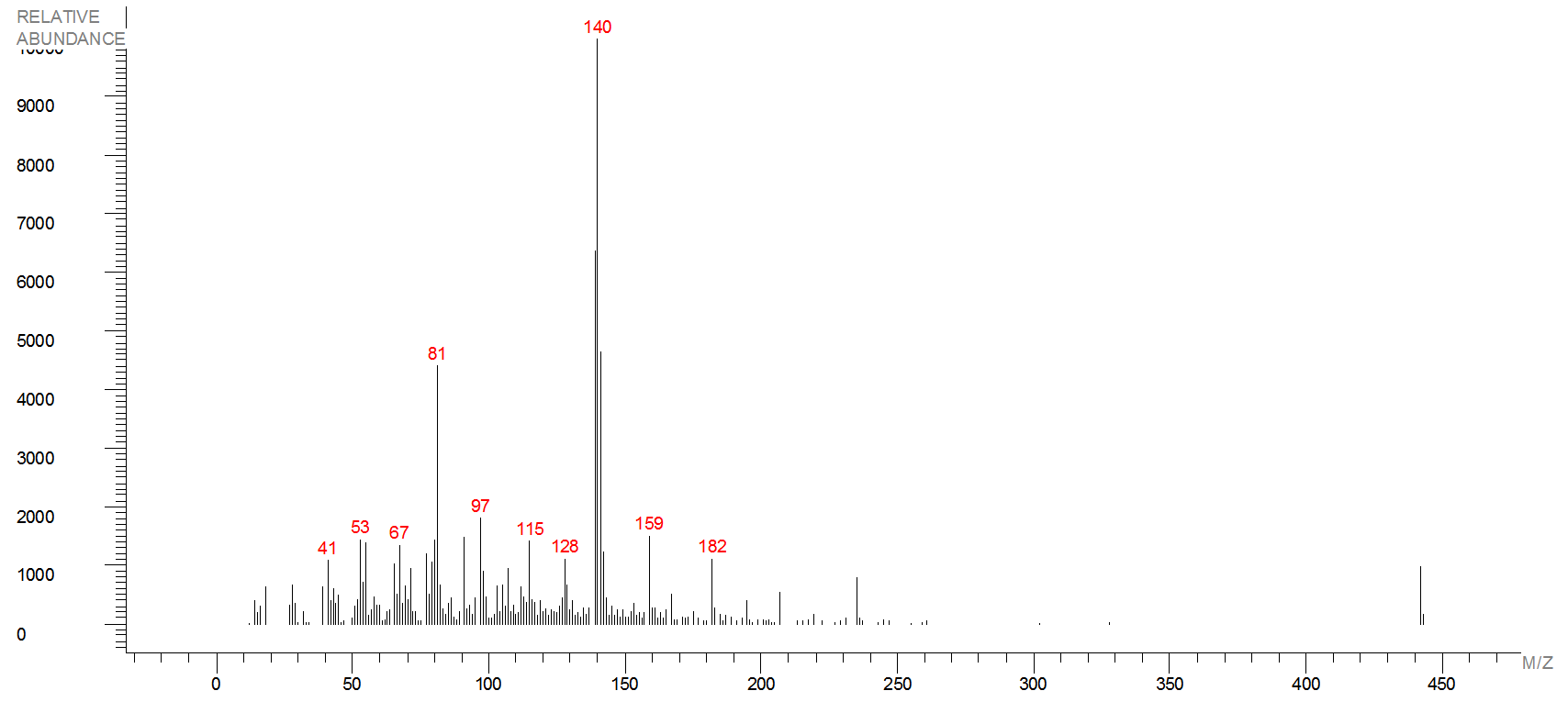

Supplement: Supplementary file 1 [file molecules-23-01621-s001.zip › Supplementary/MS/14.bmp]

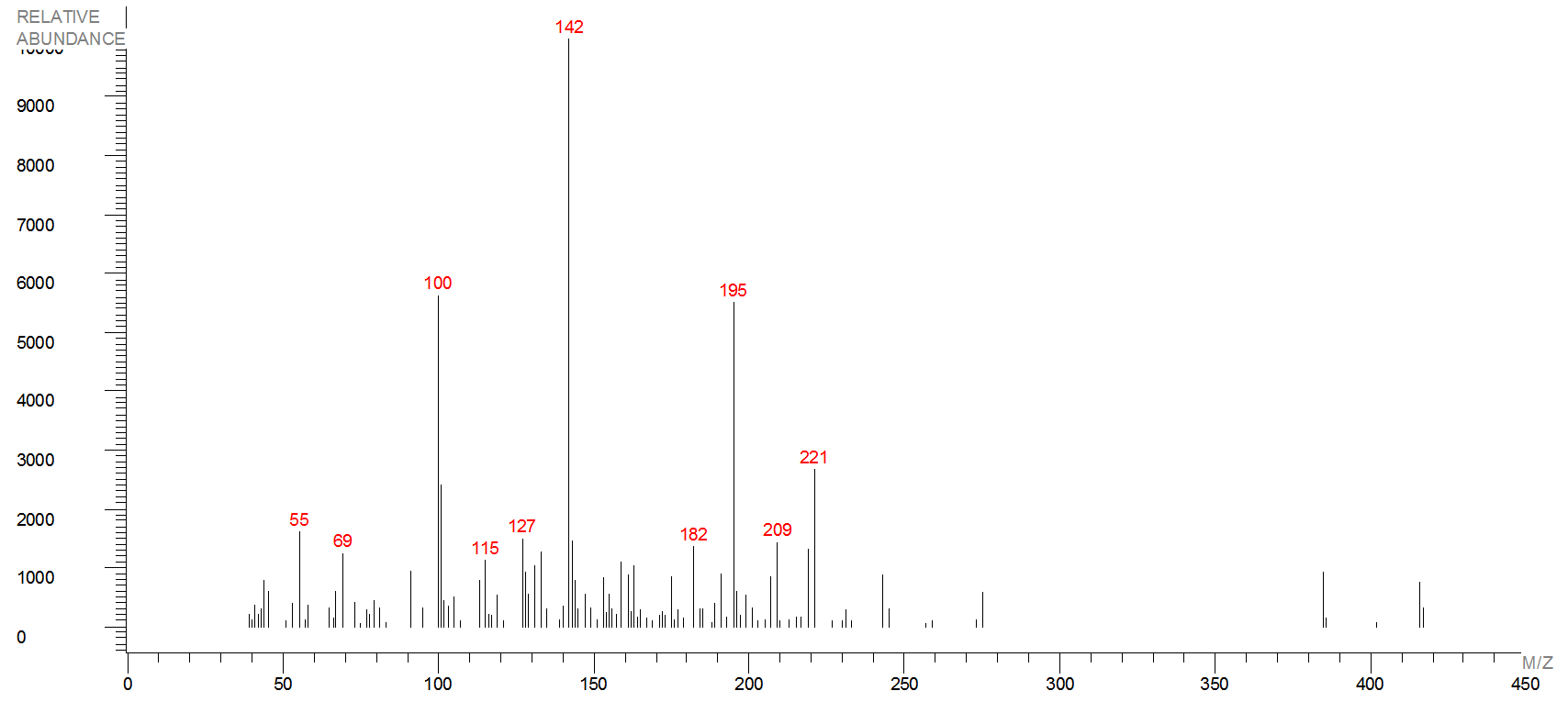

Supplement: Supplementary file 1 [file molecules-23-01621-s001.zip › Supplementary/MS/15.bmp]

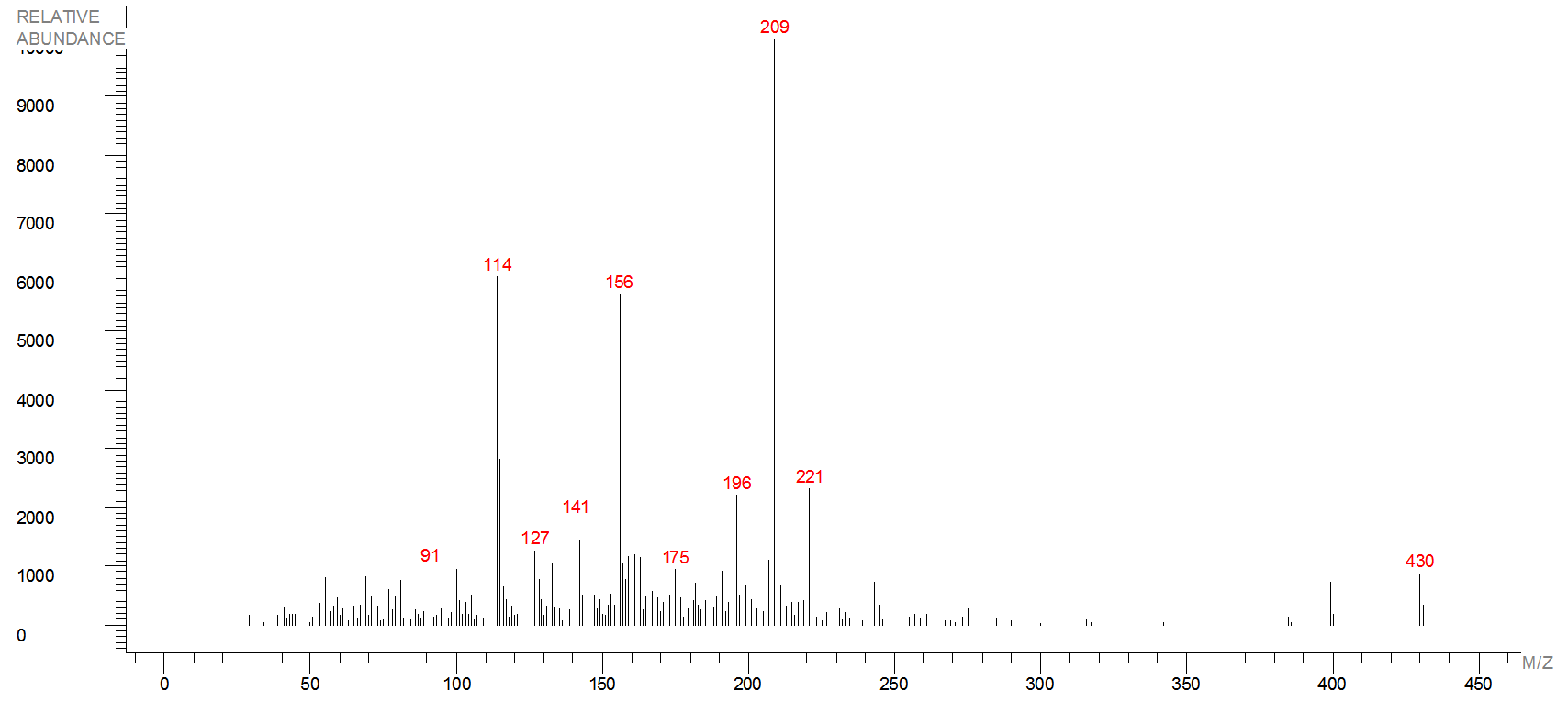

Supplement: Supplementary file 1 [file molecules-23-01621-s001.zip › Supplementary/MS/16.bmp]

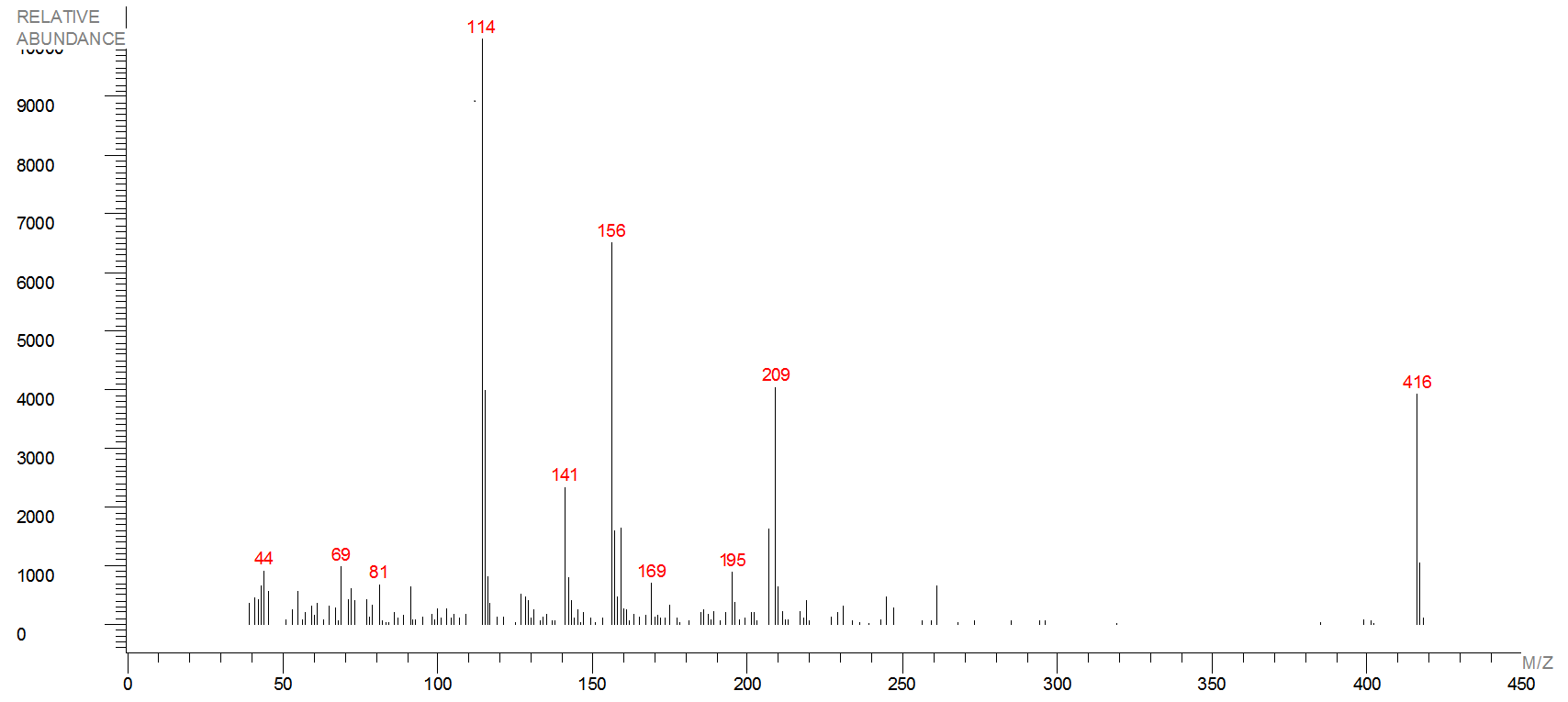

Supplement: Supplementary file 1 [file molecules-23-01621-s001.zip › Supplementary/MS/2.bmp]

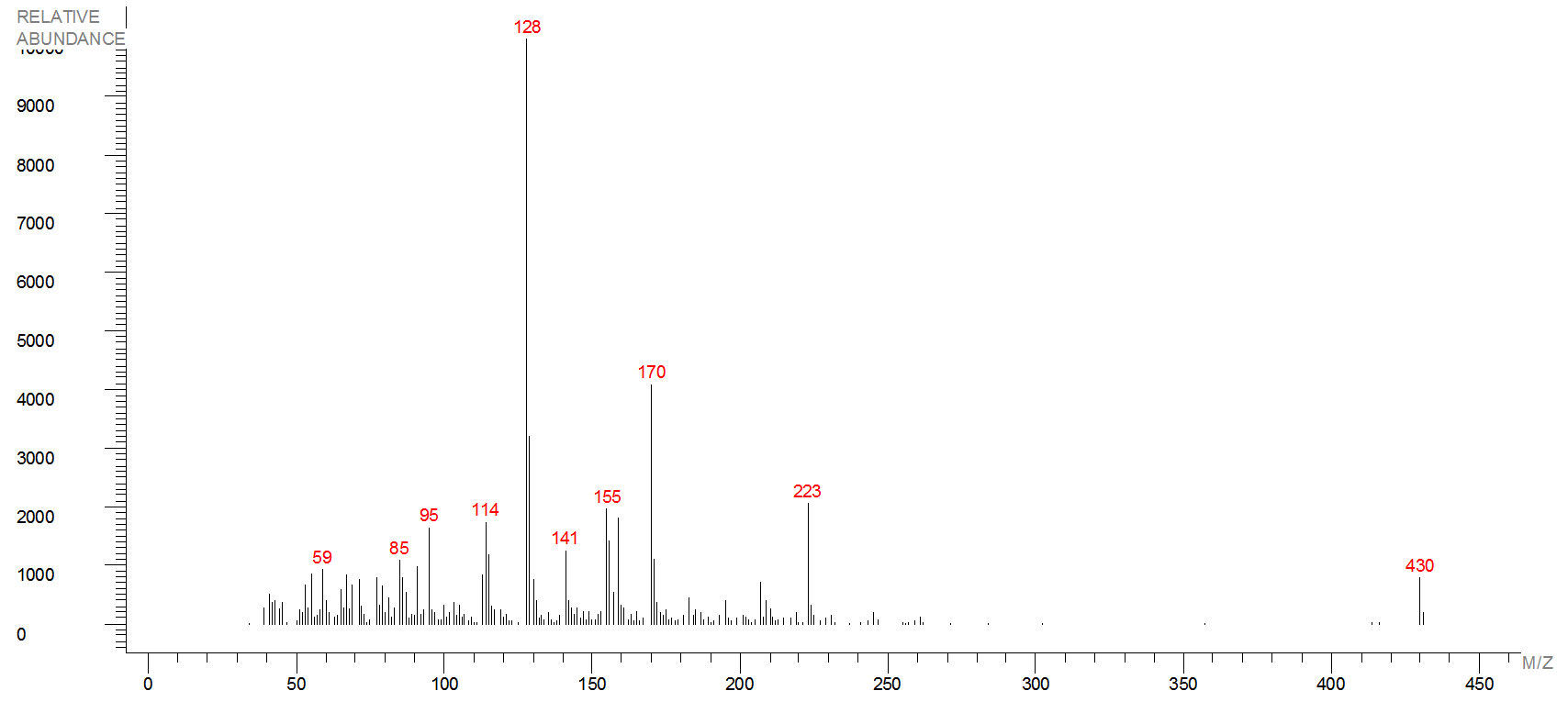

Supplement: Supplementary file 1 [file molecules-23-01621-s001.zip › Supplementary/MS/3.bmp]

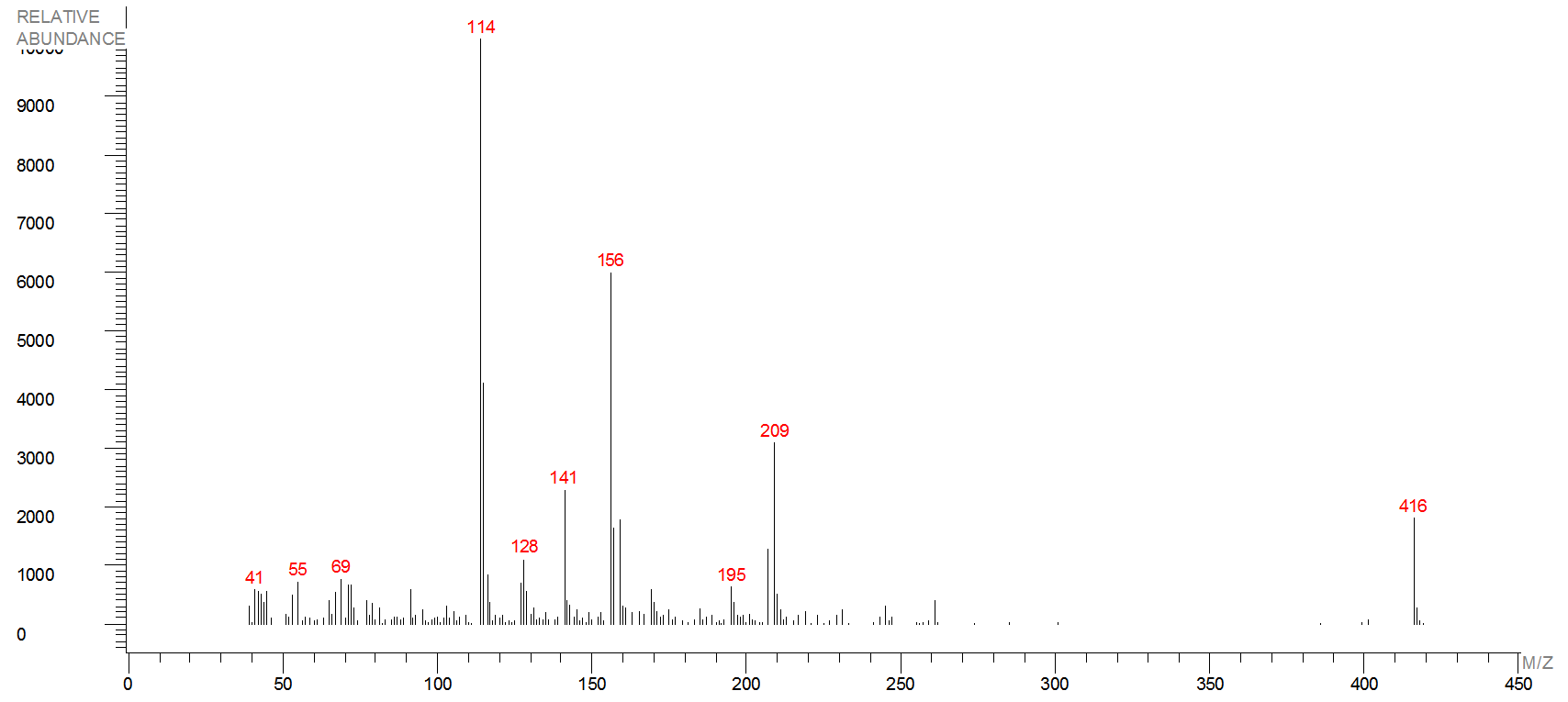

Supplement: Supplementary file 1 [file molecules-23-01621-s001.zip › Supplementary/MS/4.bmp]

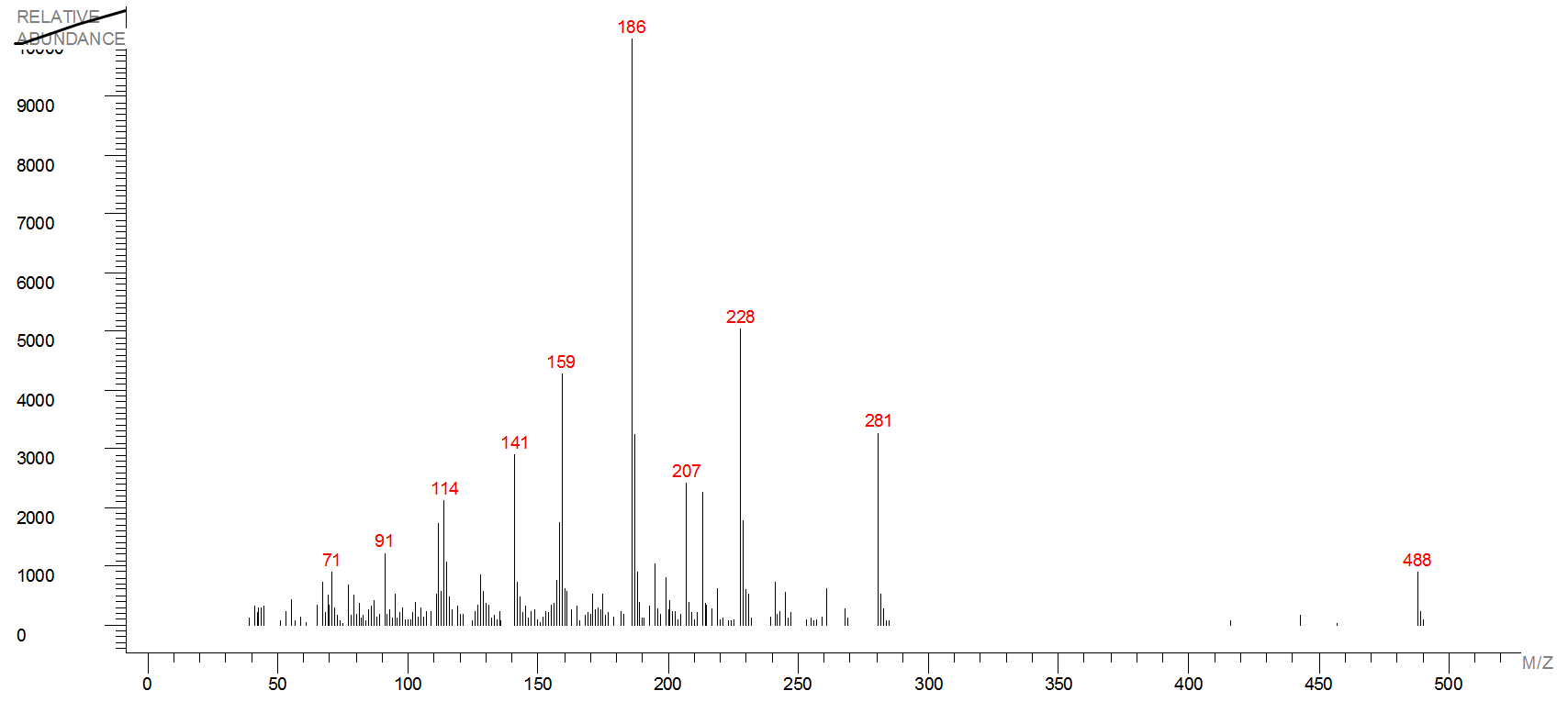

Supplement: Supplementary file 1 [file molecules-23-01621-s001.zip › Supplementary/MS/5.bmp]

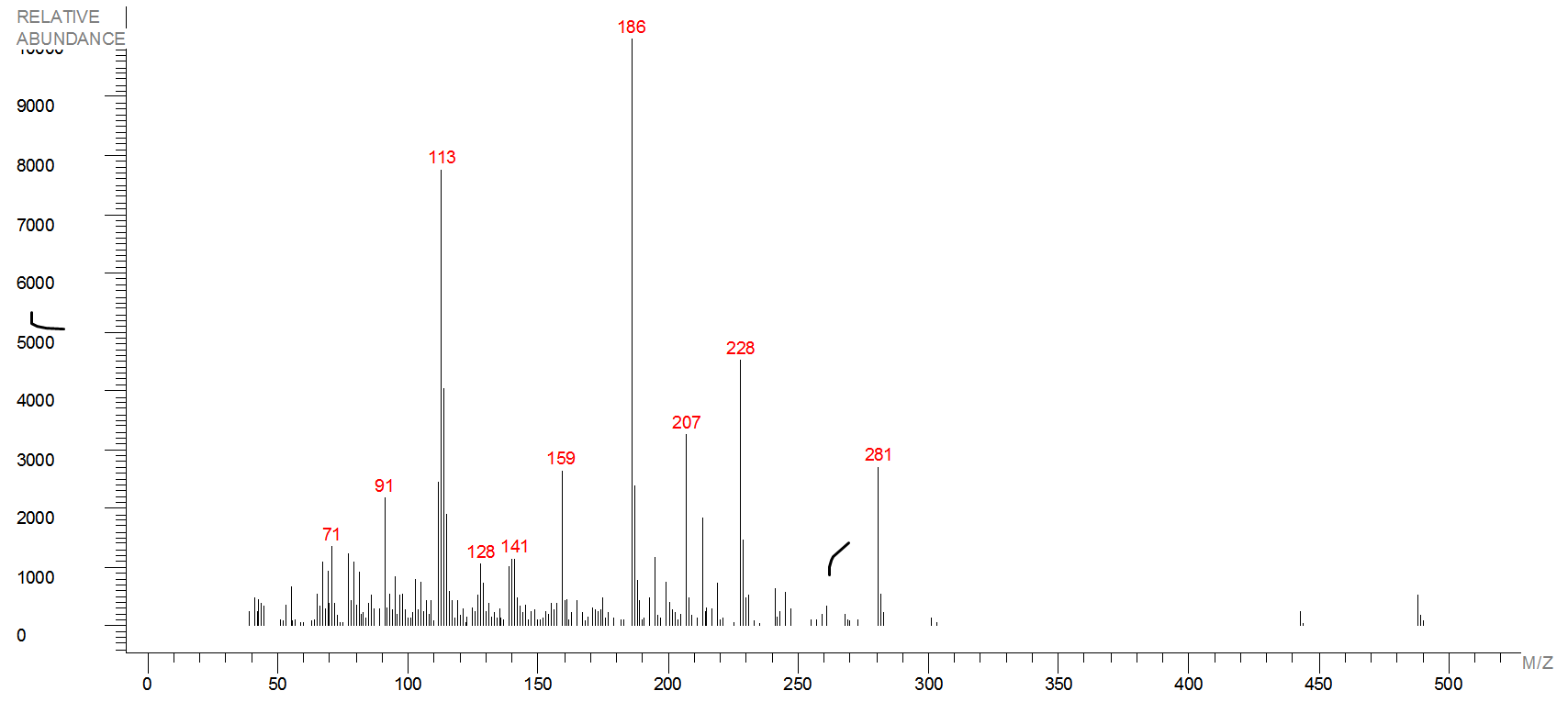

Supplement: Supplementary file 1 [file molecules-23-01621-s001.zip › Supplementary/MS/6.bmp]

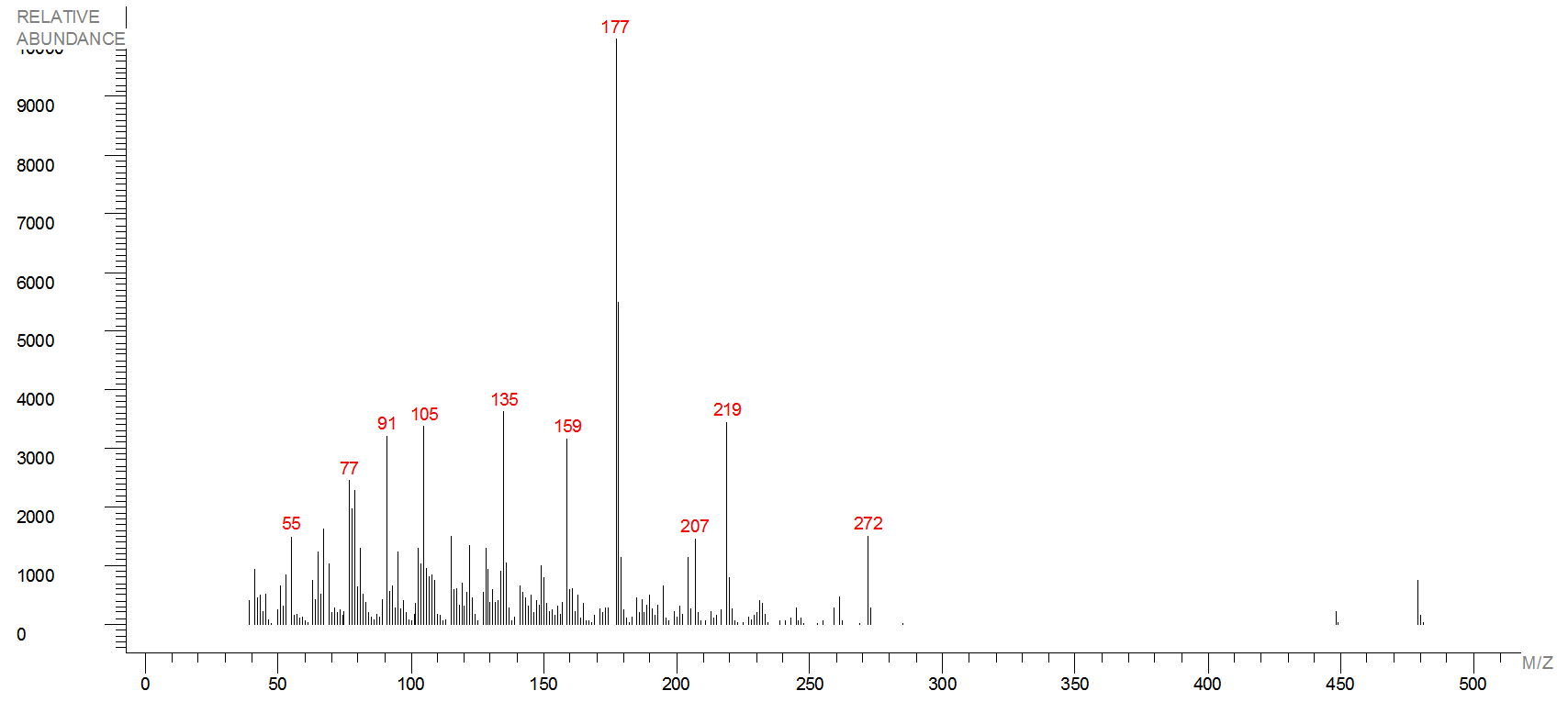

Supplement: Supplementary file 1 [file molecules-23-01621-s001.zip › Supplementary/MS/7.bmp]

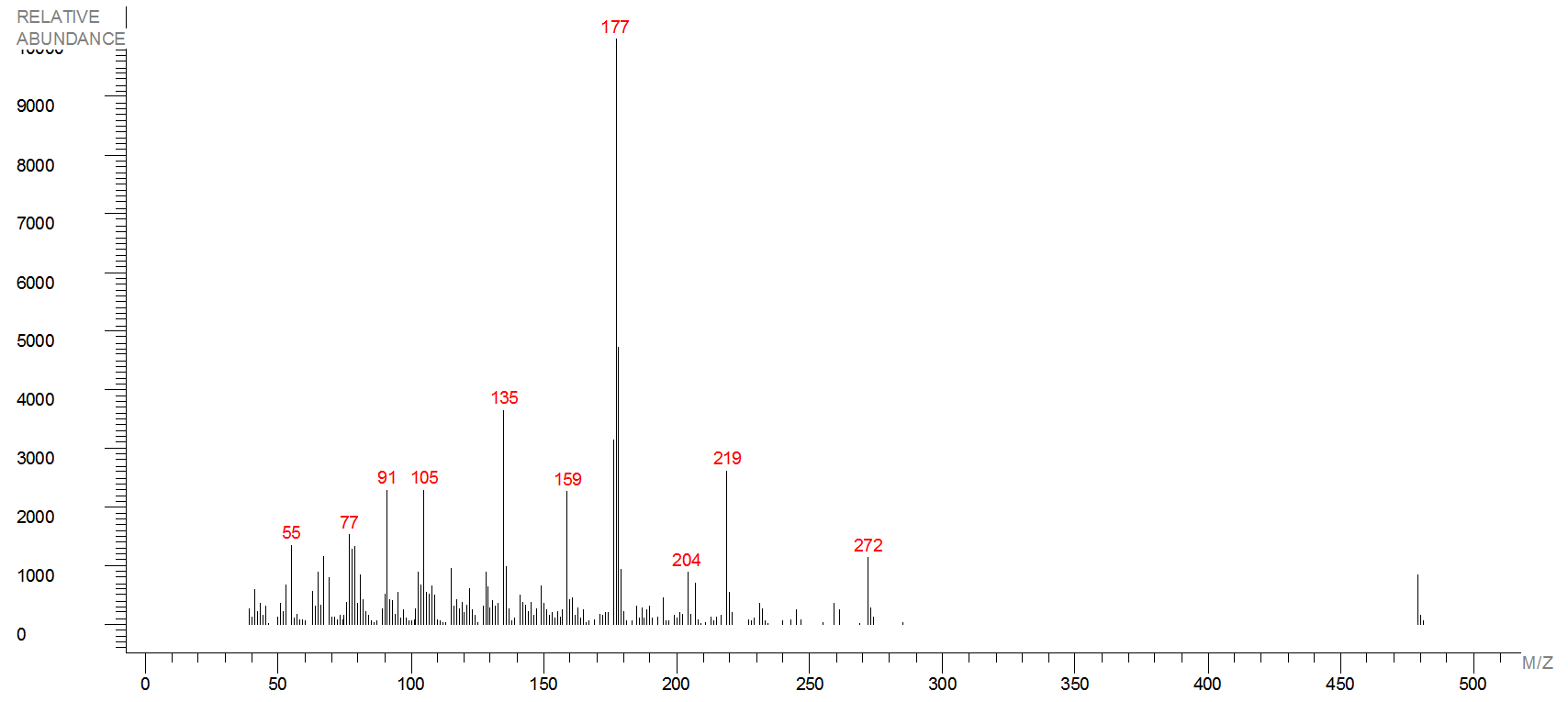

Supplement: Supplementary file 1 [file molecules-23-01621-s001.zip › Supplementary/MS/8.bmp]

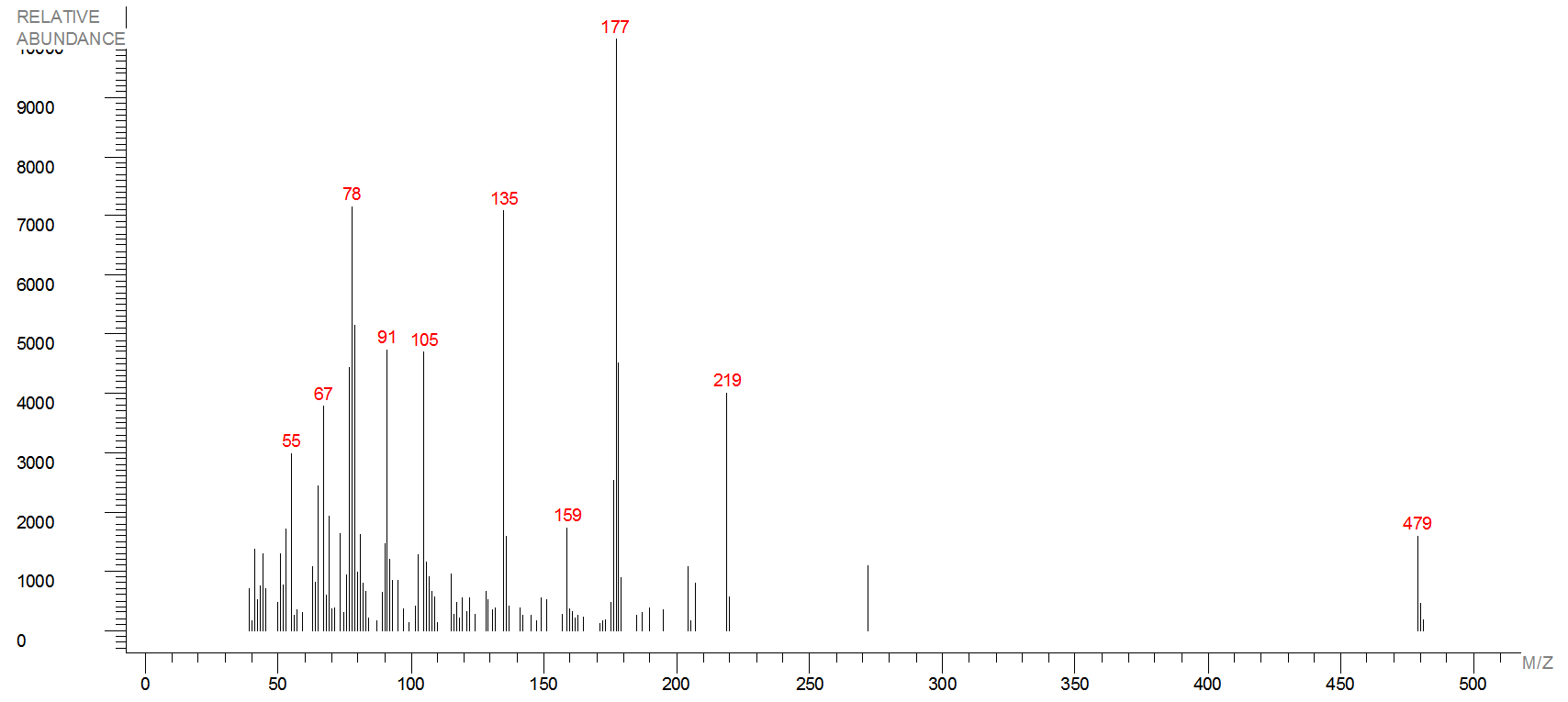

Supplement: Supplementary file 1 [file molecules-23-01621-s001.zip › Supplementary/MS/9.bmp]
